# Phylogenetic analysis of phytochrome A gene from *Lablab purpureus* (L.) Sweet

**DOI:** 10.1186/s43141-021-00295-z

**Published:** 2022-01-13

**Authors:** Stuti Krishna, Kaushal Modha, Vipulkumar Parekh, Ritesh Patel, Digvijay Chauhan

**Affiliations:** 1 Department of Genetics and Plant Breeding, N. M. College of Agriculture, Navsari Agricultural University, Navsari, Gujarat 396 450 India; 2 Department of Basic Science and Humanities, ASPEE College of Horticulture and Forestry, NAU, Navsari, Gujarat 396 450 India; 3 Pulses and Castor Research Station, Navsari Agricultural University, Navsari, Gujarat 396 450 India

**Keywords:** Phytochrome, *PHYA3*, GAF, PAS, Phylogenetic analysis

## Abstract

**Background:**

Phytochromes are the best characterized photoreceptors that perceive Red (R)/Far-Red (FR) signals and mediate key developmental responses in plants. It is well established that photoperiodic control of flowering is regulated by *PHY A* (phytochrome A) gene. So far, the members of *PHY A* gene family remains unexplored in *Lablab purpureus*, and therefore, their functions are still not deciphered. *PHYA3* is the homologue of phytochrome A and known to be involved in dominant suppression of flowering under long day conditions by downregulating florigens in *Glycine max*. The present study is the first effort to identify and characterize any photoreceptor gene (*PHYA3*, in this study) in *Lablab purpureus* and decipher its phylogeny with related legumes.

**Results:**

*PHYA3* was amplified in *Lablab purpureus* cv GNIB-21 (photo-insensitive and determinate) by utilizing primers designed from *GmPHYA3* locus of *Glycine max*. This study was successful in partially characterizing *PHYA3* in *Lablab purpureus* (*LprPHYA3*) which is 2 kb longer and belongs to exon 1 region of *PHYA3* gene. Phylogenetic analysis of the nucleotide and protein sequences of *PHYA* genes through MEGA X delineated the conservation and evolution of *Lablab purpureus PHYA3* (*LprPHYA3*) probably from *PHYA* genes of *Vigna unguiculata*, *Glycine max* and *Vigna angularis*. A conserved basic helix-loop-helix motif bHLH69 was predicted having DNA binding property. Domain analysis of *GmPHYA* protein and predicted partial protein sequence corresponding to exon-1 of *LprPHYA3* revealed the presence of conserved domains (GAF and PAS domains) in *Lablab purpureus* similar to *Glycine max*.

**Conclusion:**

Partial characterization of *LprPHYA3* would facilitate the identification of complete gene in *Lablab purpureus* utilizing sequence information from phylogenetically related species of Fabaceae. This would allow screening of allelic variants for *LprPHYA3* locus and their role in photoperiod responsive flowering. The present study could aid in modulating photoperiod responsive flowering in *Lablab purpureus* and other related legumes in near future through genome editing.

**Supplementary Information:**

The online version contains supplementary material available at 10.1186/s43141-021-00295-z.

## Background

Photoperiod sensitivity is an important trait as it enables crops to adapt to different latitudes. Short-day (SD) crops like *Oryza sativa*, *Lablab purpureus*, *Phaseolus vulgaris* and *Glycine max* requires photoperiod insensitivity to adapt to a high latitudinal environment [1]. Photoperiod is governed by phytochrome genes which play critical role in responding to the light quantity, quality and periodicity in plants and make communication between different biochemicals signaling pathway for growth and development of crops [2]. Phytochromes are Red (R)/Far-Red (FR) light receptors involving interconversion of inactive R (Pr) to active FR (Pfr) form by red light absorption which triggers its transfer to the nucleus and thus guides gene expression [3]. Phytochrome is the best characterized photoreceptor and its apoproteins are encoded by small multigene families. In recent decade, progress has been made in characterizing the number, molecular properties and biological activity of the photoreceptors that comprise a plant R/FR detecting framework [2].

Legumes have been documented with vast genetic variation for flowering and photoperiod has been quite involved in governing growth habit, flowering and maturity in these crops [4]. Legumes have been the focus of presently expanding genomics research and the availability of vast genomic resources and established synteny within the Fabaceae family has enabled the identification of candidate genes for flowering in other related species and exploring their molecular physiologies involved in downstream processes [5]. The fundamental genes governing flowering pathway in legumes are conserved from model plant *Arabidopsis* that contains five *PHY* (phytochrome) genes (*PHYA*, *PHYB*, *PHYC*, *PHYD* and *PHYE*) and each of these gene have crucial role [6, 7]. Different maturity loci, i.e. E-series (*E1* to *E8*) represent a range of allelic composition and each *E* locus affects flowering time and maturity in *Glycine max* [8–14]. Photoperiod sensitivity under different light conditions was found to be related to *E1*, *E3*, *E4* and *E7* [15]. *E3* and *E4* encode *GmPhyA3* and *GmPhyA2*, respectively, which are homologues of photoreceptor phytochrome A [16]. *GmPHYA3* and *GmPHYA2* along with *GmPHYA1*, which is encoded by a homoeologous copy of *E4*, redundantly or complementarily function in floral induction and de-etiolation responses under various light conditions [17]. *E3* and *E4* coordinates flowering responses to long-day (LD) conditions with different Red-to-Far-Red (R:FR) quantum ratios. *E3* responds to light with high R:FR ratio; plants homozygous for the recessive *e3* allele can initiate flowering under the LD conditions generated by fluorescent lamps with a high R:FR ratio [18]. Photoperiod sensitivity and determinacy played important role in evolution of *Phaseolus vulgaris* [19]. *GmFT2a* and *GmFT5a*, two homologues of *FT* (Flowering locus T), are reported to induce photoperiodic flowering in *Glycine max* [20] and they are regulated by *E1*, *E3* (*PHYA3*) and *E4* genes under different photoperiod conditions to induce or repress flowering [15]. Photoperiodic response of *PHYA3* under short day and long day conditions affects growth habit by inducing other genes that activate florigens and guides floral initiation at shoot apex [3].

The research on photoperiod responsive flowering is mainly anchored to few major pulse legumes *viz.*, *Glycine max*, *Pisum sativum*, *Phaseolus vulgaris*, *Cajanus cajan* and vegetable legumes endured despite being a promising nutritional source. Indian bean [*Lablab purpureus* (L.) Sweet] is one such underexploited legume with wide range of uses as vegetable, forage, cover crop, split pulse, fodder and medicinal [21]. The molecular information regarding this crop is scarce; nevertheless, it has the potential to become a model pulse crop in genomics era owing to its immense genetic diversity and adaptability [22]. Most landraces of *Lablab purpureus* are photoperiod sensitive and indeterminate which flower only during short days, while few improved varieties with determinate growth and photoperiod insensitivity are available which flowers within 40 to 50 days across the year. Photoperiod responsive flowering along with growth habit might have played crucial role in domestication and evolution of this crop. Dominant nature of photoperiod sensitivity, indeterminate growth habit and purple flower was reported along with coupling phase of linkage between photoperiod insensitive flowering and determinate growth habit in *Lablab purpureus* [23]. Molecular tagging of photoperiod responsive flowering in *Lablab purpureus* discerned that photoperiod insensitive flowering and determinate growth habit is linked and they might be governed by recessive alleles of *GmPHYA3* and *Dt* homologs, respectively [24]. Most recently, allelic characterization of *TFL* (Terminal Flowering locus) governing growth habit has been reported along with involvement of splice site single nucleotide polymorphism (SNP) for growth habit differences in *Lablab purpureus* [25]. The role of *PHYA3* in photoperiod responsive flowering is already reported in *Glycine max* and *Phaseolus vulgaris*, and mutations in *E3/PHYA3* conferred photoperiod insensitive and early flowering [3, 26]. Lack of genome sequence data or any molecular information regarding *PHYA3* gene or any marker tightly linked to it in *Lablab purpureus* has limited the molecular characterization of photoperiod responsive flowering in this crop. Looking to possible role of *PHYA3* in photoperiod responsive flowering and lack of genome sequence information, the present work is focused on characterization of this gene in *Lablab purpureus* using candidate gene approach and phylogenetic analysis of legume phytochromes.

## Materials and methods

### Primer designing

The *GmPHYA3* sequence from *Glycine max* [3] was obtained in FASTA format from NCBI (National Centre for Biotechnology Information) GenBank database (https://www.ncbi.nlm.nih.gov/genbank/). *GmPHYA3* is 9.2kb longer, exon 1 was divided into three frames and primers for each frame were designed using web-based Primer BLAST (Basic Local Alignment Search Tool) from NCBI (Table 1). These primers were utilized for amplification of *PHYA3* in *Lablab purpureus* cv GNIB-21 which is determinate and photoperiod insensitive.

**Table 1:** Primers used for partial amplification *LprPHYA3* locus

Frame	Primer	Sequence	Amplicon length (bp)
Frame-1	93F	5′TGCATCAGATAACAGTGGAAGA3′	951
	94R	5′TTGTAGGATTGCAGGGCTCC3′	
Frame-2	115F	5′ATTTTGAGCCGGTCAAGCCT3′	987
	116R	5′CAGCTGCCATTCCACATGC3′	
Frame-3	133F	5′TTGTCTGATGCAGGCTTCCC3′	964
	134R	5′CCAAGCTGATGGGACCAGAA3′	

F and R represent forward and reverse primers, respectively

### Amplification and sequencing

Genomic DNA was isolated from young fresh trifoliate leaves of GNIB-21 using CTAB method [27]. The DNA quality and quantity were assessed using gel electrophoresis and spectrophotometer (Nanodrop 2000, Thermo Fisher, USA). Both the stock and diluted DNA preparations were stored at −50°C until use. The target frames were amplified in GNIB-21 through Polymerase Chain Reaction (PCR) with *Taq* DNA polymerase (TaKaRa, Clontech, Japan). PCR mixture prepared in 200 μl contained approximately 100 ng genomic DNA, 200 μM of dNTPs, 10 pmol of forward and reverse primers, standard *Taq* buffer (Mg^2+^ plus) and 1 unit of *Taq* DNA polymerase in total volume of 25 μl reaction. The PCR cycle involved initial denaturation of 95°C for 7 min followed by 35 cycles of 94°C (30 s), 51–55°C (45 s) and 72°C (1 min) and a final extension of 10 min at 72°C. Amplicons giving the single discrete band when resolved on 1.5% agarose gel electrophoresis with the expected product size were purified using column purification with SLS PCR Clean-up Kit (Saffron Life science, Surat, India). Sanger sequencing reaction of purified PCR amplicon was carried out with specific primers using BDT v3.1 Cycle sequencing kit on ABI 3730xl Genetic Analyzer (Applied Biosystems). Bidirectional sequence data obtained from each amplicon were processed using BioEdit [28]. The bidirectional sequence information obtained were processed by merging the sequences from three frames and overlapping sequences were identified in both directions to construct consensus sequence.

### Sequence retrieval, alignment and phylogenetic analysis

The processed *LprPHYA3* sequence was used as query for BLASTn at https://blast.ncbi.nlm.nih.gov/Blast.cgi for finding homologous sequences with reference to NCBI (National Center for Biotechnology Information) nucleotide database [29]. Sequences showing matches with *LprPHYA3* from Fabaceae family were retrieved from NCBI nucleotide database. MEGA X (Molecular Evolution Genetics Analysis) software [30] was used to align *PHYA* nucleotide sequences of 16 species from Fabaceae family in addition to *Arabidopsis thaliana* using the CLUSTAL W alignment algorithm [31]. All the alignment settings were employed at default values; 2160 and 2051 positions with and without gaps were obtained, respectively. The nucleotide substitutions selected with complete deletion of gaps or missing data were used to analyse sequences. The phylogenetic tree was inferred using Maximum Likelihood method based on Tamura-Nei Model [32]. The initial tree was inferred with default setting using Neighbour Joining method and Nearest-Neighbour Interchange was used as ML heuristic search method. The reconstructions of phylogenetic trees were conducted using Maximum Likelihood Method. Bootstraps with 1000 replicates for Poisson correction model were performed to assess node support [33]. The best-scoring ML tree was searched simultaneously to represent the evolutionary history of the 20 specimens tested.

### Prediction of conserved motifs

The detection of conserved motifs in DNA sequence of phytochrome gene was performed using online tool MEME (Multiple Em for Motif Elicitation) (https://meme-suite.org/meme/) [34]. This online web-based analysis was performed with minimum and maximum motif width of 6 and 12 residues, respectively, which were used to identify probable motifs, keeping the rest of the parameters at default. The MEME output in HTML showed the motifs as local multiple alignments of the input sequences, as well as in several other formats. MEME HTML output were allowed one or all of the motifs to be forwarded for additional investigation. The results of the MEME analysis were applied to TOMTOM (http://memesuite.org/tools/tomtom), which is the online software performing comparison of given motifs with available databases. The output generated from TOMTOM include sequence-logo graphics on behalf of the alignment of two motifs with p and q value (a measure of false discovery rate) of the match [35].

### Exon prediction, protein prediction and functional analysis

The identified partial sequence of *LprPHYA3* was subjected to exon prediction using Eukaryotic GeneMark.hmm version 3.54 [36]. The nucleotide sequence of exon-1 obtained in such a way was translated to protein sequence using standard codon table. This amino acid sequence obtained was further compared with *GmPHYA3* sequence. The protein sequence of exon 1 from *Lablab purpureus* GNIB-21 was used to perform BLASTp in NCBI GenBank database and the sequences showing homology were further used to create multiple alignment using the CLUSTAL W algorithm [29, 31]. Phylogenetic analysis with amino acid sequence of *PHYA* in 21 different plant species was also performed using JTT model in MEGA X [30, 37]. The online web-based functional analysis tool SMART-EMBL (Simple Modular Architecture Research Tool) (http://smart.embl-heidelberg.de/) Version 9 was used for predicting domains in both *GmPHYA3* and *LprPHYA3* protein sequences [38].

## Results

### *LprPHYA3* characterization and phylogenetic analysis

In the present study, *Lablab purpureus cv* GNIB-21, which is determinate and photo-insensitive, was used for characterizing *PHYA3* gene. The sequencing data after processing and analysis revealed the successful characterization of the exon-1 of *PHYA3* gene in Indian bean (*LprPHYA3*- *Lablab purpureus PHYA3*) for the first time in the world. BLASTn analysis of nucleotide sequence *LprPHYA3* indicated highest identity with *Vigna unguiculata* (95.76%) followed by *Vigna angularis* (95.67%) and *Glycine max* (90.90%), all with *E*-value close to zero.

**Fig. 1:**
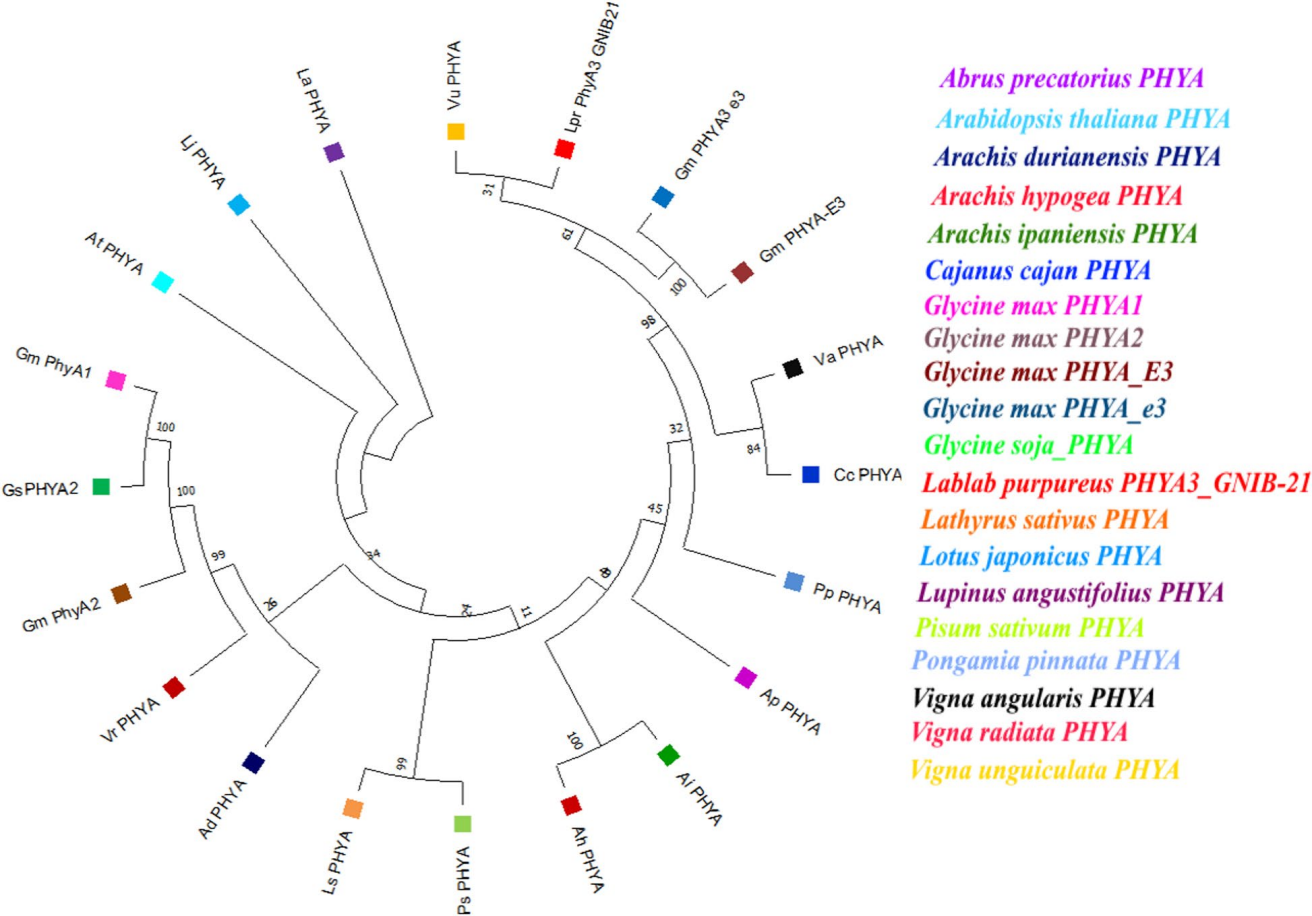
Phylogenetic tree of the Phytochrome A gene inferred by the Maximum Likelihood algorithm corresponding to the lowest value of the Log Likelihood function. The percentages of bootstrap support (1000 replicates) are shown near corresponding nodes. Names of genes are included for each taxon. *Arabidopsis thaliana*, *Lotus japonicus* and *Lupinus angustifolius* are three outgroups

Phylogenetic tree depicts formation of different clades on the basis of evolutionary changes between the sequences. *LprPHYA3* nucleotide sequence was compared with the nucleotide sequences of phytochrome genes of other plant species, most of which, belonged to the Fabaceae family. Phylogenetic analysis showed that this phytochrome A gene evolved from common ancestry root but diverged into different clades during the course of evolution (Fig. 1). The *PHYA* sequences from *Lablab purpureus*, *Vigna unguiculata* and *Glycine max* form independent clade and are closest to *Vigna angularis* and *Cajanus cajan*. This indicates that *LprPHYA3* has maximum closeness to *Vigna unguiculata*, *Glycine max*, *Cajanus cajan* and *Vigna angularis PHYAs* suggesting the evolutionary closeness of the gene in these species. The three outgroups depicted in the tree are *Lupinus angustifolius*, *Lotus japonicus* and *Arabidopsis thaliana*.

**Fig. 2:**
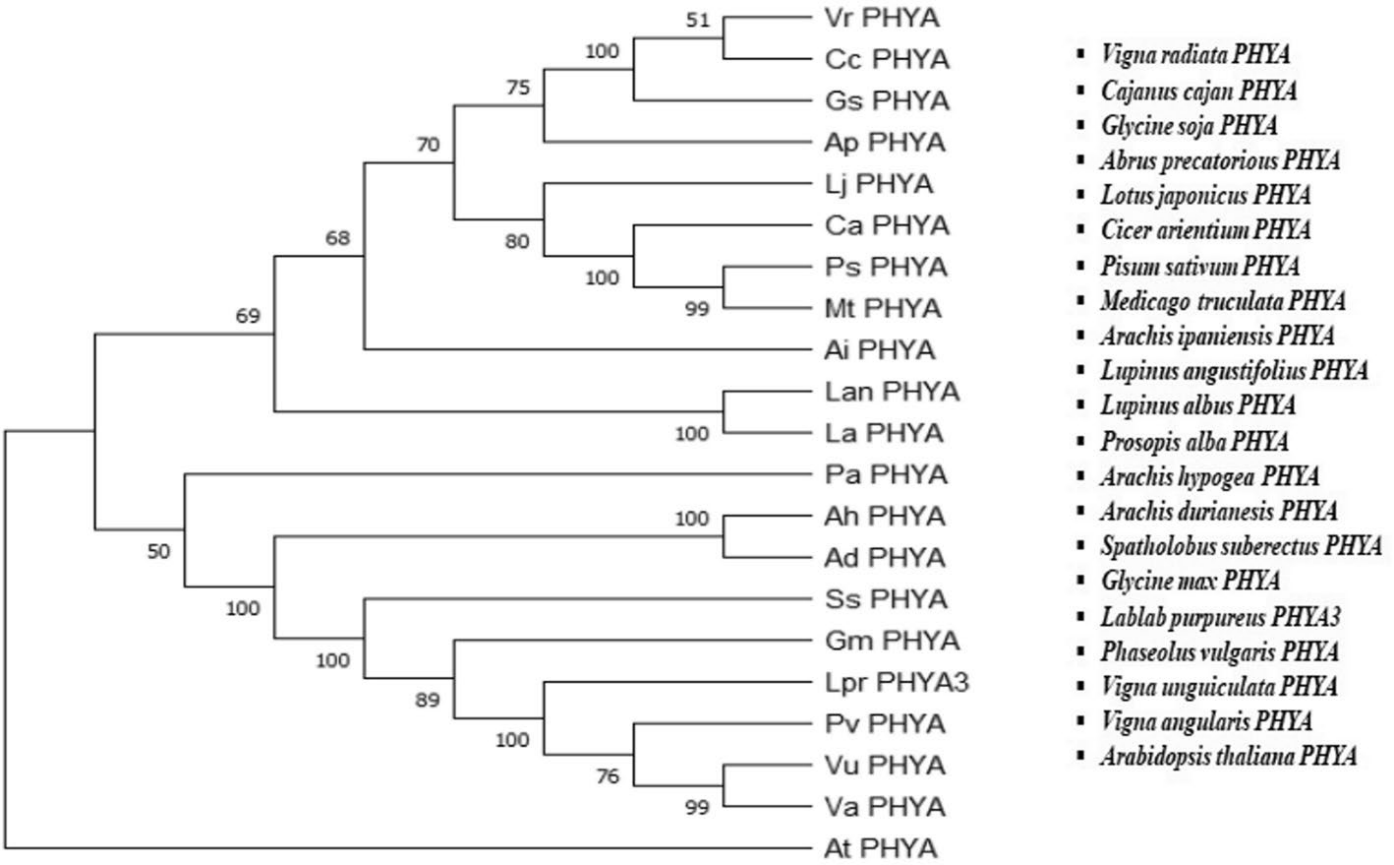
Phylogenetic tree of amino acid sequences of phytochrome A by Maximum Likelihood algorithm showing evolutionary lineage using MEGA X software. The bootstrap consensus tree is inferred from 1000 replicates; with the confidence values shown next to the branches, *Arabidopsis thaliana* is an outgroup

Realizing the importance of both DNA and protein sequence alignment in phylogenies, the protein sequence predicted from the exon 1 of *LprPHYA3* gene was also studied along with the other sequences to delineate the amino acid changes in the sequence during the course of evolution. Alignment of the amino acid sequences from *PHYA* homologs was done by CLUSTAL W alignment algorithm and phylogenetic tree for protein sequences was constructed (Fig. 2). The protein alignment also depicts the most probable evolution of *LprPHYA3* from common ancestral *PHYA* gene of *Glycine max*, *Phaseolus vulgaris*, *Vigna unguiculata* and *Vigna angularis*. This tree depicts *Arabidopsis thaliana* as an outgroup.

### Determination of common motifs in *PHYA* sequence

**Fig. 3:**
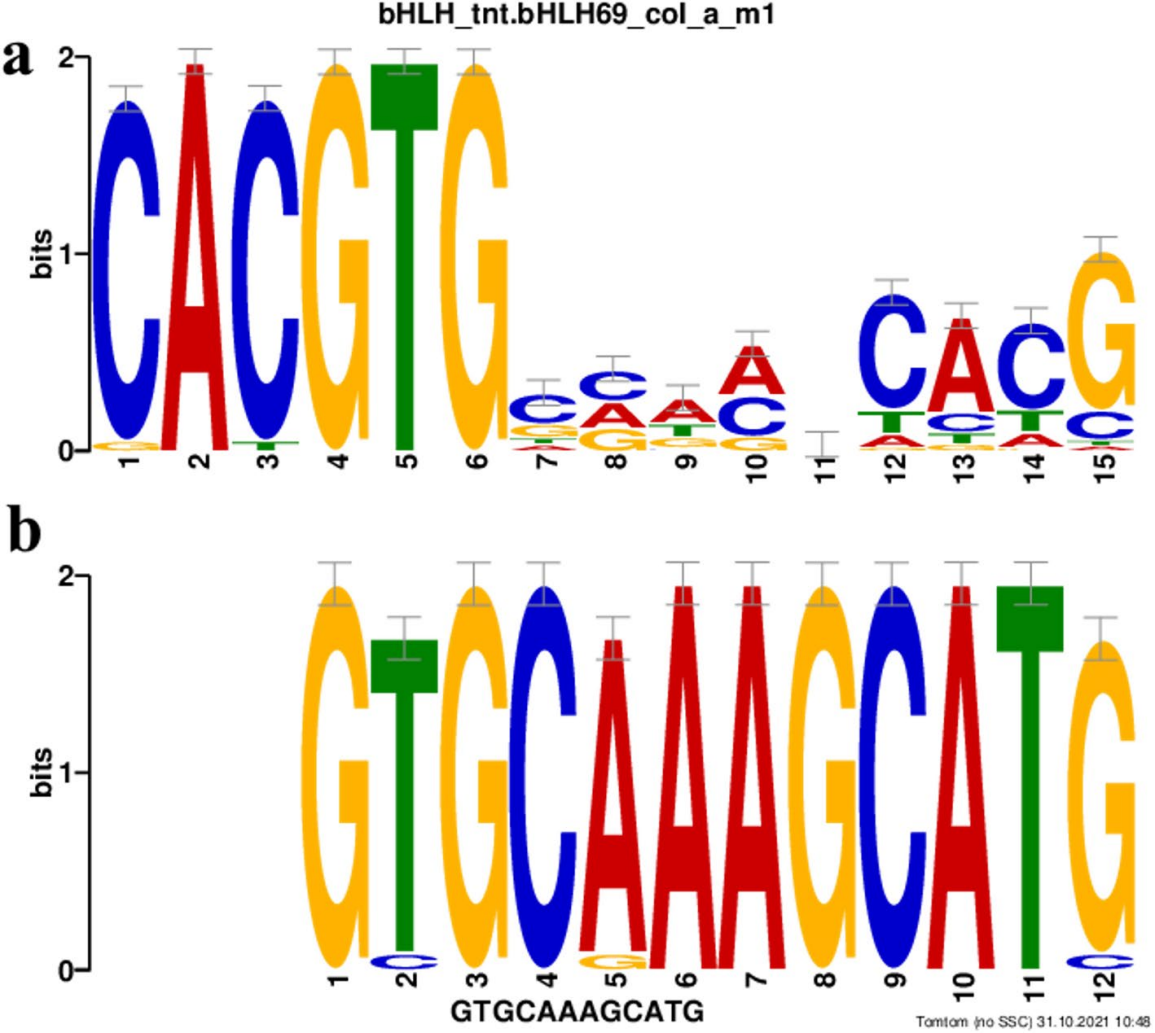
The sequence-logo comparison of conserved motifs between **a ***Arabidopsis thaliana* bHLH69 and **b** the motif found in putative phytochrome A genes

Identification of conserved motifs in the *PHY A* was performed by comparing DNA sequences from these 20 *PHY A* sequences from different species. The analysis resulted in three conserved sites present in pool of studied sequence. The best result was a 12-nt-long motif GTGCAAAGCATG which was found in all 20 analysed sequence (Table 2). The *E*-value for best match discussed here was 3.4×10^−45^. Subsequently, the motif was compared using TOMTOM with a database of *Arabidopsis thaliana* DAP motifs which resulted in identification of bHLH69 basic helix-loop-helix motif containing transcription factor (*p* value 1.16×10^−4^ and *E* value 1.01×10^−1^) (Fig. 3).

**Table 2:** Predicted conserved motifs in 20 sequences of Phytochrome A gene

Sequence	Length	Start	***P***-value	Sites		
*Ad_PHYA*	2054	1598	5.50×10^−8^	AGATGGGGTG	GTGCAAAGCATG	ACCCTGGTGA
*Ah_PHYA*	2057	884	5.50×10^−8^	GTTGATTGTC	GTGCAAAGCATG	TGAAGGTGCT
*Ai_PHYA*	2057	884	5.50×10^−8^	GTTGATTGTC	GTGCAAAGCATG	TGAAGGTGCT
*Ap_PHYA*	2073	896	5.50×10^−8^	GTTGATTGTC	GTGCAAAGCATG	TGAAAGTCCT
*At_PHYA*	2057	1601	1.42×10^−7^	AGATGGGGAG	GTGCAAAGCATG	ATCCAGATGA
*Cc_PHYA*	2076	896	5.50×10^−8^	GTTGATTGTC	GTGCAAAGCATG	TTAAGGTGCT
*Gm_PHYA1*	2077	1589	5.50×10^−8^	CGATGGGGTG	GTGCAAAGCATG	AGGCTGGAGA
*Gm_PHYA2*	2072	1589	5.50×10^−8^	CGATGGGGTG	GTGCAAAGCATG	AGGCTGGAGA
*Gm_PHYA3_E3*	2077	899	5.50×10^−8^	GTTGATTGTT	GTGCAAAGCATG	TGAATGTGCT
*Gm_PHYA3_e3*	2077	899	5.50×10^−8^	GTTGATTGTT	GTGCAAAGCATG	TGAATGTGCT
*Gs_PHYA2*	2056	1595	5.50×10^−8^	CGATGGGGTG	GTGCAAAGCATG	AAGCTGGAGA
*La_PHYA*	2063	454	5.50×10^−8^	CGCTGGGGTG	GTGCAAAGCATG	AACCTGGTGA
*Lj_PHYA*	2059	1598	5.50×10^−8^	CGATGGGGTG	GTGCAAAGCATG	AACCTGGAGA
*Lpr_PHYA3*	2077	902	5.50×10^−8^	GTTGATTGTC	GTGCAAAGCATG	TGAAGGTGCT
*Ls_PHYA*	2059	1598	5.50×10^−8^	CGATGGGGTG	GTGCAAAGCATG	AACCGGGCGA
*Pp_PHYA*	474	26	3.18×10^−7^	GTTGATTGTC	GCGCAAAGCATC	TGAAGGTTCT
*Ps_PHYA*	2056	1598	5.50×10^−8^	CGATGGGGTG	GTGCAAAGCATG	AACCGGGCGA
*Va_PHYA*	2079	902	5.50×10^−8^	GTTGATTGTC	GTGCAAAGCATG	TGAAGGTGCT
*Vr_PHYA*	2051	1595	5.50×10^−8^	CGATGGGGTG	GTGCAAAGCATG	AAGCTGGAGA
*Vu_PHYA*	2079	902	5.50×10^−8^	GTTGATTGTC	GTGCAAAGCATG	TGAAGGTTCT

The column representing sequences are phytochrome A (*PHY A*) nucleotide sequences from 20 species. *Ad_PHYA Arachis durianesis*, *Ah_PHYA Arachis hypogea*, *Ai_PHYA Arachis ipaniensis*, *Ap_PHYA Abrus precatorious*, *At_PHYA Arabidopsis thaliana*, *Cc_PHYA Cajanus cajan*, *Gm_PHYA1*, *Gm_PHYA2*, *Gm_PHYA3_E3*, *Gm_PHYA3_e3 Glycine max PHY A* homologs, *Gs_PHYA2 Glycine soja*, *La_PHYA Lupinus angustifolius*, *Lj_PHYA Lotus japonicus*, *Lpr_PHYA3 Lablab purpureus*, *Ls_PHYA Lathyrus sativus*, *Pp_PHYA Pongamia pinnata*, *Ps_PHYA Pisum sativum*, *Va_PHYA Vigna angularis*, *Vr_PHYA Vigna radiata*, *Vu_PHYA Vigna unguiculata*. The following columns present length of DNA sequence, start position, random letters probability and the sequence of the conserved motif

### Domain analysis of *GmPHYA3* and *LprPHYA3*

**Fig. 4:**
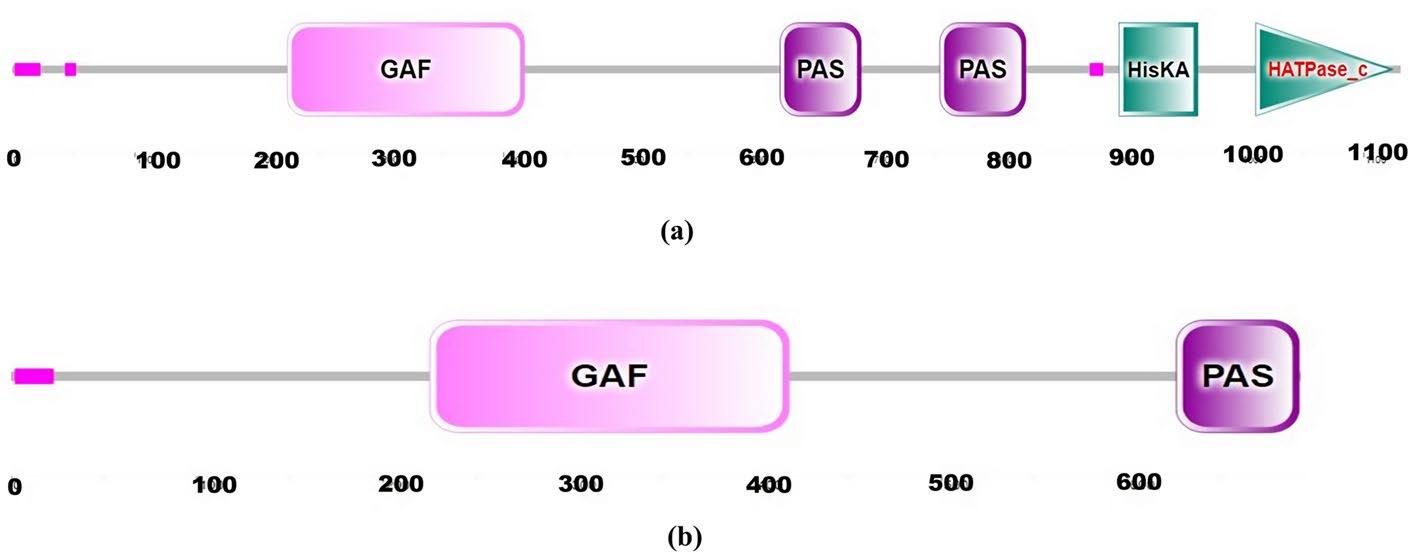
**a** Domain structure analysis of amino-acid sequence for *GmPHYA3*. **b** Domain structure analysis of exon-1 of *LprPHYA3.* The numbers represent position of amino acid. The domains for *PHYA3* protein included GAF (cGMP-specific phosphodiesterase–adenylyl cyclase–FhlA), PAS (period–ARNT–single-minded) and Histidine kinase-related domains (HKRD) *viz*., HisKA and HATPase_c

Domain analysis of *GmPHYA3* sequence retrieved from NCBI GenBank database after performing BLASTp search with *LprPHYA3* that encodes a protein with 1130 amino acids was carried out using EMBL-SMART platform. This protein displayed normal features of Phytochrome A with five domains *viz.*, GAF (cGMP-specific phosphodiesterase–adenylyl cyclase–FhlA), two PAS (period–ARNT–single-minded) domains and two Histidine kinase-related domains (HKRD) *viz.*, HisKA and HATPase_c as depicted in Fig 4a. The amino acid sequence of *LprPHYA3* showed two domains, i.e. GAF sand PAS (Fig 4b). This finding is in congruity with the domain analysis from *GmPHYA3*.

## Discussion

**Fig. 5:**
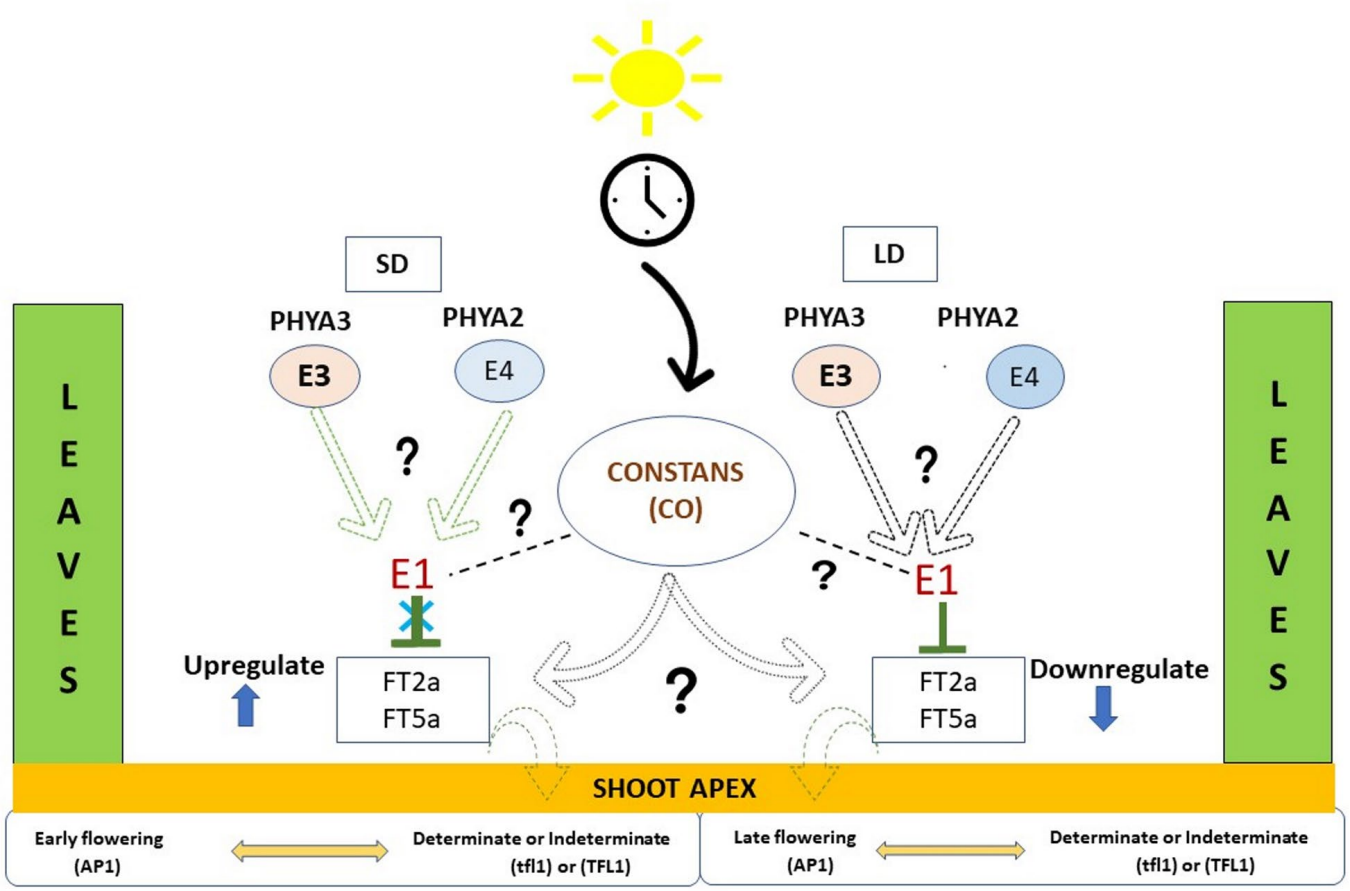
A theoretical model for photoperiod responsive flowering and growth habit mediating plant architecture in response to short day (SD) and long day (LD) conditions based on genes reported in *Lablab purpureus*, *Glycine max* and related legumes. Light arrows in SD indicate repressed *E3* and *E4* during short days. Question marks indicate the unknown genes involved in mediating the process. Genes *E4*, *E1*, *FT2a* and *FT5a* are yet to be identified in *Lablab purpureus*. *PHYA3* and *PHYA2* are homologs of phytochrome A. *E1*, *E3* and *E4* are maturity loci governing photoperiodic regulation in *Glycine max*. *FT2a* and *FT5a* are *Flowering locus T* homologs reported as florigens in many legumes and induce flowering

The deciphering of putative molecular pathways in legumes involving different phytochrome genes and their involvement in governing flowering time will pave way for future research. Various genetic models for this regulatory framework of photoperiod-based flowering with different known loci is available for *Glycine max*. It is studied in depth for photoperiod control of flowering and different *E* loci have been reported, out of which, *E3* (*GmPHYA3*) is considered a strong candidate for *FT3* and mutations in *GmPHYA3* results in early flowering [3]. Different mutations of *E3* have been reported in *Glycine max* and *Phaseolus vulgaris* that confer significantly early flowering and photoperiod insensitivity [3, 26]. Additionally, naturally occurring *E3* mutants depicted a large insertion in fourth intron and one SNP for non-synonymous amino acid substitution in third exon. Naturally occurring *e3* allele carries a large deletion spanning exon 4, whereas an induced mutant *e3* allele has sustained a 40-bp deletion and frameshift in the middle of exon 1 [3]. Flowering time is a very important trait governing several other correlated functions and molecular identification of flowering networks with these *E* loci can be used for more efficient breeding strategies [39]. These pathways have been more or less conserved in related legume species involving numerous loci with known or unknown functions. On the basis of available information on related genes and pathway undermining photoperiodic flowering in legumes, a theoretical model for photoperiod dependent flowering pathway is proposed (Fig. 5). In *Lablab purpureus*, photoperiodic response of flowering may also be governed by circadian clock as it is reported in *Glycine max*. CONSTANS (CO) is a circadian-regulated gene and acts as prime regulator of this pathway. It activates the expression of florigen gene *FT* by coordinating light and clock input to leaves. The different genes, their responses to photoperiod and their putative roles mediating this process have been proposed. It is speculated that under SD condition (low R:FR), *E3* and *E4* are repressed due to lack of exorbitant light condition and thus *E1* is also suppressed which thus has no effect on *FT2a* and *FT5a* genes leading to early flowering phenotype. Under enriched light condition, i.e. LD condition *PHYA3* (*E3*) and *PHYA2* (*E4)* are expressed activating the expression of *E1* which eventually leads to *FT2a*/*FT5a* downregulation, flowering repression, indeterminate growth (if *TFL1* is present) and delayed maturity. The presence of *TFL1* and/or *tfl1* convoys indeterminate and determinate growth habit, respectively [1, 15]. Dominant *TFL* allele suppresses development of floral architecture at shoot apex in indeterminate types; racemes emerge from axillary bud only upon short day conditions where *FT*s, expressed due to favourable photoperiod condition, might be able to nullify the effect of *TFL* in competitive manner. Determinate growth habit results from non-functional allele (*tfl*) of *TFL* which is unable to suppress flowering in shoot apex and results into determinate growth habit and photoperiod insensitive flowering. This is unveiled by the presence of splice site SNP at third exon in *Lprtfl* which renders non-functional protein and is responsible for determinate growth habit and photo-insensitive flowering in *Lablab purpureus* cv GNIB-21 [25]. A non-synonymous SNP in exon 4 of *TFL1* locus in cowpea also resulted in determinate growth habit [40]. However, photoperiod responsive expression of growth habit cannot be ignored [41].

Legumes have always been of persistent interest due to vast genetic variation for photoperiod responses mediating crucial traits like growth habit, flowering and maturity. A summary of different phytochrome *A* genes in some legumes along with their putative role is represented (Table 3).

**Table 3:** Summary of identified *Phytochrome A* genes in different crop legumes

Gene	Legume species	Function/role	Reference
*PHYA3 E3*	*Glycine max*	R/FR photoreceptor; represses FT; delays flowering in LD	[3]
*PHYA3 e3*	*Glycine max*	Promotes flowering in LD	[42]
*PHYA2 E4*	*Glycine max*	R/FR photoreceptor; represses FT; delays flowering in LD	[17]
*PHYA1*	*Glycine max*	Unknown	[17]
*PHYA2 E4*	*Glycine soja*	Inhibits flowering	[42]
*PHYA*	*Pisum sativum*	R/FR photoreceptor	[43]
*PHYA3*	*Phaseolus vulgaris*	R/FR photoreceptor; represses FT; delays flowering in LD	[26]
*PHYA and PHYB*	*Arachis hypogea*	Gynophore enlargement	[44]
*PHYA*	*Vigna radiata*	Control FT	[45]

Seeking the importance of *GmPHYA3* (or *E3*) in photoperiod adaptation of short-day legumes like *Glycine max* and *Phaseolus vulgaris*, it becomes quite important to understand its role in *Lablab purpureus* which is also a short-day crop. Due to the lack of genome sequence database, there is very scanty information available about *Lablab purpureus* at molecular level. The present study was successful in characterizing exon-1 in *LprPHYA3* by utilizing *GmPHYA3* sequence as reference. Comparative gene mapping has been quite useful in deducing genomic structure from related plant species due to conservation of genetic content, gene order and function [25, 46]. This marks the significance of phylogenetic analysis to depict evolutionary closeness of different plant species. DNA-based phylogenies have been dominated in recent years mainly because it yields more phylogenetic information than protein. Since non-synonymous mutations do not affect amino acid sequence but do alter nucleotide sequences of a pair of homologous genes. Both DNA- and amino acid sequence-based phylogenies have been conducted in present study for validation of results obtained by phylogenetic analysis. DNA sequence phylogenies provide opportunity to the researcher for examination of both coding and non-coding regions of gene. However, the use of protein sequences in establishing evolutionary relationships cannot be ignored as amino acid sequences are more conserved than DNA sequences. The degeneracy in genetic code and difference in codon usage in different species makes it less accepted. Protein sequence gives more sensitive sequence alignment as DNA has only 4 characters, while protein has 20. The translation of DNA to protein gives a higher signal-to-noise ratio and thus sharpens up the analysis making it better for phylogenetic studies [47]. *PHYA3* gene has most probably been evolved from common ancestral *PHYA* gene of these species, as depicted from the tree. Previous studies have also shown closeness of *Lablab purpureus* to *Vigna unguiculata* and their predictable evolution from *Glycine max* in Phaseoleae clade [48]. This study has delineated the conservation of *PHYA3* among the phaseoleae clade legumes indicating evolutionary closeness with *Vigna unguiculata*, *Vigna angularis* and *Glycine max* employing both DNA and protein sequences for phylogenetic analysis (Figs. 1 and 2). The evolutionary relationship of *Lablab purpureus* to *Glycine max*, *Vigna unguiculata* and *Phaseolus vulgaris* among the Phaseoleae clade has been reported earlier [48–50]. This will aid in isolating allelic variants of *PHYA3* from *Lablab purpureus* by utilizing model plants like *Glycine max* and *Phaseolus vulgaris*. Characterization of the full gene could pioneer the extensive photoperiodic flowering control mechanism in *Lablab purpureus*.

Phytochromes mediates light responses by interacting with multiple partners to modulate transcription of downstream target genes. The transcription factor (TF) containing basic helix-loop-helix (bHLH) motif interacts physically with red and far-red photoreceptor, phytochrome, called Phytochrome Interacting Factors (PIF) [51]. The present study was also successful in identifying bHLH69 as conserved motif for *PHYA* gene (Fig. 3 and Table 2). The TF with bHLH motif has already been known to be involved in regulating circadian rhythm in *Arabidopsis* [52, 53]. Presence of DNA binding motif (bHLH) in *PHYA* indicates that it might compete with PIFs for DNA binding to repress flowering [51]. The present study also deciphered the domains encoded by exon 1 of *LprPHYA3* (Fig. 4) which are in congruity with the *Glycine max PHYA3* [3]. This implies to the fact that exon 1 in *LprPHYA3* codes for GAF and PAS domains with chances of conserved functions. The plant phytochromes detect light *via* their amino-terminal photosensory module (PSM) comprising N-terminal extension (NTE), period–ARNT–single-minded (PAS), cGMP-specific phosphodiesterase–adenylyl cyclase–FhlA (GAF) and phytochrome-specific (PHY) domains with the help of a bilin chromophore. C-terminal output module (OPM) is shared by two PAS on the N-terminal side and a histidine kinase-related domain (HKRD) [54]. NTE is related to the stability of light-activated phytochromes and interacts with the part of GAF which binds to PɸB for lyase activity and reversible Pr/Pfr photo-transformation. PAS domain represents transducer domain that mediates light signal from input photosensory domain to output module. HKRD domain plays major roles in dimerization, nuclear import and localization [55]. *LprPHYA3*-Exon 1 encodes GAF and PAS domain of phytochrome genes which belongs to the photo-sensory module and is responsible for convertible Pr/Pfr transformation as well as light-signal transduction from this module to output module, respectively [56]. Characterization of full gene sequence of *PHYA3* in *Lablab purpureus* would unravel the different domains involved in downstreaming light-mediated response to the signaling pathway along with their putative roles.

## Conclusion

Partial characterization of *LprPHYA3* would facilitate allelic characterization in relation to photoperiod responsive flowering in *Lablab purpureus.* Phylogenetic analysis indicated that complete characterization of *LprPHYA3* would be possible utilizing sequence information from related legumes. The presence of conserved DNA binding motif (bHLH69) in *PHYA* gene indicated that it might repress flowering by competing for DNA binding with bHLH containing TFs. Domain analysis of protein-encoding *LprPHYA3* would unfold the signaling pathways and their interaction with different proteins from PEBP (Phosphatidyl ethanolamine-binding protein) family genes that would guide flowering response.

The continued progress in this direction would entice further questions to address in future like characterization and identification of allelic variants for *LprPHYA3* and their role in modulating photoperiod responsive flowering. Additionally, qPCR studies could also be undertaken for relative expression studies of *PHYA3* in LD and SD conditions. The role of *LprPHYA3* may be confirmed through genome editing by utilizing partial sequence reported in the present study. These efforts would accelerate the understanding of flowering time and growth habit regulation in *Lablab purpureus* in response to changed photoperiod.

## Supplementary Information

**Additional file 1.** Sequencing data.

## Data Availability

The dataset supporting the conclusions of this article are included within the article. The sequencing data have been submitted as supplementary material.

## Abbreviations

R/FR: Red/Far-Red
*PHY*: Phytochrome
FT: Flowering locus T
LD: Long day
SD: Short day
DT: Determinate
IDT: Indeterminate
PS: Photosensitive
PIS: Photo-insensitive
SNP: Single nucleotide polymorphism
NCBI: National Centre for Biotechnology Information
BLAST: Basic Local Alignment Search Tool
MEGA: Molecular Evolution Genetics Analysis
MEME: (Multiple Em for Motif Elicitation)
CTAB: Cetyl trimethyl ammonium bromide
PCR: Polymerase chain reaction
SMART: Simple Modular Architecture Research Tool
DNA: Deoxyribonucleic acid
PSM: Photosensory module
NTE: N-Phosphodiesterase–adenylyl cyclase–FhlA (GAF)
PHY: Phytochrome-specific
OPM: Output module
HKRD: Histidine kinase-related domain
PIF: Phytochrome-interacting factors
CO: Constans
AP1: Apetela 1
TF: Transcription factors
bHLH: Basic helix–loop–helix
PEBP: Phosphatidyl ethanolamine-binding protein
