# Supplementary material for: Phylogenetic analysis of phytochrome A gene from Lablab purpureus (L.) Sweet
Source: J Genet Eng Biotechnol. 2022 Jan 13;20:9. doi: 10.1186/s43141-021-00295-z (PMC8758814; doi:10.1186/s43141-021-00295-z)
Supplement: Supplementary file 1 — Additional file 1. Sequencing data. [file 43141_2021_295_MOESM1_ESM.docx]

**Supplementary Material**

1. Nucleotide sequence of Exon-1 of *LpPHYA3* in Indian Bean (*Lablab purpureus*)

>Lp_PhyA3_GNIB21 (NCBI GeneBank accession number: OL757533)

ATGTCCTCCTCAAGGCCTAGCCCATCATCGAGCAATAATTCTGGAAGATCAAGATCATCAAGACGCAGTGCTAGGGTTCTTGCTCAGACAACTTTAGATGCAAAACTGCATGCAACTTTTGAGGAATCAGGTAGTTCTTTTGACTACTCCAATTCAGTGAGATTGTCTCCTGGCACAGGTACTGCCAGTGGTGATCATCAATCAAAGTCTGATGGAGTAACAACTGCTTACCTCCATCAGTTACAGAAAAGCAAGCTTATCCAACCATTTGGGTGCTTGCTGGCCTTAGATGAGAAAACATGTAAGGTCATTGCTTACAGTGAGAATGCACCTGAAATGCTCACCATGGTTAGCCATGCTGTCCCCAGTGTTGGTGACCACCCTGCTCTTGGCATTGGCACTGACATAAGAACAATTTTCACTGCCCCGAGTTCTACGTCTATTCAGAAGGCACTAGGATTTGGGGAGGTTTCACTTCTTAACCCCATTCTAGTTCATTGCAAGTCCTCTGGGAAGCCCTTTTATGCAATTATCCACCGTGTTACCGGTAGTGTGATAATTGATTTTGAGCCGGTTAAGCCTCATGAAGTTCCCATGACTGCCGCAGGTGCCCTGCAATCCTATAAGTTTGCAGCAAAAGCAATAACAAGATTGCAATCCTTGCCTGGTGGGAGCATGCAGACTCTGTGTGACACAATGGTTAGAGAGGTTTTTGAACTCACAGGTTATGATAGAGTGATGGCTTATAAATTTCATGAGGATGATCACGGGGAAGTGATTGCTGAAGTGAAAAGGCCAGGCCTAGAGCCATATCTGGGGTTGCACTACCCAGCAACTGATATTCCTCAGGCTACACGTTTTTTGTTTATGAAGAACAAGGTGCGCATGATAGTTGATTGTCGTGCAAAGCATGTGAAGGTGCTTCAAGACAAAAAAATTCCATTTGAATTAACTTTGTGTGGATCAACCTTGAGGGCTGCTCATAGTTGTCACTTGCAGTACATGGAAAACATGAATTCTAGTGCTTCCTTGGTTATGGCGGTTGTGGTCAATGACAATGATGAAGATGGGGATAGTTCTGATGCTGTTCAGCCACAGAAGAGAAAGAGACTATGGGGTTTAGTAGTTTGCCATCACTCCACTCCCAGGTTTGTTCCTTTCCCTCTCAGGTATGCTTGTGAATTCCTGGCTCAGGTATTTGCCATCCATGTGAACAAAGAACTAGAGATAGAGTATCAGATTGTTGAGAAGAATATCCTCAGGACTCAAACACTGTTGTGTGATATGCTGATGCGAGATGCACCCCTAGGTATTGTATCACAGAGTCCAAATATAATGGATCTTGTTAAGTGCGATGGTGCAGCACTGTTGTATAAAAACAAGGTATGGAGATTAGGGGTAACACCAAGTGAATCCCAGATAAGAGAGATAGCTTTGTGGCTCTCTGAATGTCACAGGGATTCCACAGGTTTGAGTACAGATATTTTGTCTGATGCAGGCTTCCCAGGGGCTGCTACTCTTGGAGATATAGCTTGTGGAATGGCAGCTGTCAGAATATCTTCCAAAGATATAGTTTTCTGGTTTCGATCTCACACAGCCGCAGAAATTCGATGGGGTGGTGCAAAGCATGAGCCTGGTGAAAGGGACGATGGTAGGAGGATGCATCCAAGATCTTCATTCAAGGCTTTCCTTGAAGTTGTGAAGACACGGAGTTTGCCCTGGAAGGACTATGAAATGGATGTCATTCATTCGTTGCAGCTAATACTGAGAAATGCATTCAAAGACACAGAGAGTATGGAGATAAGCACATATGCTATCAATACAAGATTAGGTGATTTGAAAATTGAAGGGATGCAAGAACTGGAAGCAGTGACTAGTGAGATGGTAAGATTAATTGAAACAGCAACAGTGCCAATTTTGGCTGTTGATGTTAATGGGATGGTCAATGGATGGAATACAAAAATTGCTGAGTTGACATGTCTTCCAGTTGAGCAAGCTATTGGGAAGAATTTACTCACTCTTGTTGAAGATTTTTCAGTAGATAGAGTCAAGAAGATGTTGGACATGGCACTGCAGG

1. Amino acid sequence of Exon-1 of *LpPHYA3* in Indian Bean (*Lablab purpureus*)

>LpPhyA3_GNIB21

MSSSRPSPSSSNNSGRSRSSRRSARVLAQTTLDAKLHATFEESGSSFDYSNSVRLSPGTGTASGDHQSKSDGVTTAYLHQLQKSKLIQPFGCLLALDEKTCKVIAYSENAPEMLTMVSHAVPSVGDHPALGIGTDIRTIFTAPSSTSIQKALGFGEVSLLNPILVHCKSSGKPFYAIIHRVTGSVIIDFEPVKPHEVPMTAAGALQSYKFAAKAITRLQSLPGGSMQTLCDTMVREVFELTGYDRVMAYKFHEDDHGEVIAEVKRPGLEPYLGLHYPATDIPQATRFLFMKNKVRMIVDCRAKHVKVLQDKKIPFELTLCGSTLRAAHSCHLQYMENMNSSASLVMAVVVNDNDEDGDSSDAVQPQKRKRLWGLVVCHHSTPRFVPFPLRYACEFLAQVFAIHVNKELEIEYQIVEKNILRTQTLLCDMLMRDAPLGIVSQSPNIMDLVKCDGAALLYKNKVWRLGVTPSESQIREIALWLSECHRDSTGLSTDILSDAGFPGAATLGDIACGMAAVRISSKDIVFWFRSHTAAEIRWGGAKHEPGERDDGRRMHPRSSFKAFLEVVKTRSLPWKDYEMDVIHSLQLILRNAFKDTESMEISTYAINTRLGDLKIEGMQELEAVTSEMVRLIETATVPILAVDVNGMVNGWNTKIAELTCLPVEQAIGKNLLTLVEDFSVDRVKKMLDMALQ

3. Nucleotide sequences retrieved from NCBI-BLAST for phylogenetic analysis

>XM_027497677.1 PREDICTED: Abrus precatorius phytochrome A-like

TGGCATGGCAGTGTCTTAGTTGTAAGTTCTAACTGCTGAGTTATGTCTGAGTCTTGAGCTTGGAGGGGTTCCCACTTTCCACTTAGCTGTGTCTTCACTCTTCAATTCAAAGGTCGGTGATTAAGATTTTCTGCTTCATCACAACCTGATATATATATATATATATATAGATGCTGCTGTAACAGTAAGTAGCTAGTTGGGATTATTATTCCCTAGCTGCTTCTGCTTCTGCTTCTACTCAAACCTTTTCTCTACTCATTGGTTGGAATCGGAATTTTTCTTGCAAGTGAGTAAGGATGATGTCATTTGACTAAAAGTCTATAGTAGATTTGCACCAGTGGTAGAATAGATTCCGTTCTTCCTTGAAAGAGATTAACTTAGTAATCTTATTCTTAAGATTCCAAGTGCAGTTGAAGTGACAATGTCCTCCTCAAGGCGTAGCCAATCATCCAGCAACAATTCAGGCAGATCATCAAGACACAATGCTAGGATAATTGCTCAAACCACTGTAGATGCAAAACTCCATGCAACTTTTGAGGAGTCTGGTAGTTCTTTTGACTACTCCAACTCAGTGAGGTTTTCTCCTGGCTCAGGCACAGCTAGTGGAGAACATCAATCAATGTCTGATAGAGTAACAACTGCATATCTCCATCAGTTTCAGAAAAGCAAGCTTATTCAACCTTTTGGGTGCTTGCTAGCTTTAGATGAGAAAACATGCAAAGTCATTGCATACAGTGAGAATGCACCTGAAATGCTGACTATGGTTAGTCATGCTGTTCCCAGTGTTGGTGATCACCCTGCTCTTGGCATTGGTACTGACGTAAGAACAATTTTCACTGCCCCAAGTGCTTCTGCATTTCAGAAGGCACTAGGATTTGGAGAGGTTTCACTTCTTAACCCAATTCTAGTTCATTGCAAAACCTCCGGGAAGCCCTTTTATGCAATTATCCATCGTGTTACCGGTGGCTTGATCATTGATTTTGAGCCAGTCAAGCCTCATGAAGTTCCCATGACTGCAGCAGGTGCCTTGCAGTCCTACAAGCTTGCAGCAAAAGCAATAACCAGGTTGCAATCTTTGCCTAGTGGGAGCATGGAAACACTATGTGACACAACGGTTCAAGAAGTGTTTGAACTCACAGGTTATGACAGGGTGATGGCTTATAAGTTTCATGAGGATGATCATGGAGAAGTGATTGCAGAGGTAAAAAAGCCAGGCCTAGAGCCGTATCTGGGTTTGCACTATCCAGCCACTGATATTCCTCAGGCTACACGCTTTTTGTTTATGAAGAACAAGGTTCGTATGATAGTTGATTGTCGTGCAAAGCATGTGAAAGTCCTTCAAGACAAAAACATTCCATTTGATTTAACTTTGTGTGGATCCACCTTAAGGGCTCCTCATAGTTGCCATTTGCAATACATGGAGAACATGAATTCTAGTGCTTCCTTGGTTATGGCAGTTGTAGTCAATGACAATGATGAAGATGGAGATAGCTCTGATGCTGTTCAGCCACAAAAGAGAAAGAGGCTTTGGGGTCTAGTAGTTTGCCATCACACTACTCCCAGGTTTGTTCCTTTTCCTCTTAGGTATGCTTGTGAATTCTTGGCTCAAGTATTTGCCATCCATGTGAACAAAGAACTAGAAATAGAGTATCAGATTGTTGAGAAGAATATCCTGCGCACTCAAACACTCTTGTGTGATATGTTGATGCGAGATGCACCCCTTGGCATTGTATCACAAAGGCCTAATATAATGGATCTTGTGAAATGTGATGGAGCAGCCCTGTTGTATAAGAACAAGGTATGGAAATTAGGGGTAACGCCAAGCGAATCACAATTCGAGAGATAGCTTTATGGCTCTCTGAGTGCCATAGGGATTCCACAGGTTTGAGTACAGATAGCTTGTCAGATGCAGGGTTCCCAGGGGCTGCTGCTCTTGGTGATATTGTATGTGGAATGGCAGCTGTGAGAATTGCAACCAAAGACATGGTTTTCTGGTTTCGGTCTCACACTGCTGCTGAAATCAGATGGGGTGGTGCAAAGCATGAACCTGGTGATAGGGATGATGGTAGGAGGATGCACCCAAGATCATCCTTCAAGGCTTTCCTTGAAGTTGTTAAGACAAGGAGCTTACCGTGGAAGGACTATGAAATGGATGCTATCCACTCATTGCAGCTAATACTGAGAAATGCATTCAAAGATACCGAGAGTATGGATATAAGCACGTATGCTATCAATACAAGACTAAACGATTTGAAGATTGAAGGGATGCAAGAACTGGAAGCAGTGACAAGTGAGATGGTAAGGTTGATTGAAACAGCAACGGTGCCAATTTTGGCTGTTGATGTTCATGGTATGGTCAATGGATGGAATACAAAAATTGCTGAGTTGACAGGTCTTCCAGTTGAAGAAGCTATTGGGAAGCATTTACTCACACTTGTTGAAGATTTTTCAGTAGATAGAGTCAAGAAGATGTTGGACATGGCACTGCAAGGTGAAGAAGAGAAGAATGTTCAATTTGAGATCAGAACACATGATTTGAAGATTGATTCTGGTCCTATCAGCTTGGTAGTTAATGCTTGTGCAAGCAGGGATCTTCAAGATAATGTTGTGGGGGTTTGTTTTGTAGCCCAAGATATAACTGCTCAGAAGACAGTGATGGATAAATTCACCCGAATTGAAGGCGACTACAAGGCGATTGTACAGAACCGAAACCCATTGATCCCGCCAATATTTGGCACAGATGAATTTGGCTGGTGCTGTGAATGGAATTCTGCTATGACAAAGTTAACTGGATGGAAGAGAGAGGAGGTGATGGATAAAATGCTCTTAGGAGAGGTTTTTGGGACTCAGATAGCATGTTGTCGTCTAAGGAATCAAGAAGCTGTTGTTAATTTTAGCATTGTACTTAATAAAGCCATGACTGGTTTGGAAACTGAGAAGGTTCCTTTTGGTTTCTTTACTCGTAAGGGGAAGTATGTAGAATGCTTGCTATCTGTGAGTAAGAAATTGGATGGAGAAGGTGTAGTTACTGGGGTCTTTTGCTTCTTGCAACTAGCTAGTCCTGAGCTGCAACAAGCATTGCATATTCAGCACTTATCTGAACAAACTGCCTTGAAGAGACTGAAAGCTTTGACTTACCTGAAAAGGCAAATCCGGAATCCTTTATGTGGGATTGTGTTCTCCAGGAAATTGTTAGAGGGTACTGAGTTGGGAACTGAACAAAAACAACTTCTGCAAACTGGCACTCGGTGCCAACGCCAACTTAGCAAAATTCTGGATGACTCTGATCTTGACAGCATTATTGATGGATACTTGGATTTGGAGATGGTAGAATTTACTCTGCATGAAGTTTTGGTTGCCTCCCTAAGTCAAGTCATGACAAAGAGTAATGCAAAGGGTATCAGAGTAGTCAATGATGTTGAAGAACAGATCACAACAGAAACCTTACATGGTGATAGTCTTCGGCTTCAGCAGATCCTAGCCGACTTTTTATCGATTTCCATCAATTTCACCCCAACTGGAGGTCAAGTTGTTGTAGCAGCCTCATTAACCAAAGATCAGTTAGGGAAACTAGTCCATCTTGCTAATTTGGAGCTAAGGTAA

>XM_016084074.2 PREDICTED: Arachis duranensis phytochrome A (LOC107465088), mRNA

CATTTCATTCGACCATATTCACATGGTGTTTCTTTGTTTTTATAGTGCCCAACATTTATTTACTTATTTATAAATTTTATATATAATACAAGTTTGGTGTTATGGGGGTCCCCTCCTCAGTGATGAGCAGTAGTAGCAGCAGTGCTGAGTGCTGAACTTGGAAGTTGGATTTGGATTTGAGGGTTTCAATGTTTGGGGGGGACACACATAAGATTGCCGTTGATCGAGGTTTTCTTTCATGAATAGAGCTTCTTCTTCTTCTTCTTCAACTGCAGCAGGCACTTGCTTATTTTAAAGCCCAGCTTTTTCTTCCAACTTCCTCTCCTCATTGTCAACAACACTACAAGGCTTCTTCTTTCTTTTCGGTGAAAATGTCATCCTCAAGGCCTAGCCAATCTTCTAGCAATTCGGGGAGATCAAGACATAGCGCTAGAATTATTGCTCAGACCACTGTAGATGCAAAGCTCCATGCTAGTTTTGAGGAGTCTGGTAGTTCCTTTGACTACTCTAGTTCGGTGCGCGCCTCCGGCTCAGCTGATGGAGAAAATCAACCAAGAACAGATAAAGTAACAACAGCTTACCTCCATCACATACAGAAAGGCAAGATGATTCAACCCTTTGGGTGCTTGCTGGCCTTGGATGAGAAAACATGCAAGGTGATTGCTTACAGTGAGAATGCACCAGAGATGCTGACCATGGCGAGTCATGCTGTTCCCAGTGTTGGTGACCACCCTGCCCTTGGCATTGGCACCGACATAAGAACTATTTTCACTGCTCCAAGTGCTTCGGCATTGCAGAAAGCACTAGGATTTGGAGAAGTTCATCTTCTTAACCCCATCCTAGTTCATTGCAAGACTTCTGGGAAACCCTTCTATGCTATTCTCCATCGTGTTACTGGTAGTTTGATTATTGACTTTGAGCCCGTCAAGCCTTATGAAGTTCCTATGACTGCAGCAGGTGCTCTGCAATCCTACAAGCTTGCTGCGAAAGCAATCACCCGATTGCAGTCTTTGCCTAGTGGGAGCATGGAAAGGCTATGTGATACAATGGTTCAAGAAGTTTTTGAACTCACGGGTTATGACAGGGTGATGGCTTATAAATTTCATGAGGATGATCATGGGGAAGTGATTGCTGAGTTAACAAAGCCAGGACTTGAACCATATCTTGGGTTGCACTATCCGTCCACTGATATTCCCCAGGCTGCACGTTTTTTGTTCTTGAAGAACAAGGTCCGTATGATTGTTGATTGTCATGCAAAACATGTAAAGGTTGTTCAAGATGAGAAGCTGCCATTCGATTTGACTTTGTGTGGTTCAACCTTAAGAGCTCCTCATAGTTGCCATTTGCAATACATGTCAAACATGGATTCAATTGCTTCCCTAGTTATGGCAGTTATAGTCAATGACAGCGATGAAGATGCGGACAACTCTGATGCTGTTCAGCCACAAAAGAGAAAGCGACTCTGGGGCTTAGTTGTTTGTCATAACACTACTCCCAGGTTTGTTCCTTTTCCTCTAAGGTATGCTTGTGAGTTTCTGACACAAGTATTTGCCATCCATGTCAACAGAGAAATAGAGTTAGAATATCAGATTACTGAAAAGAATATCCTTCGCACTCAGACGCTCTTGTGTGATATGCTGATGCGAGATGCACCCCTAGGAATTGTATCACAAAGCCCTAATATAATGGACCTAGTTAAATGTGATGGGGCTTCCCTCTTGTATAAAAACAAGGTATGGAGATTAGGAGTAACACCTACTGAATCTCATATAAGAGAGATAGCTTTGTGGTTGTCTGAGCACCATATGGATTCCACAGGTCTTAGTACAGATAGCTTGTATGATGCCGGGTTCCCAGGGGCTCTGTCTCTTGGTGATGTAGTATGTGGAATGGCAGCGGTTAGAATAACTGAGAAAGACATAGTTTTCTGGTTTCGATCACACACAGCTGCAGAAATCAGATGGGGTGGTGCAAAGCATGACCCTGGTGAAAAGGACGACGGCAGGAGGATGCATCCACGGTCATCTTTCAAGGCTTTCCTTGAAGTTGTTAAGTCAAGGAGCTTACCGTGGAAAGACTACGAGATGGATGCTATTCATTCATTGCAACTAATACTAAGAAATGCATTCAAAGAGATGGATAGTATGGACATAACCACAAATGCAATAAATACAAGGCTGAATGATTTGAGGATTGAAGGGATGCAGGAACTGGAAGCCGTGACAAGTGAGATGGTTAGATTAATTGAAACTGCAACAGTACCTATTCTGGCTGTAGATGTTGATGGGCTGGTTAATGGATGGAATATAAAGATTGCCGAATTGACTGGCCTTTCGGTTGGTGATGCTATAGGGAAGCATCTTCTCACGCTCGTTGAGAACTCTTCAGTTGGTATAGTCAAGAAGATGCTTGAGATGGCATTGAAAGGTGAAGAAGAGAAAAACGTCCAATTTGAGATCAAAACACATGGGTCTAAAGTTGATTGTGGTCCTATTAGATTGGTAGTTAATGCTTGCGCAAGCCGGGATATTCATGATAATGTTGTGGGGGTTTGTTTTGTGGCCCAAGACATCACAGCTCAGAAGACTGTCATGGATAAATTTACCCGAATTGAAGGTGATTACAAGGCAATTGTACAGAATCCCAACCCATTGATCCCCCCAATATTTGGAACTGATGAATTTGGCTGGTGTTGTGAGTGGAATGCAGCTATGACAAAGGTGACCGGATGGAAGAGAGAAGAGGTGATGGATAAAATGCTTTTAGGAGAGGTTTTTGGGACTCAGATGGCTTGTTGTCGTCTTAAGAACCAAGAAGCTTTTGTTAATTTTGGTATTGTACTTAATAAAGCCATGACCGGTTTGGAGACAGCAAAGGTTGCTTTTGGTTTCTTTGCTCGTAGTGGGAAGTATGTAGAATGCCTGCTTTCGGTGAGTAAAAAATTGGACGTCGAAGGTGTAGTTACTGGGGTCTTCTGCTTCTTGCAGCTAGCTAGTCCAGAGCTGCAACAAGCATTGCATGTTCAGCGAATATCTGAACAAACTGCCTTGAAGAGACTGAAAGCTTTAACTTATATGAAGAGGCAGATCAGGAATCCTTTATCCGGGATGATGTTTTCGCGGAAAATGTTGGAGGCCACTGAGTTGGGAGCGGAACAAAAGCAACTCCTACACACCAGTGCTCAGTGCCAGTGCCAGCTTAGCAAAGTTCTTGATGACTCAGATCTTGACAGCATCATTGATGGTTACTTGGATCTTGAGATGGCTGAATTCACTCTGCATGATGTATTGGTTGCTTCCCTTAGTCAAGTAATGACAAAGAGTAACACCAAAGCTATCCGAATAGTCAACGATGTCAAAGAGCAAATTGTGACAGAAACCTTATACGGTGATAGTCTTAGGCTTCAGCAGGCCATAGCTGACTTCTTATTGATTTCCATAAACTTCACACCCAATGGAGGTCAAGTTGTAGTAACAGCCACTCTGACCAAAGAACAAATAGGGCAATCTGTCCATCTTGTTAATTTGGAGCTCAGCATAACACATGGTGGTAGTGGGGTGNCCATTCTAAAGCTGATGAATGGAGATGTGCGTTATGTAAGGGAAGCAGGCAAATCATCTTTTATCCTATCTGCTGAACTTGCCGCAGCTCATAAGTTGAAAGATTAGTAAGTTTTGGCCAAAAAAGGTTAGTTAATAAGATGTTACAAGATAATGTGCTTTTTGTACATCAGAAAGTGACTGGAATGAGAAATCAGTTGCATTCCTTTGTGCCTCTAGTTTGTTTCTGTAACTTATTGTAACGCATGCTGTAGATATGTATTAATTTCTTTCTCATTCCTTCATCAGTTCTTGCACTAGCGCTAGACACTGTTAACGACATTGATCTTTTAGGTCGGTTGTTGTCACGAATCGCACTTC

AGTTTGTTGGTTTGCAAATTTAAGCTCTCTTCGCCA

>KT984757.1 Arachis hypogaea cultivar HL14 phytochrome A-like protein mRNA, complete cds

ATGTCGTCGTCGAGGCGAAGCCAATCATCGAGCAATTCAAGCAGATCAAGACAGAGTGCAAGAGTAATTG

CTCAGACAAGTGTGGATGCAAAGCTGCATGCAAACTTTGAGGAGTCCGGAAGTTCATTCGACTACTCCAA

TTCGGTGCGGTTGTCTTCCGGTACAGTCAGTGGAGAAAACCAGGCAAGATGTGACAGAGTTACAGCAGCT

TATCTTCATCAAATGCAGAAGGGGAAGTTCATCCAGCCCTTTGGGTGCTTGCTAGCTTTGGATGACAAGA

CACTTAGGGTCATTGCATACAGTCACAATGCATCTGAAATGCTTACCATGGTCAGCCATGCTGTTCCAAG

TGTTGGTGATCACACTGCTCTTGCCATTGGCACCGACATTCGGACTATTTTCACGCCCTCAAGTGCTGCT

GCTTTGCAAAAGGCCCTTGCAGTTCCGGAGGTTTCGCTTCTTAACCCCATTCTAGTTCATTGCAAGACTT

CTGGCAAGCCCTTCTATGCAATTGTTCATCGTATTACCGCTACTTTGATCATTGATTTTGAGCCTGTTAA

GCCTCATGAAGTTCCTATGACTGCCGCCGGAGCCCTGCAATCTTACAAGCTTGCGGCCAAAGCAATAACT

AGATTGCAGTCTTTGCCCAGTGGGAGCATGGAAACGCTTTGTGACACCATGGTTCAAGAAGATTTTGAGC

TCACGGGTTATGACAGGGTGATGGCTTATAAATTTCATGAGGATGATCATGGGGAAGTGATTGCTGAGGT

TGCAAAGCCAGGTCTTGAGCCATATCTGGGTTTGCACTATCCAGCCACTGATATTCCCCAGGCTGCGCGC

TTCTTGTTTATGAAGAACAAGGTCCGAATGATAGTTGATTGTCGTGCAAAGCATGTGAAGGTGCTTCAAG

ACCCCAAAGTTTCCATTGATTTAACTTTGTGCGGTTCAACTTTAAGGGCTGCTCATAGTTGTCACTTGCA

ATATATGGAAAATATGAATTCAATTGCTTCCCTGGTTATGGCAGTTGTGGTCAATGATAACGATGAAGAT

GGGGATGGCTCTGATGTTGTTCAGCCACAAAAGAGAAAGAGACTTTGGGGTTTAGTAGTTTGCCATAACA

CTACTCCAAGGTTTGTTCCCTTTCCTCTAAGGTATGCTTGTGAGTTTTTGGCTCAAGTATTTGCCATCCA

TGTGAATAAGGAATTAGAGTTAGAATATCAGATTGTTGAGAAGAATATCCTGCGCACGCAGACACTCTTG

TGCGATATGCTGATGCGAGATGCACCCCTTGGTATTGTATCACAGAGCCCTAATATAATGGATCTTGTGA

GATGTGATGGAGCAGCACTCTTGTATAGAGACAAGGTATGGAGATTAGGTGTGGCTCCAAGTGAATCTCA

CATAAGAGAGTTAGCTTTATGGCTCTCTAAGTGCCACAAGGACTCCACGGGTTTGAGTACAGATAGCTTG

TCTGATGCAGGCTTCCCAGGAGCTGCTGCTCTTGGTGATGTTGTTTGTGGAATGGCAGCTGTGAGAATTT

CTTCCATGGACATAGTTTTCTGGTTTAGATCACACACTGCTGCAGAAATCAGATGGGGTGGTGCCAAGCA

TGAACCTGGTGACAGGGATGATCCTACAAAGATGAGTCCAAGATCATCATTCAAGGCTTTCCTTGAAGTT

GTGAAGGGAAGGAGCTTACCATGGAAGGACTACGAAATGGATGCTATCCATTCATTGCAGCTAATACTGA

GAAATTCATTCAAAGATAATGAGATTATGGATATAAGCACGCAAGCTATTGATACAAGACTAAATGATTT

GAAGATTGAAGGGATGCAAGAATTAGAAGCAGTGACGAGTGAGATGGTTCGATTAATTGAAACAGCATCA

GTGCCAATTTTGGCAGTTAATGTTGATGGGATGGTCAATGGATGGAACACGAAGATTGCTGAGTTGACAG

GCCTTTCAGTTGAGGAAGCTATTGGAAAGGATTTACTTACGCTGGTTGAGGATTTTTCAGCAGAGAGAGT

CAAGAAGATGCTGGACATGGCACTGCAGGGTAAAGAAGAGAAGAATTTCCAATTTGAGATCAAAACACAT

GGTGTGAAGATTGATTCAGGTCCTATCAGCTTGGTAGTTAATGCTTGTGCAAGCAGGAATCTCCAAAACA

GCGTGGTGGGAGTTTGTTTTGTGGCCCATGATATGACTGCTGAGAAGACAGTCATGGACAAATTCACTCG

AATTGAAGGTGACTATAGGGCGATTGTACAGAATCCGAACCCTTTGATCCCCCCAATATTTGGCACAGAT

GAATTTGGCTGGTGTTGTGAATGGAATTCAGCCATGACAAAGCTAACTGGATGGAAGAGAGAGGATGTGA

TGGATAAAATGCTATTAGGGGAGGTTTTCGGAACCCATATGGCTTGTTGCCGTCTTAAGAATCAAGAAGC

TGTTGTTAATTTTGGAATTGTACTTAACAATGCCATGACTGGTGTGGAAACAGAGAAGGCTGCTTTTGGT

TTCTTCACTCGGAAGGGCAAGTATGTAGAATGCTTGCTTTCTGTGAGCAAGAAATTGGATGTAGAGGGTG

AAGTTACTGGAGTCTTCTGCTTCTTACAGACAGCTAGCCCTGAGCTTCAACAAGCATTGCATATTCAGCG

TTTATCCGAACAAACTGCCTTGAAGAGACTCAAAGCTTTAACTTATATGAAAAGGCAAATTCGGAATCCT

TTGTGTGGGATTGTGTTCTCTCGAAAATTGTTGGAGAATACTGAGTTGGGAATTGAGCAAAAACAACTTC

TGGACACTGGCACTCAGTGCCAACGCCAGCTTAGCAAAATTCTCGATGACTCGGATCTCGACCGTATCAT

TGATGGCTACTTGGATTTGGAAATGGTTGAATTCACTCTGCATCAAGTTTTTGTTGCCTGTCTAAGTCAG

GTCATGACAAAGAGCAAGGCAATGGGTATCCATATAATCAACGAAGTCACGGAGCACATCATGACAGAAA

CCTTATATGGTGATAGTCTTAGGCTGCAGCAAGTCTTGGCTGACTTCTTATTGGTTTGCATCAATTTTAC

GCCAACCGGAGGTCAAGTTGTTGTTGCAGCCTCTTTAACCAAAGATCAGTTAGGCAAATCAGTTCATTTG

GCTAACTTGGAGATAAGCATAACACATGATGGTGTTGGTGTTCCGGAAACATTGCTGAACCAAATGTTCG

GACGAGACGGACAAGAATCCGAGGAGGGTATTAGTCTGCTCATCAGCAGAAAGTTGCTGAAGCTGATGAA

TGGAGACGTGCGGTATCTGAGGGAAGCTGGCAAATCATCTTTCATCTTAACAGTTGAACTGGCTGCTTCC

CAGAAATTGATTGCTTAA

>XM_021103955.1 PREDICTED: Arachis ipaensis phytochrome A (LOC107645995), transcript variant X1, mRNA

AAATTAGATATGACATGGCAGTGGGTGGTGTTTGAATCTGAAGTGAGGATTTGGAGTAGTTATCCTCACT

TGCCACCAAAAACTCATTAACTCATAAATATCCATCTCTGCCTTTTCATGCCTTCACTTTCACGCCATAC

ATAGATGCTGCTTCTGCTTCATCTTCTTCATCTGCATAATTCATAGTTCCAAGAATGTCGTCGTCGAGGC

GAAGCCAATCATCGAGCAATTCAAGCAGATCAAGACAGAGTGCAAGAGTAATTGCTCAGACAAGTGTGGA

TGCAAAGCTGCATGCAAACTTTGAGGAGTCCGGAAGTTCATTCGACTACTCGAATTCGGTGCGGTTGTCT

TCCGGTACAGCCAGTGGAGAAAAGCAAGCAAGGTGTGACAGAGTTACAGCAGCTTATCTTCATCAAATGC

AGAAGGGGAAGTTCATCCAGCCCTTTGGGTGCTTGCTAGCTTTGGATGACAAGACACTTAGGGTCATTGC

ATACAGTCACAATGCATCTGAAATGCTTACCATGGTCAGCCATGCTGTCCCAAGTGTTGGTGATCACCCT

GCTCTTGCCATTGGCACCGACATTCGGACTATTTTCACGCCCTCAAGTGCTGCTGCTTTGCAAAAGGCCC

TTGCAGTTCCGGAGGTTTCGCTTCTTAACCCCATTCTAGTTCATTGCAAGACTTCTGGCAAGCCCTTCTA

TGCAATTGTTCATCGTATTACCGCTAGTTTGATCATTGATTTTGAGCCTGTTAAGCCTCATGAAGTTCCC

ATGACTGCCGCCGGAGCGCTGCAATCTTACAAGCTTGCGGCCAAAGCAATAACTAGATTGCAGTCTTTGC

CCAGTGGGAGCATGGAAACGCTTTGTGACACCATGGTTCAAGAAGTTTTTGAGCTCACGGGTTATGACAG

GGTGATGGCTTATAAATTTCATGAGGATGATCATGGGGAAGTGATTGCTGAGGTTGCAAAACCAGGCCTT

GAGCCATATCTGGGTTTGCACTATCCAGCAACTGATATCCCCCAGGCTGCGCGCTTCTTGTTTATGAAAA

ACAAGGTCCGAATGATAGTTGATTGTCGTGCAAAGCATGTGAAGGTGCTTCAAGACCCCAAAGTTTCCAT

TGACTTAACTTTGTGCGGTTCAACTTTAAGGGCTGCTCATAGTTGCCACTTGCAATATATGGAGAATATG

AATTCAATTGCTTCCCTGGTTTTGGCAGTTGTGGTCAATGATAACGATGAAGATGGGGATGGCTCTGATG

TTGTTCAGCCACAAAAGAGAAAGAGACTTTGGGGTTTAGTAGTTTGCCATAACACTACTCCAAGGTTTGT

TCCCTTTCCTCTAAGGTATGCTTGTGAATTTTTGGCTCAAGTATTTGCCATCCATGTGAATAAGGAATTA

GAGTTAGAATATCAGATTGTTGAGAAGAATATCCTGCGCACGCAGACACTCTTGTGCGATATGCTGATGC

GAGATGCACCCCTTGGTATTGTATCACAGAGCCCTAATATAATGGATCTTGTGAGATGTGATGGAGCAGC

ACTCTTGTATAGAGACAAGGTATGGAGATTAGGTGTGGCTCCAAGTGAATCTCACATAAGAGAGTTAGCT

TTATGGCTCTCTAAGTGCCACAAGGACTCCACGGGTTTGAGTACAGATAGCTTGTCTGATGCAGGCTTCC

CAGGAGCTGCTGCTCTTGGTGATGTTGTTTGTGGAATGGCAGCTGTGAGAATTTCTTCCATGGACATAGT

TTTCTGGTTTAGATCACACACTGCTGCAGAAATCAGATGGGGTGGTGCCAAGCATGAACCTGGTGACAGG

GATGATCCTACAAAGATGAGTCCAAGATCATCATTCAAGGCTTTCCTTGAAGTTGTGAAGGGAAGGAGCT

TACCATGGAAGGACTACGAAATGGATGCTATCCATTCATTGCAGCTAATACTGAGAAATTCGTTCAAAGA

TAATGAGATTATGGATATAAGCACGCAAGCTATTGATACAAGACTAAATGATTTGAAGATTGAAGGGATG

CAAGAACTAGAAGCAGTGACGAGTGAGATGGTTCGATTAATTGAAACAGCATCAGTGCCAATTTTGGCAG

TTAATGTTGATGGGATGGTCAATGGATGGAACACGAAGATTGCTGAGTTGACAGGCCTTTCAGTTGAGGA

AGCCATTGGAAAGGATTTACTTACGCTGGTTGAGGATTTTTCAGTAGAGAGAGTCAAGAAGATGCTGGAC

ATGGCACTGCAGGGTAAAGAAGAGAAGAATTTCCAATTTGAGATCAAAACACATGGTGTGAAGATTGATT

CAGGTCCTATCAGCTTGGTAGTTAATGCTTGTGCAAGCAGGAATCTCCAAAACAGCGTGGTGGGAGTTTG

TTTTGTGGCCCATGATATGACTGCTGAGAAGACAGTCATGGACAAATTCACTCGAATTGAAGGTGACTAT

AGGGCGATTGTACAGAATCCGAACCCTTTGATCCCCCCAATATTTGGCACAGATGAATTTGGCTGGTGTT

GTGAATGGAATTCAGCCATGACAAAGTTAACTGGATGGAAGAGAGAGGATGTGATGGATAAAATGCTATT

AGGGGAGGTTTTTGGAACCCATATGGCTTGTTGCCGTCTTAAGAATCAAGAAGCTGTTGTTAATTTTGGA

ATTGTACTTAACAATGCCATGACTGGTGTGGAAACAGAGAAAGCTGCTTTTGGTTTCTTCACTCGGAAGG

GCAAGTATGTAGAATGCTTGCTTTCTGTGAGCAAGAAATTGGATGTAGAGGGTGAAGTTACTGGAGTCTT

CTGCTTCTTACAGACAGCTAGCCCTGAGCTTCAACAAGCATTGCATATTCAGCGTTTGTCCGAACAAACT

GCATTGAAGAGACTCAAAGCTTTAACTTATATGAAAAGGAAAATTCGGAATCCTTTGTGTGGGATTGTGT

TCTCTCGAAAATTGTTGGAGAATACTGAGTTGGGAATTGAGCAAAAACAACTTCTGGACACTGGCACTCA

GTGCCAACGCCAGCTTAGCAAAATTCTCGATGACTCGGATCTTGACCGTATCATTGATGGCTACTTGGAT

TTGGAAATGGTTGAATTCACTCTGCATCAAGTTTTTGTTGCCTGCCTAAGTCAGGTCATGACAAAGAGCA

AGGCAATGGGTATCCATATAATCAACGAAGTCACGGAGCACATCATGACAGAAACCTTATATGGTGATAG

TCTTAGGCTGCAGCAAGTCTTGGCTGACTTCTTATTGATTTGCATCAATTTTACGCCAACCGGAGGTCAA

GTTGTTGTTGCAGCCTCTTTAACCAAAGATCAATTAGGCAAATCAGTTCATTTGGCTAACTTGGAGATAA

GCATAACACATGATGGTGTTGGTGTTCCGGAAACATTGCTGAACCAAATGTTCGGACGAGACGGACAAGA

ATCCGAGGAGGGTATTAGTCTGCTCATCAGCAGAAAGTTGCTGAAGCTGATGAATGGAGACGTGCGGTAT

CTGAGGGAAGCTGGCAAATCATCTTTCATCTTAACAGTTGAACTGGCTGCTTCCCAGAAATTGATTGCTT

AAAGTTATAGGAAAATAAAGAAATACTTTCAGTTTTTGTACATCAGAAAAGCAAGTGTGCAAGAAATCAC

TTTTCTTGTACAACATACCTTAAGAGTAAGAGTAACATA

>NM_100828.4 Arabidopsis thaliana phytochrome A (PHYA), mRNA

CCTCAAAACCCAACTTTCTACTTCTGGTTACTGCCTTCATTAAACAACTACCACCATCTTTCTTTCTCTC

TCTCTTTCTCGTTTTTTTATTTTATTAATAATCTTTTTTTTTTTTTTTACTTTTGATCTCTTATCCTTTG

GACGATTCTCTTCAATCGGATTTATAAGAATCAACCCCATTTTGTTCTTCTTCCACTTTACTTCTTTTTT

CCTCATTACACAGTCTTAGTCACATCGGTGAAAATATCAATTAATATTCCCCAGAAAGTTGCCAAGATTT

ACAAGTAGTAGTACTTGATATATCTTAAGCCCACTGTTCTGTTTTAGCGAGGAAAATGTTACCGGCGAAG

CCGACCCTGTTCTTGATATGTGTGTGAAGTTTGTCTTTTATCCAAATTAAGCTTTTTTCTTCTTTTATTT

TATTCCATTATAAAATATACAAATCGCCCATACTCCGAGACATGGGCATACGTATTCGGGGTGGTCCCAT

TCATCGTCGTCGTCTGTGTTAGGGAGCACAAATAATAGAGAGGCGTAGCACAAGAGAGAAGGTGGTGATC

GAGGCCAAGATTTAGAATTTAACTATAACAAAAAGCCTCTGACGAGTGTGACTAGTCACAAGATCTGATC

ATGGCTTCTTGAAACTTCTTCTTCTTCTTTCTTCTCTTTAAAGGAAAAAAATGTCAGGCTCTAGGCCGAC

TCAGTCCTCTGAGGGCTCAAGGCGATCAAGGCACAGCGCTAGGATCATTGCGCAGACCACTGTAGATGCG

AAACTCCATGCTGATTTTGAGGAGTCAGGCAGCTCCTTTGATTACTCAACCTCAGTGCGTGTCACTGGCC

CGGTTGTGGAGAATCAGCCACCAAGGTCTGACAAAGTTACCACGACTTATCTTCATCATATACAGAAGGG

AAAGCTGATTCAGCCCTTCGGTTGTTTACTTGCCTTGGATGAGAAGACCTTCAAAGTTATTGCATACAGC

GAGAATGCATCTGAGCTGTTGACAATGGCCAGTCATGCAGTTCCTAGTGTTGGCGAACACCCTGTTCTAG

GCATTGGGACAGATATAAGGAGTCTTTTCACTGCTCCTAGTGCGTCTGCATTGCAGAAAGCCCTTGGATT

TGGAGATGTCTCTCTTTTGAATCCCATTCTTGTGCACTGCAGGACTTCTGCAAAGCCCTTTTATGCGATT

ATCCACAGGGTTACAGGGAGCATCATCATCGACTTTGAACCCGTGAAGCCTTATGAAGTCCCCATGACAG

CTGCTGGTGCCTTACAATCATACAAGCTCGCTGCCAAAGCAATCACTAGGCTGCAATCTTTACCCAGCGG

GAGTATGGAAAGGCTTTGTGATACAATGGTTCAAGAGGTTTTTGAACTCACGGGGTATGACAGGGTGATG

GCTTATAAGTTTCATGAAGATGATCACGGTGAGGTTGTCTCCGAGGTTACAAAACCTGGGCTGGAGCCTT

ATCTTGGGCTGCATTATCCTGCCACCGACATCCCTCAAGCAGCCCGTTTTCTGTTTATGAAGAACAAGGT

CCGGATGATAGTTGATTGCAATGCAAAACATGCTAGGGTGCTTCAAGATGAAAAGCTTTCCTTTGACCTT

ACCTTGTGTGGCTCCACCCTTAGAGCACCGCACAGCTGCCATTTGCAGTACATGGCCAACATGGATTCAA

TTGCATCTCTGGTTATGGCGGTTGTAGTTAACGAGGAAGATGGAGAAGGGGATGCTCCTGATGCTACTAC

ACAGCCTCAAAAGAGAAAGAGACTATGGGGTTTAGTGGTTTGTCACAATACGACTCCGAGGTTTGTTCCA

TTTCCTCTCAGGTATGCCTGTGAGTTTCTAGCTCAAGTGTTTGCCATACACGTCAATAAGGAGGTGGAAC

TCGATAACCAGATGGTGGAGAAGAACATTTTGCGCACGCAGACACTCTTGTGCGATATGCTGATGCGTGA

TGCTCCACTGGGTATTGTGTCGCAAAGCCCCAACATAATGGACCTTGTGAAATGTGATGGAGCAGCTCTC

TTGTATAAAGACAAGATATGGAAACTGGGAACAACTCCAAGTGAGTTCCACCTGCAGGAGATAGCTTCAT

GGTTGTGTGAATACCACATGGATTCAACGGGTTTGAGCACTGATAGTTTGCATGACGCCGGGTTTCCTAG

GGCTCTATCTCTCGGGGATTCGGTATGTGGGATGGCAGCTGTGAGGATATCATCGAAAGACATGATTTTC

TGGTTCCGTTCTCATACCGCTGGTGAAGTGAGATGGGGAGGTGCGAAGCATGATCCAGATGATAGGGATG

ATGCAAGGAGAATGCACCCAAGGTCATCGTTCAAGGCTTTCCTTGAAGTGGTCAAGACAAGGAGTTTACC

TTGGAAGGACTATGAGATGGATGCCATACACTCCTTGCAACTTATTTTGAGGAATGCTTTCAAGGATAGT

GAAACTACTGATGTGAATACAAAGGTCATTTACTCGAAGCTAAATGATCTCAAAATTGATGGTATACAAG

AACTAGAAGCTGTGACCAGTGAGATGGTTCGTTTAATTGAGACTGCTACGGTGCCAATATTGGCGGTTGA

TTCTGATGGACTGGTTAATGGTTGGAACACGAAAATTGCTGAGCTGACTGGTCTTTCGGTTGATGAAGCA

ATCGGGAAGCATTTCCTCACACTTGTTGAAGATTCTTCAGTGGAAATCGTTAAAAGGATGCTAGAGAACG

CATTAGAAGGAACTGAGGAGCAGAATGTCCAGTTTGAGATCAAGACACATCTGTCCAGGGCTGATGCTGG

GCCAATAAGTTTAGTTGTAAATGCATGCGCAAGTAGAGATCTCCATGAAAACGTGGTTGGGGTGTGTTTT

GTAGCCCATGATCTTACTGGCCAGAAGACTGTGATGGACAAGTTTACGCGGATTGAAGGTGATTACAAGG

CAATCATCCAAAATCCAAACCCGCTGATCCCGCCAATATTTGGTACCGATGAGTTTGGATGGTGCACAGA

GTGGAATCCAGCAATGTCAAAGTTAACCGGTTTGAAGCGAGAGGAAGTGATTGACAAAATGCTCTTAGGA

GAAGTATTTGGGACGCAGAAGTCATGTTGTCGTCTAAAGAATCAAGAAGCCTTTGTAAACCTTGGGATTG

TGCTGAACAATGCTGTGACCAGTCAAGATCCAGAGAAAGTATCGTTTGCTTTCTTTACAAGAGGTGGCAA

GTATGTGGAGTGTCTGTTGTGTGTGAGTAAGAAACTGGACAGGGAAGGTGTAGTGACAGGTGTCTTCTGT

TTCCTGCAACTTGCCAGCCATGAGCTGCAGCAAGCGCTCCATGTTCAACGTTTAGCTGAGCGAACCGCAG

TGAAGAGACTAAAGGCTCTAGCATACATAAAAAGACAGATCAGGAATCCGCTATCTGGGATCATGTTTAC

AAGGAAAATGATAGAGGGTACTGAATTAGGACCAGAGCAAAGACGGATTTTGCAAACTAGCGCGTTATGT

CAGAAGCAACTAAGCAAGATCCTCGATGATTCGGATCTTGAAAGCATCATTGAAGGATGCTTGGATTTGG

AAATGAAAGAATTCACCTTAAATGAAGTGTTGACTGCTTCCACAAGTCAAGTAATGATGAAGAGTAACGG

AAAGAGTGTTCGGATAACAAATGAGACCGGAGAAGAAGTAATGTCTGACACTTTGTATGGAGACAGTATT

AGGCTTCAACAAGTCTTGGCAGATTTCATGCTGATGGCTGTAAACTTTACACCATCCGGAGGTCAGCTAA

CTGTTTCAGCTTCCCTGAGGAAGGATCAGCTCGGGCGTTCTGTGCATCTTGCTAATCTAGAGATCAGGTT

AACGCATACCGGAGCTGGGATACCTGAGTTTTTACTAAACCAAATGTTTGGGACTGAGGAAGATGTGTCA

GAAGAAGGATTGAGCTTAATGGTTAGCCGGAAACTGGTGAAGCTGATGAATGGAGATGTTCAGTACTTGA

GACAAGCTGGGAAATCAAGTTTCATTATCACTGCGGAACTCGCTGCAGCAAACAAGTAGTCCCCAAAAGA

AAAGGGGTCTGGCTTGATATAAAATAGTCACTGGTTGTTCTTTGCTTGTAACTTTCCTTATCGCTTTTGT

TTTCGTTTTCAAATTTCAGTAACGATGAAATATCCATCCATTTACATCTTCTGTTGAACTCTTTTCTGAA

GCTGTAAATATGGATGCATATCTAATCTCCTCCTGAGTGTTTTGGTTTCATGATGTATCATTCAAAATAA

GTAATATAGGAGAAATGAGCTAATGTTTACGGTCCATGGTTTATCATAATGGCAGAAACTCAAACAAATA

TTCTATCTAGTTTCATATAATCTCCTTCTTTTAAGGTATTAGAGGTA

>XM_020355232.2 PREDICTED: Cajanus cajan phytochrome A (LOC109795716), transcript variant X2, mRNA

ATTTCATGGTTTGATTGTTCGTCTGAGTTATGAATTGTAATGGAACACAATGTGGATAGCTTTGTTACTG

TTACTTTTTATCCTCCCAGCTTTGAGGGCTGCACGTGACACCCATAACTCTTTTTCTTGTTGCCATTCGC

TACGCTTCCTCTTCTATGGATATTGCTATCCAATGAAATAAATGAGTGACCCAGAAAAAGTTTCCTCATA

GCACGGTAATAACCCTTCATTAGAATTTGTTAAGCAACATAAGTTTGGATTTTTCCTCTCTTAAATTTGT

ACCCAAAAAGGAAAGTCAGAGTTGAAGGTGGTGATGATGCAGATCGTGATCAGAAGCCGTAAAAGCTGCG

TATTGCTATTTTTGGGTGCAATCATCAATTCTGTATTTACCAATTTAGAAACCAATCCATTCTTTAATAG

TAGCAATATTATAATATATTATTATAATGATTGAATAATAATAAATATAAGTAAGCTCCATAAAGAAAGA

GAGAGAGAGAGAGTAGTATTAAATTATTATATCATTGCTATTATGGCATGGCAGTATCTACTGTGTCTCA

GTTGTAACTGCTGAGTTCTGTTCTGTCTAAGTCGTGAGCTTGGAGGGGTTCCTACTTTCAAGTTTCAACT

TTTGCCGAGTTCCAATCCCAAAGATTTTGTGCTTCATCACAATCAGTCATATAGATGCAGCTGTAACAAA

AAGTGGCAAGCTGGGATTTTTATTCCCAAGCTGCTTCTGCTTCTGTTAAAACCTTCTCTCTGTTCATTGG

TTGGAATCAGAATTTTCCTTGCAAGTAGAGGTGACAATGTCCTCCTCAAGGCCTAGCCAATCATCCAGCA

ACAATTCTGGCAGATCATCAAGGCACAGTGCTAGGGTTATTGCTCAGACAACTTTAGATGCAAAACTGCA

TGCAACTTTCGAGGAATCAGGTAGCTCTTTTGACTACTCCAATTCTGTGAGATTGTCTCCTGGTACAGGC

ACTGCCAGTGGAGACAATCAACCAAGGTCTGATAAAGTAACAACTGCTTATCTTCATCAGATTCAGAAAA

GCAAGCTTATCCAGCCATTTGGGTGCTTGCTAGCATTAGATGAGAAAACATGTAAGGTCATTGCTTACAG

TGAGAATGCACCTGAAATGCTCACTATGGTTAGTCATGCTGTGCCAAGTGTTGGAGACCACCCTGCCCTT

GGCATTGGCACTGACATAAGAACTATTTTCACTGCCCCAAGTGCTGCAGCTATTCAGAAGGCACTAGGTT

TTGGAGAGGTTTCACTTCTTAACCCCATTCTAGTCCATTGCAAGACCTCTGGGAAGCCCTTTTATGCAAT

TGTCCATCGTGTTACCGGAAGCGTTATCATTGATTTTGAGCCTGTCAAGCCTCATGAAGTTCCCATGACT

GCAGCAGGTGCCCTGCAATCCTACAAGCTTGCAGCAAAAGCAATAACTAGGTTGCAATCTTTGCCTAGTG

GGAGCTTGGAAACACTGTGTGACACAATGGTTCAAGAAGTTTTTGAACTCACAGGCTATGACAGGGTAAT

GGCTTATAAATTTCATGATGATGATCACGGTGAAGTGATTGCTGAGGTGAAAAGGCCAGGCCTAGAGCCA

TATCTGGGGTTGCACTACCCAGCCACTGATATTCCCCAAGTTACACGCTTTTTGTTTATGAAGAATAAGG

TGCGTATGATAGTTGATTGTCGTGCAAAGCATGTTAAGGTGCTTCAGGACAAAAAGATTCCATTTGATTT

AACCTTGTGTGGATCAACCTTGAGGGCTGCTCACAGTTGCCACTTGCAATACATGGAGAATATGAAATCT

AGTGCTTCCTTGGTTATGGCAGTTGTGGTCAATGACAATGATGAAGATGGGGATAGTGGCTCTGATGCTG

TTCAGCCACAGAAGAGAAAGAGACTCTGGGGTTTAGTGGTTTGCCATCACACTACTCCCAGGTTTGTTCC

TTTCCCTCTTAGGTATGCTTGTGAATTCCTGGCTCAAGTATTTGCCATCCATGTGAACAAAGAACTTGAG

ATAGAGTATCAGATTGTTGAGAAGAACATCCTGAGGACTCAAACACTCTTGTGTGACATGCTGATGCGAG

ATGCGCCCCTAGGCATAGTGTCACAGAGTCCTAACATAATGGATCTTGTTAAGTGTGATGGAGCAGCCTT

GTTGTACAAAAACAAGGTATGGAGATTAGGGGTAACACCAAGTGAATCTCAGATAAGAGAGATAGCATTG

TGGCTCTCTGAGTTCCATAGGGATTCCACAGGTTTGAGTACAGATAGCTTGTCTGATGCAGGCTTCCCAG

GGGCTGCTGCACTTGGTGATATTGCTTGTGGAATGGCAGCTGTCAGAATAGCTTCCAAAGATATAGTGTT

CTGGTTTCGGTCTCGCACTGCGGCTGAAATCCGATGGGGTGGTGCAAAGCATGAGCCTGGTGAAAAGGAT

GATGGTAGGAGGATGCATCCAAGATCATCATTCAAGGCTTTCCTTGAAGTTGTGAAGACTAGGAGCTTGC

CCTGGAAGGACTATGAAATGGATGCCATTCATTCATTGCAGTTAATTTTGAGAAATGCATTCAAAGAGAC

AGAGAGTATGGAGATAAGCACATATGCTATCAATACAAGACTAGGTGATTTGAAGATTGAAGGGATGCAA

GAGCTGGAAGCAGTGACAAGTGAGATGGTGAGGTTAATTGAAACAGCAACGGTGCCAATTCTGGCAGTTG

ATGTTAATGGGATGGTCAATGGATGGAATACAAAAATTGCTGAGTTGACAGGTCTTCCAGTTGAGAAAGC

TATTGGGAAGCATTTACTCATGCTTGTTGAGGATTTTTCTGTAGATAGAGTCAAGAAGATGTTGGACATG

GCACTCCAGGGTGAGGAAGAGAAAAATGTCCAATTTGAGATCAAAACACATGACTTGAAGATTGATTCTG

GTCCTATAAGCTTGGTCGTTAATGCTTGTGCAAGCAGGGATCTTCAAGACAATGTTGTGGGTGTTTGTTT

CGTGGCACAAGATATAACTGCTCAGAAGACAGTGATGGACAAATTCACCCGAATTGAAGGTGATTACAAG

GCAATTGTACAGAATCCAAATCCATTGATCCCTCCAATATTTGGTACAGATGAATTTGGCTGGTGTTGTG

AATGGAACTCAGCTATGATAAAATTAACTGGATGGAAACGAGAAGAGGTGATGGATAAAATGCTTTTAGG

AGAGGTTTTTGGGACCCAAATAGCTTCTTGTCGTCTAAGGAACCAAGAAGCTGTTGTTAATTTTAGCATT

GTACTTAACAAAGCCATGGCTGGTTTGGAAACAGAGAAGGTTCCCTTTGGTTTCTATACTCGTGAGGGGA

AGTATGTAGAATGTCTTCTTTCTGTGAGTAAGAAATTGGATGCAGAGGGCGTAGTTACTGGGGTATTCTG

CTTCTTGCAATTAGCTAGTCCAGAACTGCAACAGGCGTTACACATTCAGCACCTATCTGAACAAACTGCA

TTGAAGAGACTGAAAGATTTAACTTATTTGAAGAGGCAAATCCGGAATCCTCTATATGGGATTATATTCA

CCCGAAAATTGTTAGAGGGTACTGAGTTGGGAGCTGAACAAAATCAATTTCTGCAAACGGGCACTAGGTG

TCAACACCAGCTCAGTAAAATTCTGGATGACTCAGATCTTGACAGCATCATTGATGGTTACCTGGATTTG

GAGATGGTTGAATTTACTATGCATGAAGTTTTGGTTGCCTCCCTAAGTCAAGTGATGACAAAGAGTAATG

CAAAGGGTATCCCTGTAGTCAACCATGTTGAAGATCATATTACAACAGAAACCTTATATGGTGATAGTCT

CAGGCTTCAGCAGGTCTTAGCTGACTTTTTATCGATTTCCATCGATTTCACACCAAGCGGAGGTCAGGTT

GTTGTAGCAGCCTCATTAACCAAACAGCAGTTAGGGAAACTAGTCCATCTCGCTTATTTGGAACTCAGAA

ACATTGCTGAACCAGATGTTTGGACGTGATGGAAATGAATCTGAAGAGGGTATTAGCATGCTGATTAGCA

GAAAACTGCTAAAGCTGATGAATGGAGACGTACGTTATATAAGAGAAGCGGGAAAATCATCTTTCATCTT

AACTGTTGAACTTGCCGTAGCCCATAATTCCAGGGCTTGAAACTGGAAATAAAATCAAACCACCTTTTTT

GTACATCAGAATGTGAAGGAAGCGACAAATTTGCATTCCTTGTTCAATATTTAACTCTATTTTTTCTTTC

TTTTTTTTCTGGGCACGTTTCTGTACCTTCATGCAAGGTGTTGTAGATATGTATAACTAACTATTCACAC

ACTCTATTGACCTGTTCTCATCTACAGGCTACAGCAATTATGCTTCATTTGCAACGAAATGAAACATAGG

AACTAACTAAACTAGGAGCATGTTAAATGACA

>AB797198.1 Glycine max GmPhyA3 gene for phytochrome A, complete cds, cultivar: Harosoy

CTTTGATTGTCATACAAATTTAACCGATTGTAACAACTGTCTTAAAGAATTGTATATTTAATATTTTGTC

CATAAAATACCTAAATTATACTTTTGACAACAAATATCTGCAGACTGTAACGAAGCTCATTACATGTTTT

TTTCCCAACCTAACATTAATGTCAATGATACCAACTAGGAATATGCCATTAACATAGGAAGCTAAAAGCT

TTTAAGCTGCAACCTAATTAGGCACTTTTCGTACTAGATATGTCAACAACTCCACTCCACCAATCACCAT

CAAGAATCCGTTTCCTCAGCAGTCGGCACTACAATTAATTTTGAATGGTGCTGTGGGGTTTGGAGGAAGG

ATCATAAAGAAAAGATTTAGAGAAAGTGGGGAAAGCTTGAGAATTAGTGGATGGATCTGAGAGACGAACC

AATAAATCAATGGAACAGATATTGAATTTGCATATGGGTGGGGTAAGGATCATGATGGAGTAGGGAATAG

CCAGCAAGAGAATGAGAAGAATGTAATTAAGGAGAAACTAAAACTGTAATTTGCACACGTTAATGAGGCT

TACTACTTCTTTAACAAGTTGCCCACCGTTCATCTCATTCACATTCTTTATGACAAATGTTAACCAAAAT

CTTTACACTACTAACTAAGAAAATTACAAATTAGATTTTCATTAAAAGTTATTGTTAAATTCAATTTATC

ATAATTTCTATTACACCCTGATTTTAATAAAAATTTATGTTTGATCTTTAATAAGTGTGCCTTGAGGATA

TTAGATAGCAACATCCTTTACTATTAAATCCATAATTAATTTTTGCTGGATCATAATCATTGAATATTTC

GTAATTTACTTTGGGCGGTAATTTTTACTAACAGATCATTTGTTTAAACTTTATACGACAGGAAGAAGCC

TAACGCTATTATTTTTTACGAACTAGTTTGTTTAAAGTAAAAAAGTCATTTGATGATTTTAATTTTTTTG

CAAAAGGTTATAATTTTTAACTTTTAAAAAAATAAAACAATTTTCTTGAGGATAAAAATTATATGATTTA

AAATTATCTTTGAATTAAGAAGTATTTTAAAAAATTGAATCTTATAATATAAACTAATAAATAAAAGATC

AATCGATCAAGTTTTTTTTTTTAAAAAATGTAAATTATATTAAATCCAAAATAATACAACAATAAACGGG

GCATACCCGCAGTTAGAGAAAATCAAAAGTCACCCTGACATAAAAAGACTTATATCATGCTCCTAAAACA

AATACAAGTGTACTTGTCTATACATAAGAAATTTAATATTGCCAATAACCTCTATTACAGAAAATTAATA

ATTTTCAAAAATTACTTTATTTCTATACAGTATGATGTAGTATAAATATATTGTTGCTCTCAAAAAAATA

AAATTATAAACATCTAACGTGTGGCCGAATGGACTGGTTTATCCATCATGGGATTCAAATCAAATCAAAA

GTCATTGGGTCATGTTGTGTTTAGTTGATTAGAACCCTTTCCAATTTTCAAGCATGTAGCTCGCAAGTCG

CAAGTACCAACTCATGACTGCGACCCAACGTGCACATTACGCTATCATTGAATAAACCAGCTTCCGATAA

AGATACACTACACTTTGCTGATAAACCTCCACTAGAGAGATGCTTCTGTAAATTTAGCATCGGTATTTGA

GTTTATTATATCAGTAAAATACAGAGGCATTGTTAATATATTAATAGGAGTATTGTTAACGTAGATGTAT

TTTAAATATACTTGTGTTTGAAGTTTCACATTAATAATTAAACTTAACTTTTAATATTAATTTTTATTAT

GCAAATAATCATTCAAGTTTAATCAGATATTTTAAATTCGAATCTTAGATATTCATAGCAAAAAAAATTT

CGTCTATAATAATTTTATTCGACTTAAATAAAATTATTTTTGATAAATAAATGATTCATGTGATCTTATA

CAAAAATTATGAAAGCAAAAGTTTAATTATAATAAAAATCTTAAAACGTTGAACACAATAACAAGAGTTA

TAATAATAATAAATTAAGTCACATAAATGATTAATGTGGGCAAGTTAAAAATAAATGTTCAATACTATTT

GAGTTTGATATTTAATAAAAATAAATTTTTATCAGATTTTATTTATAATTAAAATATTATTTTTTAATCA

ACAAGAATATTAAGAAAAAAAATATTATAATATCGGTAGAAGAAAATATTAGTTAGTTTCTATAACCCGA

CGAACCACAATAACTAAATAAACAAATAAATATTGTACCACTGTAAATACCGTACAGAAACTTATGATTC

CGTGTGTGTACTTTTGATCACATTGTGGCTTATGATGTTAGTACTTTTTAGTAGTTAAGCCGAAAGCCGC

AAGCCGCAAGACCCTCCCTGCTTTGAGGGCTGCACGTGACACCCATAACTCTTTTTTTTCTTGTTGCCAT

TCGCTAAGATGATATCCAATTAAAATAAAAATGAGTGACCCAGATAAAAGTTTCCTTCTAGTTTTTTTTT

TAATTATTAGAATGTTAGTTTTGTTTGTTAAAAAAATTAAACTCGTAATATTTTATTTTTTTTAAAACTA

TTCAACCATCTTATATCTGCTGTTTCCTTGCAGTAGTTCATAATAAACCTTAATTAGAAGCAAACCCACT

TTGAAGCCAAAATTTTTGTTGGGTATTTTCTTCTCTTCCCAAAAGGGAAAGTCAGAGTTGAAGGTGATGA

TGCAGATCGTGATCAGAGTCCGTAAAAGCTGCATGTTGCTATTTACGGCTGCAATCATCATCACATGTCC

CTTCCGTATTTTGCCTATTTAGAAACCAATCCACTCTTTACTATTACTAATATGCTATAATGATTGAATA

ATACTAATACTTAAATATAAGTAAGCACCAGAGAGAGAGAGAGAGAGAGAGAGAGAGAGAGTATTAAATT

ATTATTATGGCATGGCAGTGTCTCAGTTCTAACTGCTGAGTTCTGTTCTGTGAGCTTGAAGGGGTTCCAA

CTTCCAAGTTCCAATCCCAAAGGGCGGTGATTAAGATTTTGTGCTTCGTCACAATTCACAACCAGACATA

TATATGCAGATGCAGCTGTAACAGTAAAGTTGCTAGCGGGGATTTTTATTCCCAAGCTACTTCTGCTTCT

AATAAAAACCTCACCCTTCTCTCTGCTCATCAGAATTTTTCCTTGCAAGGTGGGTTTTCCCTTTCTTCAT

TTTCTTTCTCCTTGCTTTAGTTGATTCTTCCTTCTTTTTGGGTAGGTTGGGTTATCACTACCCCTTATTC

TCTTAAATTTGCTTTTCTTAACTTTTCTGATGTATGTACTCTTTTTTGACCACGGAAAATTCTTGTTTAC

CGTACTCTGGATGATGTTTTCTGTAGTTTCCGGGTCAATTAAGGTTCCGTACCAATTACTGTTCTTGACA

ATTAAAGTGGAAAAAATGAAAATCAAATTGCAAAAAAAAAAAAAAAGAAAAAGAAAGAGAAACTATTGTT

TTGCTGAAAAAGTGTATAGTTGAGTGCATGCTCATGAGTTCCTCTGAAGAATTTATAACCTCTTACCATA

TGCATCAGATAACAGTGGAAGATTAATGATAAAGATTCTTAATATTATTATTGTTGTTTGATCTTTGGAT

AGAGTATTACAACAGGATATGATTGGATCATTAATTATCATTACTTACTGAAATCCTTTCAAAGTTTTGT

TCTTCATGTGTGGAAAAATCCACTTTCTCTTCTACAATTGTGACAGTGAGTAAGGATGATGTGAATTGAC

TAGGTCCATACTCTTAGAAGCTTTGCATCTTTACTAGAGCAAACAGTTTCCATTCTTTCTTGTGAGAGAT

TAACTTAGTGATTCTATGCTTGTGGTTTCAAGTGCAGTTGAAGTGACAATGTCCTCTTCAAGGCCCAGCC

AATCATCCAGCAATAATTCTGGCAGATCTAGAACATCAAGACTCAGTGCTAGGAGGATGGCTCAGACAAC

TTTAGATGCAAAACTGCATGCAACTTTTGAGGAATCAGGTAGTTCTTTTGACTACTCCAGTTCAGTGAGA

ATGTCTCCTGCTGGTACTGTCAGTGGAGACCATCAACCAAGGTCTGATAGAGCAACAAGTTCTTACCTCC

ATCAGACACAGAAAATCAAGCTTATCCAGCCATTTGGGTGTTTGTTAGCTTTAGATGAGAAAACATGCAA

GGTCATTGCTTACAGTGAGAATGCACCTGAAATGCTCACCATGGTTAGTCATGCTGTCCCCAGTGTAGGT

GACCACCCTGCTCTTGGCATTGGCACTGACATAAGAACTATTTTCACTGCCCCAAGTTCTGCTGCTATTC

AGAAGGCACTGAGATTTGGGGATGTTTCACTTCATAACCCCATTCTAGTCCATTGCAAGACCTCTGGGAA

GCCCTTTTATGCAATTATCCATCGTGTTACCGGTAGTGTGATCATCGATTTTGAGCCGGTCAAGCCTCAT

GAAGTTCCCATGACTGCATCAGGAGCCCTGCAATCCTACAAGCTTGCAGCAAAAGCAATAACTAGATTGG

AATCCTTGACTACTGGGAACATGGAAACACTATGTAACACAATGGTTCGAGAGGTTTTTGAGCTCACAGG

TTATGACAGAGTGATGGCTTATAAATTCCATGAGGATGATCATGGGGAAGTGATTGCTGAGGTTAAAAGG

CCAGGCCTAGAGCCATATCTGGGGTTGCACTACCCAGCCACTGATATTCCTCAGGCGACACGCTTTTTGT

TTATGAAGAACAAGGTGCGTATGATAGTTGATTGTTGTGCAAAGCATGTGAATGTGCTTCAAGACAAAAA

AATTCCATTTGATTTAACCTTGTGTGGATCAACCTTGAGAGCTGCTCATAGTTGCCACTTGCAATACATG

GAGAACATGAATTCTAGTGCTTCCTTGGTTATGGCAGTTGTGGTAAATGACAATGATGAAGATGGGGATA

GTTCTGATGCTGTTCAACCACAGAAGAGTAAGAGACTCTGGGGTTTAGTAGTTTGCCATCACACTACTCC

CAGATTCGTTCCTTTCCCTCTTAGGTATGCTTGTCAATTTCTGGCTCAAGTATTTGCGGTTCATGTGAGC

AAAGAGCTAGAGATAGAGTATCAGATTATTGAGAAGAACATCCTGCAAACTCAAACACTCTTGTGTGATA

TGCTGGTGCAAGGTGAGCCCCTAGGCATTGTTTCACAAAGTCCTAATATAATGGATCTTGTGAAGTGTGA

TGGAGCAGCCCTGCTATATAAAAACAAGGTGTGGCGATTAGGGGTAACACCAAGTGAATCTCAGATAAAA

GAGATAGCTTTGTGGCTCTTTGAGTGCCATGAGGATTCCACAGGTTTTTGTACAGATAGCTTGTCTGATG

CAGGCTTCCCTGGGGCTGCTGCTCTTGGTGATATTGCATGTGGAATGGCAGCTGCCAGAATAGCTTCCAA

AGATATACTTTTCTGGTTTCGGTCTCACACAGCCTCAGAAATCCGATGGGGTGGTGCAAAGCATGAGCCT

GGTGAAAGGGATGATGGTAGGAGGGTGCATCCAAGATCATCATTCAAGGCTTTCCTTGAAGTTGTGAAGA

CAAGGAGCTTACCCTGGAAGACCTATGAAACGGATGCCATTCATTCGTTGCAGTTAATACTGAGAGATGC

ATTCAAAGAGACACAGAGCATGGAGATAAGCACATATGCTATCGATACAAGGCTAGGTGATTTGAAGATT

GAAGGAATGCAAGAACTGGATGCAGTGACAAGTGAGGTGGTAAGGTTAATTGAAACAGCAACGGTGCCAA

TTTTGGCGGTTGATGTTAATGGGATGATCAATGGATGGAACACAAAAATTGCTGAGTTGACAGGTCTTCC

AGTTGATGAAGCTATTGGAAAGCATTTACTCACACTTGTAGAGGATTTTTCAGTAGATAGAGTCAAGAAG

ATGTTGGACATGGCATTGCAGGGTATGCCTTTTTTCCTTTCCCCGTCTTATTCTTATTCTAGCTCTTATG

TTCTTTTACTTTTTTTTGTTAGGTTTTAGCATTCATAAGCAAACTTTGAAATGAGTGTAATGATATTTAG

ATTTGGGGATGTTATGTTCTCTACAACCATCCATAAATATTTCATTAATAAAGAATAACAGCTTCCAAGT

TTTATTCAATTTTAAAAGACTTAGGAAGGAATGGAGCATTGCAAAAGATACTTTATCCTAGCTTTCCATA

TTTCTACTTAATTGATTTGTTATGCTACAATATGATTAGGTGAGGAAGAGAGAAATGTCCAATTTGAGAT

CCAAACACATCATATGAAGATTGATTCTGGTCCCATCAGCTTGGTAGTTAATGCTTGTGCAAGCAGGGAT

CTTCAAGATAATGTTGTGGGAGTTTGTTTTCTGGCACAAGATATAACTGCTCAGAAAACAATGATGGACA

AATTCACCCGAATTGAAGGTGACTACAAGGCAATTGTACAGAACCCAAACCCATTGATCCCTCCAATATT

TGGCACAGATGAATTTGGTTGGTGTTGTGAATGGAATTCAGCTATGGCAAAATTAACTGGATGGAAGCGA

GAGGAGGTAATGGATAAAATGCTTTTAGGAGAGGTTTTCGGGACCCAAATAGCTTGTTGTCGCCTAAGGA

ATCATGAAGCTGTTGTTAACTTTAGCATTGTACTTAATACAGCCATGGCTGGTTTGGAAACAGAGAAGGT

TCCTTTTGGTTTCTTTGCTCGTGATGGAAAGCATGTAGAATGTATTCTTTCTATGACTAAGAAATTGGAT

GCAGAAGGTGTAGTTACTGGTGTCTTCTGCTTCTTGCAACTAGCAAGTGCAGAGCTGCAACAAGCATTAC

ACATTCAGCGCATATCTGAACAAACTTCATTGAAAAGACTGAAAGATTTAACTTATTTGAAAAGGCAAAT

CCAGAATCCTTTATATGGGATTATGTTCTCCCGGAAATTGTTAGAGGGTACTGAGTTGGGAGCTGAACAA

AAACAATTTCTGCAAACGGGCATTCGGTGTCAACGCCAGATTAGCAAAATTCTGGATGACTCGGATCTTG

ACAGCATCATTGATGGGTATGATATCTGTGAATGTGTTGCTTCTAGTTTTTATTTTTACTTGCCATTTGT

GTGGTATTTTGAGCATTTATCAACAAGAGTTCCTATTAAATCATACAATTTCGTATTTCATAACCACACC

TAATTTATGCTTCTAGTCTTTTGTCCTTGTCATTTGTGTGATGTTAAATCATTTATCAACAATAGCCCCT

ATTAAATCAACCACACTTAATATTGAGTGTCAAATAGTTTATAGTCAAACATGAAAGAGATCAGGATTTG

AACTGTGTTCTGTTTTGATAAGTGAATACTGACTCCTGAATTTCAGGAATCAGATTCTTTACAACTACTT

TTTTTTTTCCAGTAAAGACCTTGATGTACTAGTGGCCCTTTTGTCCTGCATAACAGCTACATGGATTTGG

AGATGGTTGAATTCACTTTGCATGAAGTTTTGGTTGCCTCCCTAAGTCAAGTCATGACAAAGAGTAATGC

AAAAGGTATCCGAGTAGTCAATGATGTTGAAGAGAAGATCACAACAGAGACCTTATATGGTGATAGTATC

AGGCTTCAGCAGGTCTTAGCTGACTTTTTATTGATTTCCATCAATTTCACACCAACTGGAGGTCAGGTTG

TTGTAGCAGCCACGCTAACCCAACAGCAGTTAGGGAAATTAGTTCATCTTGCTAATTTGGAGTTCAGGTA

ATCAACTATTCCAGACTCAGATGGACATTTCAAACTTTCTTGTGGGTCTTCAGTTCAGTTGGTTTAAATG

CCTGCAAAATAAACGTGACATGAAAAGAAACATATTATCACTTTTTTATGAGTTTAGTTTAACAGTATAA

ATGTTTTTACAGTCAACTAACTAAAAATTACCTTGTAATTGGAGGGTATTGGATGATGCTTTGTCAAAGT

TAATGAAGCTTTGTAATTGGATGACAGTGTAAAACTGTTGTACATTGTCTGTGCATAAACTATTTTCTCT

TTTTACATTATACACTCTTGTATGAGAACTAAAGGCTCTCACATTGGTCAAATAACAACAACAAACAACA

ACGCCTTATCCCACTAGGTGGGGTCGACTACATGGATCAACTTCCGCCATAATGTTCTATCAAGTACCAT

ACTTCTAACCAAACCATTAATTTCGAGATCTTTTTTGATAACCTCTCTTATAGTCTTTTTGGGTCTTCCT

CTGCCTCGAATTGTTTGTCTTCTCTCCATCTGGTCTACTCTCCTCACTACAGAGTCTACCGGTCTTCTCT

CTACATGCCCAAACCACCTAAGTCTATTTTCCACCATCTTCTCTACAATAGGCGCTACTCCAACCCTCTC

TCTAATAGCTTCGTTTCTAATTTTATCCTGTCGAGTCTTACCACACATCCACCGCAACATCCTCATCTCC

GCTACACCTACTTTATTCTCATGTTGGCTCTTGACCGCCCAACATTCTGTTCCGTACAAAATCGCCGGTC

TTACCGCAGTCCGATAAAACTTTCCCTTTAGCTTGATCGGTACCTTTGCATCACATAACACCCCCGATGC

TTTTCTCCATTTCATTCATCCTGCTTGAATGCGATGATTCACATCCCCTTCAATTTCCCCATCATCCTGT

ATTACAGACCCAAGATATTTAAACCGTGTGACTTGAGGAATAATATGGTCTCCTATTTTCACCTCTGAGT

TAGAAACCCTCCTTCTTTTGTTGAACTTACATTCCATATACTCCGATTTGCTTCTGCTTAGGCGAAAGCC

ATGTGTTTCTAGAGCTCGTCTCCAAGTTTCCAACCTCTCATTCAACTCCTCCCTCGACTCTCCAAGGAGG

ACTATGTCATCTGCAAAAAGCATGCATCTCGGCGCTATCTCTTAGATTTGTTCCGTGAGGACATCCAGAA

TTAAGGTAAAAAGGTAGGGGCTAAGGGTTGACCCTTGATGTAAACCAATTGTGATGGGAAAATCGTCTGA

CTCTCCACCCTGTGTCCTAACACTAGTCGATACCCTATCATACATATCTTGGATAGCTCGAATATATGCA

ACCCTAACCCCTTTCTTCTCTAGAGCTTTCCACAAAATCTCTCTAGGCACTCTATCATACGCTTTCTCCA

AGTCAATAAAAATCAAGTGCAAGTCTTGTTGGGCCATGCGATATTGCTCCATCACCTGCCGTAATAAATA

AATCGCTTCCATGGTCGACCTTCCCGACATGAAACCAAATTGATTCTCAGTAACTTGAGTCTCCTTTCTT

AATCTCCGTTCGATCACTCTTTTCCATAATTTCATGGTATGACTCATGAGCTTGATTCCCCTATAATTTG

CACAATTTTGTATATCCCCCTTGTTCTTATAGATTGGCACTAACGTGCTTCTCCTCCATTCCTCCGGCAT

GCGTTTTGACCTCATAATTTCGTTAAAGAGTTCGGTGAGCCACTCAAGACCTCTATCTCCAAGAGTTTTC

CACACTTCAATAGGTATGTTGTCTGGCCCCACCGCCTTACCATTACTCATTCTTTTCAACGCTTCCTTTA

CTTCCTGTTTTTGAATCCGACGATAGTACTTATAGTTCCGGTCCTCTTCTCTTGTGTCTAGACTGCTAGA

GTCATATCCATATCCATCATTAAATAAGTTGTGGAAATACGCCTTCCACCTTTCCTTGATATCTTTTTCA

TGCACTAAGACTTTGCCTTCTTCATCCTTAACACACTTTACTTGATCCAAATCTCTAGTCTTCCTCTCTC

TACCCTTAGCAAGCCTATATATAGATCTTTCTCCGTCCCTGGTTCCTAGAGCTTGGTATAGTCCGTCAAA

AGCTTGGGCTCTTGCCTCACTCACCGCCTTTTTGGTTTCATTTCTAGCTATCTTATACTTATCCCAAGTT

TCAGAATTTCTACACCTAGACCACTCCTTGAAACACTCCTTTTTTACTCTAACTTTGCTCTGAACATTTT

CATTCCACCACCACGATTCTTTACCCCTAGGTCCAAAACCTCTAGATTCACCCAACGTCTCTTTAGCCAC

TTTAATAATCTCTTGGGACATCTTGTTCCACATATCATTTGCACTTCCTTGTGATTGTCCACACCAACCC

TCCCATATCTTTTGTTGGAAGATTCCTTGTTTCTCACCCTTCAAGTGCCACCATTTGATCCTTGGTGCTA

CCAGAGGACTTCTTCTCTTTGCCCTATCTCTAATTCTTACATCCATAACCAAAACTCTATGTTGGGTAGT

CAAGCTCTCTCCCGGGATAACTTTACAGTTCAAGCAATACTTCCTATCAGACTTCCTGATAAGGAAGAAA

TCTATCTGAGAACATGTCCCTCCACTTTTGTAAGTGATAAGATGTTCCTCTCTTTTCTTAAACCATGTAT

TGGCTATAGAAAGATCCAAAGCCTTCGAAAACTCCAAGATGGATTTACCCTCCCCATTCATCTCCCCTAG

GCCAAAACCCCTATGCACCCCCTCAAAACCTCTAGCCACGCTACCTACATGTCCATTGAGATCCCCTCCT

AGGAAAACTTTCTCTCCTTGGGGTATATCCTGAAGTACCCCTTCTAGATCCTCCCAAAATTTTACCTTAA

AGTGTTCTGCTAACCCAACCTGAGGTGCGTACCCACTAATAACATTAAAGGTGTCCTGTCCCACTACCAA

TTTTAAGACTAATAACATTGGTCAAAACGACAAAAAAAAATGTGTGTATGGAGGGGTTGAGAGCAATTGG

TTTGAATCTTTTGGATGATACCTTCCTATGCTTGATACACGCCGTTTTGAGATTATATACGTGATTTCTA

AGTCATTGTGTTTGCTTTTGTTATTTTTTGCAGCATAACGCATGATAGTTTTGGGGTTCCAGAAACATTG

CTGAACCAGATGTTTGGACGCGATGGACATGAATCTGAGGAGGGTATTAGCATGCTGATTAGCAGAAAGC

TGCTAAAGCTCATGAATGGAGACGTACGTTATTTAAGGGAAGCAGGCAAATCATCTTTCATCCTATCTGT

TGAACTTGCCGCAGCACATAAATCCAACACTTAACATTTTGGAAATAAAACCAAACCATATTTTTTGTAC

ATCAGAAAGTGAAAGGAAGCAAGAAATTTGCATTCCTTGTTCAATATTTAACTCTTGTTATTGATTACCT

TCTTGTAAGGTGATGTAGATATGTAATGTATAAGTGTATAACTAACTATTTACACACTATAGACCTGTTC

TCATCTACAGCAATGATGCTTCATTTGCAACAAAATGAAACATAGGGACTAACTAGGAGCATGTTAAATG

GCATTGTTTTCTACCTTTATTTCATTTTCTTCCAAAACTTTATTTAAAACATCCTGCTTAGAGATACCAT

AAGTATTAAATATTAGTAAAGTATTTCTTATAAATACTTTTAATTACTTCAAAAATAAATATTAAATATT

TTTTTTAAGTAATAAATAATTTTTGTTATCTTCATTTTTTCATCTGTCAGTGTCACTTTAAAAATAGTTA

TTTATTTGTCATTTTAAAATTCTAAAATGACATTAGTTACTTTTTTTTAAAATTATATACTTATAAAAAA

AGAGAGAGACATAATAATATATGAATGCCAAAAAATAAAATAAATAAGAGAGGGAAAAACATATTAAAAA

ATGATAAATAATTTTAATAAAAAGTAGGAATTTATTAACTTTTCTCAATTTATGTTCAATAATAAATAAA

AAGAAACAATAATATTTTTAGGGCATAAACATGTATATGTGATCTTAAATAGTAAATAATTTAAAATGGA

AGGCTTGCAGTTTTATGTGGATAGTCATTTGTTTTCATAATTTGTTGTGATGATGTTTATCTAATTATTA

CTGAAACCAGAGGCGGGACTTAGA

>AB643561.1 Glycine max PhyA1 gene for Phytochrome A1, complete cds, haplotype: 13

ATGTCTACCTCAAGGCCTAGCCAATCATCCAGCAATTCAAGGAGATCAAGACATAGTGCTAGAATGGCTC

AGGCAACTGTAGATGCAAAAATCCATGCAACTTTTGAGGAGTCCGGTAGTTCCTTTGATTACTCCAGTTC

GGTGCGTGTCTCTGGTACAGCTGATGGAGTCAATCAACCAAGGTCTGACAAAGTTACAACAGCTTACCTC

AATCACATGCAGAGAGGCAAGATGATTCAGCCTTTCGGTTGCTTGTTGGCCATTGATGAGAAAACATGTA

AGGTCATTGCATACAGTGAGAACGCGCCCGAAATGCTGACCATGGTTAGCCATGCTGTCCCCAGTGTTGG

TGACCACCCTGCCCTTGGCATTGGCACTGACATAAAAACTCTATTCACTGCACCAAGTGTTTCTGGATTG

CAGAAGGCTCTAGGATGTGCGGACGTTTCGCTTCTTAACCCCATACTTGTCCATTGCAAGACCTCTGGGA

AGCCCTTTTATGCAATTGTCCATCGCGTCACTGGTAGTTTGATCGTTGACTTTGAGCCAGTCAAGCCTTA

TGAAGTTCCCATGACTGCAGCAGGTGCCTTGCAATCTTACAAGCTTGCTGCCAAAGCAATTACCCGATTG

CAATCATTGCCTAGTGGGAACATGGAAAGACTATGTGATACTATGGTTCAGGAAGTTTTTGAACTCACAG

GTTATGATAGGGTGATGGCTTATAAATTTCATGAGGATGATCATGGAGAGGTGATTGCTGAGATAACAAA

GCCCGGTCTTGAGCCATATCTGGGTTTGCACTATCCAGCCACCGACATTCCCCAGGCTTCACGCTTTTTA

TTTAGGAAGAACAAGGTTCGTATGATAGTTGACTGTCATGCAAAACACGTGAGGGTTCTTCAAGATGAAA

AACTCCAATTTGATTTGATTTTGTGTGGTTCCACCTTAAGAGCTCCTCATAGTTGCCACGCGCAGTACAT

GGCTAACATGGATTCAATTGCTTCCCTGGTTTTGGCAGTTGTAGTCAATGACAACGAAGAAGATGGGGAC

ACTGATGCTGTTCAGCCACAAAAGAGGGAGAGACTTTGGGGTTTGGTAGTTTGCCATAACACTACTCCCA

GGTTTGTTCCCTTTCCTCTAAGGTATGCTTGTGAATTTCTGGCTCAAGTATTTGCCGTCCATGTGCACAA

AGAAATAGAGTTAGAATATCAGATTATTGAGAAGAATATCCTGCGCACCCAGGCACTCTTGTGTATGCTG

ATGCGAGATGCACCCCTAGGAATTGTATCAGAGAGTCCTAATATAATGGATCTAGTTAAATGTGATGGAG

CTGCCCTCATATACAGGAACAAAGTATGGAGATTAGGAGTGACACCAAGTGAACCCCAGATAAGAGAGAT

AGCTTTGTGGTTGTCTGAGTACCATATGGATTCCACAGGCTTTAGTACAGATAGCTTGTTTGATGCAGGG

TTCCCATCGGCTCTTTCTCTGGGTGATGTTGTGTGTGGAATGGCATCTGTTAGAGTAACTGCAAAAGACA

TGGTATTTTGGTTTCGGTCACACACTGCTGCAGAAATCCGATGGGGTGGTGCAAAGCATGAGGCTGGAGA

AAAAGATGATAGTAGGAGGATGCATCCAAGATCATCATTCAAGGCTTTCCTTGAAGTTGTGAAGGCAAGG

AGTTTACCTTGGAAGGAATATGAAATGGATGCTATTCATTCCTTGCAGATAATACTGAGAAATGCATTCA

AAGAAGATACCGAGAGTTTGGATTTAAACGCAAAAGCAATTAATACAAGACTAAGTGATTTGAAGATTGA

AGGGATAAACGATTTGAAGATTGAAAGGATGCAGGAACTGGAAGCAGTGACAAGTGAGATCGTTAGGTTG

ATTGACACAGCAACAGTGCCTATTTTGGCCGTTGATGTTGATGGGCTGGTCAATGGGTGGAACATAAAAA

TTGCTGAGTTGACGGGTCTTCCAATTGGTGAAGCTACTGGAAAGCATTTACTCACACTTGTTGAGGATTC

TTCAACTGATAGAGTCAAGAAGATGCTTAACTTAGCACTGCTAGGTATGTCTTTTTTTCTTTCTCTATCT

TATCCTTCTATACTCTTTTCTTTTCTTTGTTTTTTCAAGCCTTATCATTCATAAGCAAAAGCTTAAAAAA

TGAGTTAGTATGTGCCTGTGTCTATCTAGCTGTGAGCAACTGAGCATATTATGTTCCCTAAAGCCACACA

TGAACATTGCATTGGTGAAGAATTCTGAGAAGAAAAGTATGTCTTGTTGACAGTTTCATTCAATTTTGTG

CCCTTACAATAACTTGGGAAGGAATGGAACACCCTAGGAGTTGTGATGTCATTTTTATATTGGGGGCTTG

GTGAGAGGAATTGATGCAATTTATCTGACACAGTGGGCTTGTATTCTTCTAACCTGCTTTCCATATTTCT

TAATTGTTGTTTTGTTCTGTTAATTAGGTGAAGAAGAGAAGAATGTCCAATTTGAGATCAAAACACATGG

GTCTAAGATGGATTCTGGTCCTATTAGTTTGGTAGTAAATGCTTGCGCGAGCAGGGATCTTCGAGATAAT

GTTGTCGGGGTTTGTTTTGTGGCCCATGATATAACTGCTCAGAAGAATGTCATGGACAAATTCATCCGTA

TTGAAGGTGATTACAAGGCAATTGTACAGAACCGCAATCCATTAATCCCTCCTATATTTGGCACAGATGA

ATTTGGCTGGTGTTGTGAGTGGAATCCAGCTATGATGAAGTTAACTGGATGGAAGCGAGAGGAGGTGATG

GATAAAATGCTTTTGGGAGAGATTTTTGGCACCCAGATGGCTGCTTGTCGCCTAAAGAATCAAGAAGCTT

TTGTTAATTTGGGCGTCGTACTTAATAAAGCCATGACTGGTTCAGAAACAGAGAAGGTTCCTTTTGGTTT

CTTTGCTCGGAATGGCAAGTATGTAGAATGCCTGCTTTCTGTGAGTAAGAAATTGGACGTAGAGGGCCTA

GTTACTGGGGTCTTCTGCTTCTTACAGCTAGCTAGCCCAGAGCTCCAACAAGCATTACATATTCAGCGTC

TATCTGAGCAAACTGCTTCGAAGAGATTGAATGCATTAAGTTACATGAAAAGGCAGATCAGGAATCCTTT

GTGTGGAATTGTATTTTCCCGGAAAATGTTGGAGGGTACTGACTTGGGAACAGAACAGAAACAACTTCTG

CGCACTAGTGCTCAGTGCCAGCAGCAGCTTAGTAAAATTCTTGATGACTCAGATCTTGACACCATCATAG

ATGGGTATGATATCTGTGCATTTTGTTCTTTTTCCCCCTTATTTACATTCATGTGGTGTGAAGCATTTAT

CAGTTGGAACTCCTACTAAAGCATAAAAGTTCCTATTTGAAACCTCATTGAAACTCATGACTAGAAAGTT

AACCTCATTCATGCAACTAAACTCCAAAAACATTGACTAGTATATAACCCAACTGATATTGGGTGTTTAC

TGTTATCATGTTCTGTTTTTTTTTTTTTGCTCAGCAAAAACTGTTATCATGTTATGTAGTTCTCAAGGTT

CTTGTTATGTAGTTTTGTCATGTTTTGTACTTTTGTTAGAGTTTAGTGTACCTATTTAGAAGTTTAAATA

TTAGCATCTTTGAAAGAAGGCATCCAATGTGGATGTTTATTTTTCATTCACACGGTGCTTTCAAAGTCAC

TCTGTAGTGCTAGATTAATTTGTGTAAAAATACAGTTTCCTCATTTATTTGTCTTGGAATCTTCGATAAC

AGTTTTATAATCATTTTTTCCCCCTGTATGTTCAACAGTTACTTGGATCTTGAGATGGCTGAGTTCACCC

TGCATGAAGTTCTGGTTACCTCCCTTAGTCAAGTCATGACAAAAAGTAATGGAAAGAGTATCCGAATAGT

CAATGATGTTGCAGGGCATATTATGATGGAAACTTTATATGGTGATAGTCTTAGGCTTCAGCAGGTCTTG

GCTGACTTTTTACTTATTTCCATCAATTTCACTCCAAATGGAGGTCAGGTTGTTGTAGCAGGCTCTCTAA

CCAAAGAACAGTTAGGCAAATCTGTCCATCTTGTTAAGTTAGAGCTCAGGTAACTCTTCCATGCTCGCAT

TCCCACTGTGTATGTGATTTAAATGCCTGGCGGAAACACTGCCATAAATTGTTGCATGATCAACACATTG

TTCTCTTGTGCTAGGCAAAGAAACTTGCATTGTTTATGCGGTCTTGCTAACCATTAAGGACACTGATTAA

GGAATCAACAAGTGGTTGAGATTATACGTTTTCTAATAAAAAACTTTTCACTTTTGATTCCTTAACCAGT

GTCCTGATTAACATTTTCCTTGTTTTCAACCAAAACATAAAGAAGGAAGTTACACTATAACCTTGATTTA

CTTCTAAGTGAGTGTTACTTTTGGTATTTATGTAGCATAACACATGGTGGGAGTGGGGTACCAGAAGTGT

TACTGAATCAGATGTTCGGAAACAATGGGCTAGAATCCGAGGAGGGTATTAGCCTACTGATAAGCAGAAA

GCTGCTGAAGCTGATGAATGGAGATGTTCGCTATCTAAGGGAAGCAGGCAAATCAGCTTTTATCCTCTCT

GCTGAACTTGCTGCAGCCCATAATTTGAAAGCTTAAAAGTTTTGGAAAAAAGAATAAAAGATGATGCAAC

AGCAACACAATCTGTTTTCTACATCATAAAGAGAACGGAGCTAGAAATTAATTTGCATCCAATGTAACTC

TAATTTGTTCCCACGTTTCTTTAACTGCTTCTTGTAACGTACGTTGTAGATATGTATGATTAATCTTCTT

CCCATTCCTTGACCTGTTCTCATTCTCACTAGAGCTTTTCCTTATTTATTCCTTCAGCAAACAGCAATGA

TGAGGCTAATTCTGTCTCTCGCTTCTTATCTTCACGTGTTAGCCATGGTTTTCTACCTAACTTTTTTTCT

TTTTGATTACCACCTAACTTTTTTTTTCCTGCTTGCAACGGTATATAAGTTGTCTTCAATTTATTGATCT

GCACATTTATTGCAGGTTGTTTGTATTCGTGGATTTGTAAACACGGTTTTCTTTTCTCTGCTAGCTGTGT

TTTTGTACGACTAATGATTCAAATTCGATCAAATGACCAAAAGAAATCTTAAACCTTAGACTTTTCTCAT

TCTTCATATGTTATAGGCGTGTCTGTCGTAATTAGGATTTTTATAAATATAATAAAATTTAGAAAAGTAC

TAAATCTTAGAGTATTTTAAATGAAAAAAATTTATCACTTTTGTGGATCCTATAATATCACAGGATACAA

GAATTTTTATCATTTTTACTTTAATAGTAGAACTTAATTTGGGTTATTAGATTTTATTTGCATATAAAAA

ACCAATTTTTTCTCTCTCAATGACATTAAAAATAATATTTTTTTAATAAAAGTGGAATTCTTAAAAGTTT

TTCCTGTAATTCAACCCCAGCCAGAACTCTTGAGCAGAACAAGAATAAATCTAG

>AB643571.1 Glycine soja PhyA2 gene for Phytochrome A2, complete cds, haplotype: 13

TTAAAAACTAGTTGAATAATTGTAAACTTATGATTGCTTATAATTTAGACTTGAACAACAAGATGAAATT

TTGCTACTAATAGTCCTTACATGTACTTAACATCCTATCCAAACCAATTACAGAGGAGTGTCAACTAGTT

TTACACATTTTTACATTAAAAAGAAAATAAAACAAAAACTTTAGTTAAAAAAATGTTGATTTTGGTATAT

TGGCCACTCATAAAAGCTTGAACTGTAGAACGAATAATGATGAATTGTTTAATATTTTTTTTTTATTTTA

ATAATCAGAGTTCATTCCCCATTAACTCCCACATTCACATTCACATGGTGCTCGAAGTGGGTACTATAAA

AATGATATCTTTATGGCATGGCGGTCCCTGGTCACACTCACAGTTGTGGTGAGTTTGAAGAAGAGGGGGT

TGGGTTCCTTGGAGAGACATGGGTGTTGTTGTTGCTCACACCTCTTTTCTTTATTTTAACCCTTAGCTTC

ATTGCTTCCTTCCTTTTGTTGCCACTCTTTCCCTTTCAAGGTTCGTTGCTACCATACTCACCTTTTTTCC

TTTTTTCTCTTCCCTCCTCTGCTGTCTGTGATGTTATCTTATCCTATTCACTTTCCTGCTTTTAATGTTT

GTGATTCGTGTCTGTTTGTTCCTGGCAAACTATGTTCTTGTTTCACTGGAAATTGTTTTGTCGGCTCGTA

GTTGTTCAGTGTGTTAACGTACAGTATGATCAATTTTCTTCAAAATTGAGAATAATAACGTTCAATTTTA

TTCCATCATTGAGCATGTACAGTTGTGTGATTGGGTCGTTGTTCACAATCTACATGATGTGTATTTATTC

ACCTGAGCTGTGGAGATTGTTTGTATTTCATGATTGGGTCATAAAATAAATTGTTAGTAGTTACTCAAGT

CTTTTGTAATTTTGTCATCTTGAGAAGGTGTTGGAAGTTTGTTATTATCTTCTCGAATTGTTACATGAGC

AGGAGGGAATTGATTGGCATTGGAGTAAATTGATTAATAGATTTTAACTTGTTCTCAGATGTTCTTATAT

GATTCAGATTAACTTTTACTCATTATTTTTTACATTTCAAGTGCAGTTTGGGGCGAAAATGTCTACCTCA

AGGCCTAGCCAATCATCCAGCAATTCGGGGAGATCAAGACGTAGTGCTAGGGCTATGGCTCTGGCAACTG

TAGATGCAAAACTCCATGCAACTTTTGAGGAGTCTGGTAGTTCCTTTGACTACTCCAGTTCGGTGCGCAT

CTCTGGTACAGCTGATGGAGTCAATCAACCAAGGCATGACAAAGTTACAACAGCTTACCTCCACCACATG

CAGAAAGGCAAGATGATTCAGCCTTTTGGGTGCTTGTTGGCCTTAGATGAGAAAACATGCAAGGTCATTG

CATACAGTGAGAACGCACCCGAAATGCTGACCATGGTGAGCCATGCTGTCCCCAGTGTTGGTGACCACCC

TGCCCTTGGTATTGGCACTGACATAAAAACTCTATTCACTGCACCAAGTGCTTCTGCATTGCAGAAGGCT

CTAGGATTTGCGGAGGTTTCGCTTCTTAACCCCGTCCTTATCCATTGCAAGACCTCTGGGAAGCCCTTTT

ATGCGATTATCCATCGTGTCACTGGTAGTATGATCATTGACTTTGAGCCAGTCAAGCCGTATGAAGTTCC

CATGACTGCAGCAGGTGCCTTGCAATCTTACAAGCTTGCTGCCAAAGCAATTACCCGATTGCAATCGTTG

CCTAGTGGGAGCATGGAAAGACTATGTGATACTATGGTTCAAGAAGTTTTTGAACTCACAGGTTATGATA

GGGTGATGGCTTATAAATTTCATGAGGATGATCATGGAGAGGTGATTGCTGAGATAACAAAGCCAGGTCT

TGAGCCATATCTGGGTTTGCACTATCCAGCCACCGATATTCCCCAGGCTTCACGCTTTTTATTTATGAAG

AACAAGGTTCGTATGATAGTTGATTGTCATGCAAAACACGTGAGGGTTCTTCAAGATGAAAAACTCCCAT

TTGATTTGACTTTGTGTGGTTCCACCTTAAGAGCTCCTCATAGCTGCCACGCACAGTACATGGCGAACAT

GGATTCAATTGCTTCCCTGGTTATGGCAGTTGTAGTCAATGACAATGAAGAAGATGGGGACACTGATGCT

ATTCAGCCACAAAAGAGGAAGAGACTTTGGGGATTGGTAGTTTGCCATAACACTACTCCCAGGTTTGTTC

CCTTTCCTCTAAGGTATGCTTGTGAGTTTCTGGCTCAAGTATTTGCCATCCATGTTAACAAAGAAATAGA

GTTGGAATATCAGATTATTGAGAAGAATATCCTGCGCACCCAGACACTCTTGTGTGATCTGGTGATGCGA

GATGCACCCCTGGGAATTGTGTCAGAGAGCCCCAATATAATGGATCTTGTGAAATGTGATGGAGCTGCCC

TCATATATAAGAACAAGGTATGGAGATTAGGAGTAACACCAAGTGAATCCCAGATAAGAGAGATAGCTTT

TTGGTTGTCTGAGTACCATATGGATTCCACAGGCTTCAGTACAGATAGCTTGTCTGATGCAGGGTTCCCA

TCGGCTCTTTCTCTGGGTGATGTTGTTTGTGGAATGGCAGCAGTTAGAGTAACTGCAAAAGACGTAGTAT

TTTGGTTTCGGTCACACACTGCTGCAGAAATCCGATGGGGTGGTGCAAAGCATGAAGCTGGAGAAAAAGA

TGATGGTAGGAGGATGCATCCAAGATCATCATTCAAGGTTTTCCTTGATGTTGTGAAGGCAAGGAGCTTA

CCATGGAAGGAATATGAAATAGATGCTATGCATTCCTTGCAGTTAATACTGAGAAATGCATTCAAAGATA

CCGAGAGTATGGATTTAAACACAAAAGCAATTAATACAAGACTAAGCGATTTGAAGATTGAGGGGATGCA

GGAACTGGAAGCAGTGACAAGTGAGATTGTTAGGTTGATTGAAACAGCAACAGTGCCTATTTTGGCAGTT

GATGTTGATGGGCTGGTCAATGGGTGGAACATAAAAATTGCTGAGTTGACAGGTCTTCCAGTTGGTGAAG

CTATGGGAAAGCATTTACTCACACTTGTTGAGGATTCTTCAACTGATAGAGTCAAGAAGATGCTTAACTT

AGCACTGCTAGGTATGTCTTTTTTTTCCCTCTCTCTGTCTTATCCTTCTATACTCTTTTCTTCTCTTTGT

TTTTCAAGCCTTATCATTCATAAGCAAAAGCTTAAAAAATGAGTTAGTATGTGCCTTTGTCTATTTAGCT

GTGAGCAACTGAGCATATTATGTTCCCTAAAGCCACACATGAACTTTGCATTGATGAAGAATTCTGAGAA

GAAAAGTATGTCTTATTGACAGTTTCATTCAATTTTGTGCCCTTACAATAACTTGGGAAGGAATGGAACA

CCCTAGGAGTTGTGTTGTTATTTTTATTTTGGGGGCTTGGTGGAAGGAATTGATGCAATTTATCTGACAC

AATGAGCTTGTATTCTTCTAACCTGCTTTCCATATTTTTTATTTGTTGTTTTGTTCTGTTAACTAGGTGA

AGAAGAGAAGAATGTCCAATTTGAGATCAAAACACATGGGTCTAAGATGGATTCTGGTCCTATTAGTTTA

GTAGTAAATGCTTGCGCAAGCAGGGATCTTCGAGATAATGTTGTTGGGGTTTGTTTTGTGGCCCATGATA

TAACTGCTCAGAAGAATGTCATGGACAAATTTACGCGTATTGAAGGTGATTACAAGGCAATTGTACAGAA

CCGCAATCCATTAATCCCTCCTATATTTGGCACAGATGAATTTGGCTGGTGTTGTGAGTGGAATCCAGCT

ATGACGAAGTTAACTGGATGGAAGCGAGAGGAGGTGATGGATAAAATGCTTTTGGGAGAGCTTTTTGGCA

CCCATATGGCTGCTTGTCGCCTAAAGAATCAAGAAGCTTTTGTTAATTTGGGTGTTGTACTTAATAAAGC

CATGACTGGTTTGGAAACAGAGAAGGTTCCTTTTGGTTTCTTTGCTCGGAATGGCAAGTATGTAGAATGC

CTACTTTCTGTGAGTAAGAAATTGGACGTAGAGGGCCTAGTTACTGGGGTCTTCTGCTTCTTACAGCTAG

CTAGCCCAGAGCTCCAACAAGCATTACATATTCAGCGCCTATCCGAGCAAACTGCCTTGAAGAGATTAAA

TGCATTAAGTTACATGAAAAGGCAAATCAGGAATCCTTTGTGTGGAATTATATTTTCCCGGAAAATGTTG

GAGGGTACTGCCTTGGGAACAGAGCAGAAACAACTTCTACGCACTAGTGCTCAGTGCCAGCAGCAGCTTA

GTAAAATTCTTGATGACTCAGATCTTGATAGTATCATAGATGGGTATGATATCTCTGAATTTGTTCTTTT

TCCCCTTATTTACATTCATGTGGTGTGAAGCATTTATCAGTTGAAACCTCATTGAAACTCAAGACTAGAA

AGTTAACCTCATTCATGCAACTAAACTCCAAAAACATTGACTAGTATAACACAACTGATATTTTGTGTTT

GTTGTTATCATGTTCTGTCGTTCTCAAGGTTCTTGTTATGTAGTTTTGTCATGTTTTGAACTTTTGTTAG

AGTGTAGGGTACCCATATAAAATGTTCAATTATTAGCATCTTTGAAAGAAGGCATCCAATAGGTATGTTT

ATTTTTCATTCACACTGTGCTTTCAAAATCACTCTGTAGTGCTAGATTAATTTGTTTGTATGAAATTATG

AATACACAAACATGACCAAAAATACAAAGTTTCCTCATTTATTTGTCTTGGCATCTTCCATAACAATTAT

GTAATCTTTTTTTGTTTTCCTGTGTGTTCAACAGTTACTTGGATCTTGAAATGGCTGAGTTCACTCTGCA

TGAAGTTCTGGTTACCTCACTTAGTCAGGTCATGACAAAAAGTAATGGAAAGAGTATCCGAATAGTCAAT

GATGTTGCAGAGCAGATTGTGATGGAAACTTTATATGGTGATAGTCTTAGGCTTCAGCAGGTATTGGCTG

ACTTTTTACTTATTTCCATCAATTTCACTCCAAATGGAGGTCAGGTTGTTGTAGCAGGCACTCTAACCAA

AGAACAGTTAGGCAAATCCGTCCATCTCGTTAAGTTAGAGCTCAGGTAACTCTTCCATGCCCGAATTCAA

AATTCATTTATTTAATTTAAGTTAGAGCTCAGGCTATTAGTATTCCCACTTTATATGTGATTTAAATGCC

AGGAGGAAACAATGTGTGACATGAATTGATGCATGATCAACACATTGCTCGCTTGTGCTATGCAAAGAAA

CTTGCATTGTTTATGCAGTCTTGCTAACCAGTGTCCTTCCTAAGGACACCGATAAGGGTCGACATTAACA

TTTTCCTTTGTTTTTAACCAAAACATAAAGAAGGAACTTGCACTATAACCTTGATTTACATCTAAATAAG

TGTTTATTTTTTGTATTTATGTAGCATAACACATGGTGGGAGTGGGGTACCAGAAGCATTACTGAACCAG

ATGTTTGGAAACAACGGGCTAGAATCCGAGGAGGGTATTAGCCTACTGATCAGCAGAAAGCTGCTGAAGC

TGATGAATGGAGATGTTCGCTATCTAAGGGAAGCAGGCAAATCAGCTTTTATCCTCTCTGCTGAACTTGC

AGCAGCCCATAATTTGAAAGGTTAAAAGTTTTGGAAAAAAGAATAAAAGATGATGCAACCAGCAAAACAC

AATCTGTTTTGTACATCATAAAGAGAACGGAGCTAGAAATTAATTGCATCCAATGTAACTCTAATTTGTT

CCCACGTTTCTTGTTAACTGCTTCTTGTAACGTACGTTGTAGATATGTATTAATCTTCTTCCCATTCCTT

GGCCTGTTCTTTCTAGAGTTTTTCCTTATTTACTTATTCCTCCATCAAACAGCAATGATGAATCATTTGT

TGCGAAATGAAACGTAGAAATTAGAAACCGCTGATTCTGTCTCTCGCTTCTTATTTCCACGTGTTAGCCT

TGGTTTTCTACCTAACTTTTATGATTCAAATTCGATCAATGACCAAGCGAAATCTTACACTTTTCTCATT

CTTCATATGTAATGTCTGTCGTAATTAAGATTTTTACAATATAACAAAATTTAGAAAAATACTAAATATT

ACAAACTTGCAAATGAATAAAATAAAACTTACTCGAAATTGCGTTAGTAGTGTAAAATAGCTGTTAGTCG

TTAAGTATTAAAGAAAAATATGTTAATACTGGTTTGAGTAAGCTCATTTCACACGGCAAAGTAATCTCAG

ATGATGATAAAAAATATATATATTGGATTTAAA

>AB797210.1 Glycine max GmPhyA3 gene for phytochrome A, complete cds, cultivar: Harosoy_e3

CTTTGATTGTCATACAAATTTAACCGATTGTAACAACTGTCTTAAAGAATTGTATATTTAATATTTTGTC

CATAAAATACCTAAATTATACTTTTGACAACAAATATCTGCAGACTGTAACGAAGCTCATTACATGTTTT

TTTCCCAACCTAACATTAATGTCAATGATACCAACTAGGAATATGCCATTAACATAGGAAGCTAAAAGCT

TTTAAGCTGCAACCTAATTAGGCACTTTTCGTACTAGATATGTCAACAACTCCACTCCACCAATCACCAT

CAAGAATCCGTTTCCTCAGCAGTCGGCACTACAATTAATTTTGAATGGTGCTGTGGGGTTTGGAGGAAGG

ATCATAAAGAAAAGATTTAGAGAAAGTGGGGAAAGCTTGAGAATTAGTGGATGGATCTGAGAGACGAACC

AATAAATCAATGGAACAGATATTGAATTTGCATATGGGTGGGGTAAGGATCATGATGGAGTAGGGAATAG

CCAGCAAGAGAATGAGAAGAATGTAATTAAGGAGAAACTAAAACTGTAATTTGCACACGTTAATGAGGCT

TACTACTTCTTTAACAAGTTGCCCACCGTTCATCTCATTCACATTCTTTATGACAAATGTTAACCAAAAT

CTTTACACTACTAACTAAGAAAATTACAAATTAGATTTTCATTAAAAGTTATTGTTAAATTCAATTTATC

ATAATTTCTATTACACCCTGATTTTAATAAAAATTTATGTTTGATCTTTAATAAGTGTGCCTTGAGGATA

TTAGATAGCAACATCCTTTACTATTAAATCCATAATTAATTTTTGCTGGATCATAATCATTGAATATTTC

GTAATTTACTTTGGGCGGTAATTTTTACTAACAGATCATTTGTTTAAACTTTATACGACAGGAAGAAGCC

TAACGCTATTATTTTTTACGAACTAGTTTGTTTAAAGTAAAAAAGTCATTTGATGATTTTAATTTTTTTG

CAAAAGGTTATAATTTTTAACTTTTAAAAAAATAAAACAATTTTCTTGAGGATAAAAATTATATGATTTA

AAATTATCTTTGAATTAAGAAGTATTTTAAAAAATTGAATCTTATAATATAAACTAATAAATAAAAGATC

AATCGATCAAGTTTTTTTTTTAGAAAAATGTAAATTATATTAAATCCAAAATAATACAACAATAAACGGG

GCATACCCGCAGTTAGAGAAAATCAAAAGTCACCCTGACATAAAAAGACTTATATCATGCTCCTAAAACA

AATACAAGTGTGCTTGTCTATACATAAGAAATTTAATATTGCCAATAACCTCTATTACAGAAAATTAATA

ATTTTCAAAAATCACTTTATTTCTATACAGTATGATGTAGTATAAATATATTGTTGCTCTCAAAAAAATA

AAATTATAAACATCTAACGTGTGGCCGAATGGACTGGTTTATCCATCATGGGATTCAAATCAAATCAAAA

GTCATTGGGTCATGTTGTGTTTAGTTGATTAGAACCCTTTCCAATTTTCAAGCATGTAGCTCGCAAGTCG

CAAGTACCAACTCATGACTGCGACCCAACGTGCACATTACGCTATCATTGAATAAACCAGCTTCCGATAA

AGATACACTACACTTTGCTGATAAACCTCCACTAGAGAGATGCTTCTGTAAATTTAGCATCGGTATTTGA

GTTTATTATATCAGTAAAATACAGAGGCATTGTTAATATATTAATAGGAGTATTGTTAACGTAGATGTAT

TTTAAATATACTTGTGTTTGAAGTTTCACATTAATAATTAAACTTAACTTTTAATATTAATTTTTATTAT

GCAAATAATCATTCAAGTTTAATCAGATATTTTAAATTCGAATCTTAGATATTCATAGCAAAAAAAAATT

CGTCTATAATAATTTTATTCGACTTAAATAAAATTATTTTTGATAAATAAATGATTCATGTGATCTTATA

CAAAAATTATGAAAGCAAAAGTTTAATTATAATAAAAATCTTAAAACGTTGAACACAATAACAAGAGTTA

TAATAATAATAAATTAAGTCACATAAATGATTAATGTGGGCAAGTTAAAAATAAATGTTCAATACTATTT

GAGTTTGATATTTAATAAAAATAAATTTTTATCAGATTTTATTTATAATTAAAATATTATTTTTTAATCA

ACAAGAATATTAAGAAAAAAAATATTATAATATCGGTAGAAGAAAATATTAGTTAGTTTCTATAACCCGA

CGAACCACAATAACTAAATAAACAAATAAATATTGTACCACTGTAAATACCGTACAGAAACTTATGATTC

CGTGTGTGTACTTTTGATCACATTGTGGCTTATGATGTTAGTACTTTTTAGTAGTTAAGCCGAAAGCCGC

AAGCCGCAAGACCCTCCCTGCTTTGAGGGCTGCACGTGACACCCATAACTCTTTTTTTTCTTGTTGCCAT

TCGCTAAGATGATATCCAATTAAAATAAAAATGAGTGACCCAGATAAAAGTTTCCTTCTAGTTTTTTTTT

TTAATTATTAGAATGTTAGTTTTGTTTGTTAAAAAAATTAAACTCGTAATATTTTATTTTTTTAAAACTA

TTCAACCATCTTATATCTGCTGTTTCCTTGCAGTAGTTCATAATAAACCTTAATTAGAAGCAAACCCACT

TTGAAGCCAAAATTTTTGTTGGGTATTTTCTTCTCTTCCCAAAAGGGAAAGTCAGAGTTGAAGGTGATGA

TGCAGATCGTGATCAGAGTCCGTAAAAGCTGCATGTTGCTATTTACGGCTGCAATCATCATCACATGTCC

CTTCCGTATTTTGCCTATTTAGAAACCAATCCACTCTTTACTATTACTAATATGCTATAATGATTGAATA

ATACTAATACTTAAATATAAGTAAGCACCAGAGAGAGAGAGAGAGAGAGAGAGAGAGAGAGAGAGTATTA

AATTATTATTATGGCATGGCAGTGTCTCAGTTCTAACTGCTGAGTTCTGTTCTGTGAGCTTGAAGGGGTT

CCAACTTCCAAGTTCCAATCCCAAAGGGCGGTGATTAAGATTTTGTGCTTCGTCACAATTCACAACCAGA

CATATATATGCAGATGCAGCTGTAACAGTAAAGTTGCTAGCGGGGATTTTTATTCCCAAGCTACTTCTGC

TTCTAATAAAAACCTCACCCTTCTCTCTGCTCATCAGAATTTTTCCTTGCAAGGTGGGTTTTCCCTTTCT

TCATTTTCTTTCTCCTTGCTTTAGTTGATTCTTCCTTCTTTTTGGGTAGGTTGGGTTATCACTACCCCTT

ATTCTCTTAAATTTGCTTTTCTTAACTTTTCTGATGTATGTACTCTTTTTTGACCACGGAAAATTCTTGT

TTACCGTACTCTGGATGATGTTTTCTGTAGTTTCCGGGTCAATTAAGGTTCCGTACCAATTACTGTTCTT

GACAATTAAAGTGGAAAAAATGAAAATCAAATTGCAAAAAAAAAAAAAAAGAAAAAGAAAGAGAAACTAT

TGTTTTGCTGAAAAAGTGTATAGTTGAGTGCATGCTCATGAGTTCCTCTGAAGAATTTATAACCTCTTAC

CATATGCATCAGATAACAGTGGAAGATTAATGATAAAGATTCTTAATATTATTATTGTTGTTTGATCTTT

GGATAGAGTATTACAACAGGATATGATTGGATCATTAATTATCATTACTTACTGAAATCCTTTCAAAGTT

TTGTTCTTCATGTGTGGAAAAATCCACTTTCTCTTCTACAATTGTGACAGTGAGTAAGGATGATGTGAAT

TGACTAGGTCCATACTCTTAGAAGCTTTGCATCTTTACTAGAGCAAACAGTTTCCATTCTTTCTTGTGAG

AGATTAACTTAGTGATTCTATGCTTGTGGTTTCAAGTGCAGTTGAAGTGACAATGTCCTCTTCAAGGCCC

AGCCAATCATCCAGCAATAATTCTGGCAGATCTAGAACATCAAGACTCAGTGCTAGGAGGATGGCTCAGA

CAACTTTAGATGCAAAACTGCATGCAACTTTTGAGGAATCAGGTAGTTCTTTTGACTACTCCAGTTCAGT

GAGAATGTCTCCTGCTGGTACTGTCAGTGGAGACCATCAACCAAGGTCTGATAGAGCAACAAGTTCTTAC

CTCCATCAGACACAGAAAATCAAGCTTATCCAGCCATTTGGGTGTTTGTTAGCTTTAGATGAGAAAACAT

GCAAGGTCATTGCTTACAGTGAGAATGCACCTGAAATGCTCACCATGGTTAGTCATGCTGTCCCCAGTGT

AGGTGACCACCCTGCTCTTGGCATTGGCACTGACATAAGAACTATTTTCACTGCCCCAAGTTCTGCTGCT

ATTCAGAAGGCACTGAGATTTGGGGATGTTTCACTTCATAACCCCATTCTAGTCCATTGCAAGACCTCTG

GGAAGCCCTTTTATGCAATTATCCATCGTGTTACCGGTAGTGTGATCATTGATTTTGAGCCGGTCAAGCC

TCATGAAGTTCCCATGACTGCATCAGGAGCCCTGCAATCCTACAAGCTTGCAGCAAAAGCAATAACTAGA

TTGGAATCCTTGACTACTGGGAACATGGAAACACTATGTAACACAATGGTTCGAGAGGTTTTTGAGCTCA

CAGGTTATGACAGAGTGATGGCTTATAAATTCCATGAGGATGATCATGGGGAAGTGATTGCTGAGGTTAA

AAGGCCAGGCCTAGAGCCATATCTGGGGTTGCACTACCCAGCCACTGATATTCCTCAGGCGACACGCTTT

TTGTTTATGAAGAACAAGGTGCGTATGATAGTTGATTGTTGTGCAAAGCATGTGAATGTGCTTCAAGACA

AAAAAATTCCATTTGATTTAACCTTGTGTGGATCAACCTTGAGAGCTGCTCATAGTTGCCACTTGCAATA

CATGGAGAACATGAATTCTAGTGCTTCCTTGGTTATGGCAGTTGTGGTAAATGACAATGATGAAGATGGG

GATAGTTCTGATGCTGTTCAACCACAGAAGAGTAAGAGACTCTGGGGTTTAGTAGTTTGCCATCACACTA

CTCCCAGATTCGTTCCTTTCCCTCTTAGGTATGCTTGTCAATTTCTGGCTCAAGTATTTGCGGTTCATGT

GAGCAAAGAGCTAGAGATAGAGTATCAGATTATTGAGAAGAACATCCTGCAAACTCAAACACTCTTGTGT

GATATGCTGGTGCAAGGTGAGCCCCTAGGCATTGTTTCACAAAGTCCTAATATAATGGATCTTGTGAAGT

GTGATGGAGCAGCCCTGCTATATAAAAACAAGGTGTGGCGATTAGGGGTAACACCAAGTGAATCTCAGAT

AAAAGAGATAGCTTTGTGGCTCTTTGAGTGCCATGAGGATTCCACAGGTTTTTGTACAGATAGCTTGTCT

GATGCAGGCTTCCCTGGGGCTGCTGCTCTTGGTGATATTGCATGTGGAATGGCAGCTGCCAGAATAGCTT

CCAAAGATATACTTTTCTGGTTTCGGTCTCACACAGCCTCAGAAATCCGATGGGGTGGTGCAAAGCATGA

GCCTGGTGAAAGGGATGATGGTAGGAGGGTGCATCCAAGATCATCATTCAAGGCTTTCCTTGAAGTTGTG

AAGACAAGGAGCTTACCCTGGAAGACCTATGAAACGGATGCCATTCATTCGTTGCAGTTAATACTGAGAG

ATGCATTCAAAGAGACACAGAGCATGGAGATAAGCACATATGCTATCGATACAAGGCTAGGTGATTTGAA

GATTGAAGGAATGCAAGAACTGGATGCAGTGACAAGTGAGGTGGTAAGGTTAATTGAAACAGCAACGGTG

CCAATTTTGGCGGTTGATGTTAATGGGATGATCAATGGATGGAACACAAAAATTGCTGAGTTGACAGGTC

TTCCAGTTGATGAAGCTATTGGAAAGCATTTACTCACACTTGTAGAGGATTTTTCAGTAGATAGAGTCAA

GAAGATGTTGGACATGGCATTGCAGGGTATGCCTTTTTTCCTTTCCCCGTCTTATTCTTATTCTAGCTCT

TATGTTCTTTTACTTTTTTTTGTTAGGTTTTAGCATTCATAAGCAAACTTTGAAATGAGTGTAATGATAT

TTAGATTTGGGGATGTTATGTTCTCTACAACCATCCATAAATATTTCATTAATAAAGAATAACAGCTTCC

AAGTTTTATTCAATTTTAAAAGACTTAGGAAGGAATGGAGCATTGCAAAAGATACTTTATCCTAGCTTTC

CATATTTCTACTTAATTGATTTGTTATGCTACAATATGATTAGGTGAGGAAGAGAGAAATGTCCAATTTG

AGATCCAAACACATCATATGAAGATTGATTCTGGTCCCATCAGCTTGGTAGTTAATGCTTGTGCAAGCAG

GGATCTTCAAGATAATGTTGTGGGAGTTTGTTTTCTGGCACAAGATATAACTGCTCAGAAAACAATGATG

GACAAATTCACCCGAATTGAAGGTGACTACAAGGCAATTGTACAGAACCCAAACCCATTGATCCCTCCAA

TATTTGGCACAGATGAATTTGGTTGGTGTTGTGAATGGAATTCAGCTATGGCAAAATTAACTGGATGGAA

GCGAGAGGAGGTAATGGATAAAATGCTTTTAGGAGAGGTTTTCGGGACCCAAATAGCTTGTTGTCGCCTA

AGGAATCATGAAGCTGTTGTTAACTTTAGCATTGTACTTAATACAGCCATGGCTGGTTTGGAAACAGAGA

AGGTTCCTTTTGGTTTCTTTGCTCGTGATGGAAAGCATGTAGAATGTATTCTTTCTATGACTAAGAAATT

GGATGCAGAAGGTGTAGTTACTGGTGTCTTCTGCTTCTTGCAACTAGCAAGTGCAGAGCTGCAACAAGCA

TTACACATTCAGCGCATATCTGAACAAACTTCATTGAAAAGACTGAAAGATTTAACTTATTTGAAAAGGC

AAATCCAGAATCCTTTATATGGGATTATGTTCTCCCGGAAATTGTTAGAGGGTACTGAGTTGGGAGCTGA

ACAAAAACAATTTCTGCAAACGGGCATTCGGTGTCAACGCCAGATTAGCAAAATTCTGGATGACTCGGAT

CTTGACAGCATCATTGATGGGTATGATATCTGTGAATGTGTTGCTTCTAGTTTTTATTTTTACTTGCCAT

TTGTGTGGTATTTTGAGCATTTATCAACAAGAGTTCCTATTAAATCATACAATTTCGTATTTCATAACCA

CACCTAATTTATGCTTCTAGTCTTTTGTCCTTGTCATTTGTGTGATGTTAAATCATTTATCAACAATAGC

CCCTATTAAATCAACCACACTTAATATTGAGTGTCAAATAGTTTATAGTCAAACATGAAAGAGATCAGGA

TTTGAACTGTGTTCTGTTTTGATAAGTGAATACTGACTCCTGAATTTCAGGAATCAGATTCTTTACAACT

ACTTTTTTTTTTCCAGTAAAGACCTTGATGTACTAGTGGCCCTTTTGTCCTGCATAACAGCTACATGGAT

TTGGAGATGGTTGAATTCACTTTGCATGAAGTTTTGGTTGCCTCCCTAAGTCAAGTCATGACAAAGAGTA

ATGCAAAAGGTATCCGAGTAGTCAATGATGTTGAAGAGAAGATCACAACAGAGACCTTATATGGTGATAG

TATCAGGCTTCAGCAGGTCTTAGCTGACTTTTTATTGATTTCCATCAATTTCACACCAACTGGAGGTCAG

GTTGTTGTAGCAGCCACGCTAACCCAACAGCAGTTAGGGAAATTAGTTCATCTTGCTAATTTGGAGTTCA

GGTAATCAACTATTCCAGACTCAGATGGACATTTCAAACTTTCTTGTGGGTCTTCAGTTCAGTTGGTTTA

AATGCCTGCAAAATAAACGTGACATGAAAAGAAACATATTATCACTTTTTTATGAGTTTAGTTTAACAGT

ATAAATGTTTTTACAGTCAACTAACTAAAAATTACCTTGTAATTGGAGGGTATTGGATGATGCTTTGTCA

AAGTTAATGAAGCTTTGTAATGATGCTTTTATTTTTTCTATTTATTTATTTCTCTAAAACTAAAAAAAAA

TATTTTTATCAAACTAAATATAACTATTTTTATTTTATTTTTCACCTATTTTCATTCACTACATGTCTCT

CTTTCCTTTTCTTATCGATTATTGTGTTTCTATTTTTTTCACTTTTTAATTATGAAACTGTCACCTTATT

CTTAATTTATTTTTCGTCGTAAAGAATTGTATAGGACACAACGAAAACAAATTGTTTCCCCAATATTTCA

TGTTTAAATCTTTAAAAACAGAACAGATTAAAAATAAGATGATAAATTTGTCACTAAAAAGTAAAAACAC

AAACAAATGAGTTTTTTGTAATCCTACATTATTTTTTCTCTTTTCTGCTTAGTTTGAAAATACCACCCCA

TCCTTTAGAAAGTGGGATCGATGTGTCCATTTGACATTTCGGTTCCATTTCGCCATATGCATTTTGTTCA

CTTAAAATTTTAAACTTTCAGAAATTATAGGCACTAATGCTTTTAGTTTCATTGAGGATGCCAATTTAAA

AAAGAAAAATATGGGACACTTGCTCCTTTTTAAACTTAGAATGATTAATTGTATAAATATCTCTTATATT

CTCCATCTTTAAAACAGGACATTGATAGCATCAGGTGTTTTGATATGTCCCTCAAATGCAAAGAACGTGA

TTATCATGTGCATTCCTTTTGACAAGCAGCTACCTGAATACAGATATTGATGGGCTCATTTACTATAATG

TCTTTGTTTCTGTGGGACTAGCTGGCTCTGGCTGAATTAACAAAACAAGAAAATAAAAACTACCATGCAT

TGCATGGTAACTAACATGTACACGTTGTGCAGTTAATAAATATCATGCCAAAGTGATTCACTTGTAAGGT

GAAACTGTGAAAGGTCCTTCCTTTAGGGAAGGGATGATAGCAATACCAGTTCTGACCCACCACACCACAC

CAATCAAACAGGATGAGGGAACACTCAAAAAAGTGTTTTTCAGTTGACAGTGATATTCAAACAAAAAAGA

TTTAGCATGATTAAGATACTAGAAGTTAGACTGCTATTTTGAAAGAGACAGTTCAAATGTAAGATGGATT

TTATGAGTGGAATGAGATTCACAAAGTAAAGCTAAAGGCAAAGGCTCTTGAGTTTCTGTGATGTGCATGA

TAAGAGAGGGAAAGTGACACTCATGGACTTTTATCAAACCAATGGAGCTCTAAAAAGGACATTGAAGACA

CCATATGAGCTGTACCATGTGCCCAATAGTTTCAACTTTGAATATTAATTTGACTTAGGATTTCGGTTTT

TCATAACATATACATCTATTTTTTTACTGTCCTAGCCTCTAGGGATGGTACTAGAAGGTCAGCTTTGGCC

AAATGGGTAGCTTTATGTGTTCCCTTCGTAGTTCGTACACATGGAAATTTTTGGGCTATATGTGATTTTT

TTTTTCATAGAAATAAATAAAATAGATTTTTTCCCTCTGTCAAAGGGATCCAGATTCTCTACCGTTAGAG

AAATCCTACATTCATGCCGTCAGACGATTAAAACTGTGTAATTAAGATCTAACGTCTCATAAATAAATCA

GTAAATTTAACTTAAAAAACAGTTTTAAAATACTAATAAAATCAGAGTCCATAATCATGACTGCGTGAAT

GCAGGGAATCCAAATCCCTGACAAAGCTGCTAACTTAAACAAAAGGACAAGTTTCAATTTTAACCTTAAT

CCTTTCATACTTATCATGATAATAATTAATTATCCTAGCTTTGATACAACATACAAGGTTGCTGCTGCCA

GTGCCAGCTTTAATTTAACTACTAATATTAATTAAGAGTACACGTTGCTGATTTCACACACCCAATTTTG

AATTACGAACCATTACAAGATACAAGTTCTCATTAGGGCCTATCAGGGTTATTGTTATGACATAATTAAG

AGCTAAATGATGAGGAAATCACGTGATGTAAGTATGATGCAGCTTTAAAGTTAATAAATAGTTGATGAGT

TTGAACTGTGACATGACCACTTAATCAAATAATGCTATGTATCAAGTAGAGAAAAGTCTACGCAATTTCG

TAAAGCAGTCTTTTCCTTTCAATTTGACCCATGAAAGCTTCTGGTTTAGGAAGTAGATAAATGCCAGCAA

ATGGATTGTTCAACGAAGACAAGCATATAGATGGAGTGGGGTGGGGCAAGTATGTTGGGTTGATTAAGAT

AATTGATAAATTGAATGACTAAGCCTCCACAATTAGTAGCCTTAAACAACCACTTGTGCCTCCAAATCAG

TGAGTGATGAGTGTCATGCCCTGTCAAGAATACAACCCAAATCTTTCTTTTTCTGTTTTGGATTTGAAAA

TGCCAAACTGGTGAGCTAAGAGGCCTTGAGAAAGGAGGGACAGAGAGCATGATGAGTAGCATGGAGGACA

ATTAGTCTAGGATAGAGGGAATCAATTTAAGGTCATGGAAAATTATAAACTAGGTCCTCTTGTGCATTTT

ATGCTCAAAGTTGCTCTCTCTCTTTTACCATCCTTCTCTGAGCTCTAGCTTTTCTCGAAAGCTTCCTTTT

TGGGCTATATGTTCACAAACACCATTTCCCCTGAACACCCAATTCCTGTCTAAAACACACTTCTCAATAT

CTATCTCTAGAATGGTAAGCCACTAAGTCTAGTGACTAGTGACTCATTCATTCAGTTCAAGTGTTCAACC

TTCCTTCTCTATCTCCCTTTCTAACTTCTCCCTATGTGTGTATAGTTGATGATTTTCAAATTCTTGTCAT

ACAGGCTAACCATGATGCAAAAGAAGAACAAAGGCCACTCAACCAATGGAGAAGAAGCAAAGGGGGATTC

ATGGCTTCCATGTTCATTTTTGGTAAGCAAAAAAAAAAAAAAAAGACTCTTACTCTATGTTATGTGATTT

CTCAGCAAGATATATGTTAAGCTTTTTTTGCCCCCTTTCCCTTTCTTGTTGTTCATTGTATAGTCTTGTC

AGC

>EU428747.2 Glycine max phytochrome A-2 (phyA) mRNA, complete cds

AGTGAAAATGTCTACCTCAAGGCCTAGCCAATCATCCAGCAATTCAAGGAGATCAAGACATAGTGCTAGA

ATGGCTCAGGCAACTGTAGATGCAAAAATCCATGCAACTTTTGAGGAGTCCGGTAGTTCCTTTGATTACT

CCAGTTCGGTGCGCGTCTCTGGTACAGCTGATGGAGTCAATCAACCAAGGTCTGACAAAGTTACAACAGC

TTACCTCAATCGCATGCAGAGAGGCAAGATGATTCAGCCTTTTGGTTGCTTGTTGGCCATTGATGAGAAA

ACATGTAAGGTCATTGCATACAGTGAGAACGCGCCCGAAATGCTGACCATGGTTAGCCATGCTGTCCCCA

GTGTTGGTGACCACCCTGCCCTTGGCATTGGCACTGACATAAAAACTCTATTCACTGCACCAAGTGTTTC

TGGATTGCAGAAGGCTCTAGGATGTGCGGACGTTTCGCTTCTTAACCCCATACTTGTCCATTGCAAGACC

TCTGGGAAGCCCTTTTATGCAATTGTCCATCGCGTCACTGGTAGTTTGATTGTTGACTTTGAGCCAGTCA

AGCCTTATGAAGTTCCCATGACTGCAGCAGGTGCCTTGCAATCTTACAAGCTTGCTGCCAAAGCAATTAC

CCGATTGCAATCATTGCCTAGTGGGAACATGGAAAGACTATGTGATACTATGGTTCAGGAAGTTTTTGAA

CTCACAGGTTATGATAGGGTGATGGCTTATAAATTTCATGAGGATGATCATGGAGAGGTGATTGCTGAGA

TAACAAAGCCCGGTCTTGAGCCATATCTGGGTTTGCACTATCCAGCCACCGACATTCCCCAGGCTTCACG

CTTTTTATTTAGGAAGAACAAGGTTCGTATGATAGTTGACTGTCATGCAAAACACGTGAGGGTTCTTCAA

GATGAAAAACTCCAATTTGATTTGATTTTGTGTGGTTCCACCTTAAGAGCTCCTCATAGTTGCCACGCGC

AGTACATGGCTAACATGGATTCAATTGCTTCCCTGGTTTTGGCAGTTGTAGTCAATGACAACGAAGAAGA

TGGGGACACTGATGCTGTTCAGCCACAAAAGAGGGAGAGACTTTGGGGTTTGGTAGTTTGCCATAACACT

ACTCCCAGGTTTGTTCCCTTTCCTCTAAGGTATGCTTGTGAATTTCTGGCTCAAGTATTTGCCGTCCATG

TGCACAAAGAAATAGAGTTAGAATATCAGATTATTGAGAAGAATATCCTGCGCACCCAGGCACTCTTGTG

TATGCTGATGCGAGATGCACCCCTAGGAATTGTATCAGAGAGTCCTAATATAATGGATCTAGTTAAATGT

GATGGAGCTGCCCTCATATACAGGAACAAAGTATGGAGATTAGGAGTGACACCAAGTGAACCCCAGATAA

GAGAGATAGCTTTGTGGTTGTCTGAGTACCATATGGATTCCACAGGCTTTAGTACAGATAGCTTGTTTGA

TGCAGGGTTCCCATCGGCTCTTTCTCTGGGTGATGTTGTGTGTGGAATGGCATCTGTTAGAGTAACTGCA

AAAGACATGGTATTTTGGTTTCGGTCACACACTGCTGCAGAAATCCGATGGGGTGGTGCAAAGCATGAGG

CTGGAGAAAAAGATGATAGTAGGAGGATGCATCCAAGATCATCATTCAAGGCTTTCCTTGAAGTTGTGAA

GGCAAGGAGTTTACCTTGGAAGGAATATGAAATGGATGCTATTCATTCCTTGCAGATAATACTGAGAAAT

GCATTCAAAGAAGATACCGAGAGTTTGGATTTAAACGCAAAAGCAATTAATACAAGACTAAGTGATTTGA

AGATTGAAGGGATAAACGATTTGAAGATTGAAAGGATGCAGGAACTGGAAGCAGTGACAAGTGAGATCGT

TAGGTTGATTGACACAGCAACAGTGCCTATTTTGGCCGTTGATGTTGATGGGCTGGTCAATGGGTGGAAC

ATAAAAATTGCTGAGTTGACGGGTCTTCCAATTGGTGAAGCTACTGGAAAGCATTTACTCACACTTGTTG

AGGATTCTTCAACTGATAGAGTCAAGAAGATGCTTAACTTAGCACTGCTAGGTGAAGAAGAGAAGAATGT

CCAATTTGAGATCAAAACACATGGGTCTAAGATGGATTCTGGTCCTATTAGTTTGGTAGTAAATGCTTGC

GCGAGCAGGGATCTTCGAGATAATGTTGTCGGGGTTTGTTTTGTGGCCCATGATATAACTGCTCAGAAGA

ATGTCATGGACAAATTCATCCGTATTGAAGGTGATTACAAGGCAATTGTACAGAACCGCAATCCATTAAT

CCCTCCTATATTTGGCACAGATGAATTTGGCTGGTGTTGTGAGTGGAATCCAGCTATGATGAAGTTAACT

GGATGGAAGCGAGAGGAGGTGATGGATAAAATGCTTTTGGGAGAGATTTTTGGCACCCAGATGGCTGCTT

GTCGCCTAAAGAATCAAGAAGCTTTTGTTAATTTGGGCGTCGTACTTAATAAAGCCATGACTGGTTCAGA

AACAGAGAAGGTTCCTTTTGGTTTCTTTGCTCGGAATGGCAAGTATGTAGAATGCCTGCTTTCTGTGAGT

AAGAAATTGGACGTAGAGGGCCTAGTTACTGGGGTCTTCTGCTTCTTACAGCTAGCTAGCCCAGAGCTCC

AACAAGCATTACATATTCAGCGTCTATCTGAGCAAACTGCTTCGAAGAGATTGAATGCATTAAGTTACAT

GAAAAGGCAGATCAGGAATCCTTTGTGTGGAATTGTATTTTCCCGGAAAATGTTGGAGGGTACTGACTTG

GGAACAGAACAGAAACAACTTCTGCGCACTAGTGCTCAGTGCCAGCAGCAGCTTAGTAAAATTCTTGATG

ACTCAGATCTTGACACCATCATAGATGGTTACTTGGATCTTGAGATGGCTGAGTTCACCCTGCATGAAGT

TCTGGTTACCTCCCTTAGTCAAGTCATGACAAAAAGTAATGGAAAGAGTATCCGAATAGTCAATGATGTT

GCAGGGCATATTATGATGGAAACTTTATATGGTGATAGTCTTAGGCTTCAGCAGGTCTTGGCTGACTTTT

TACTTATTTCCATCAATTTCACTCCAAATGGAGGTCAGGTTGTTGTAGCAGGCTCTCTAACCAAAGAACA

GTTAGGCAAATCTGTCCATCTTGTTAAGTTAGAGCTCAGCATAACACATGGTGGGAGTGGGGTACCAGAA

GTGTTACTGAATCAGATGTTCGGAAACAATGGGCTAGAATCCGAGGAGGGTATTAGCCTACTGATAAGCA

GAAAGCTGCTGAAGCTGATGAATGGAGATGTTCGCTATCTAAGGGAAGCAGGCAAATCAGCTTTTATCCT

CTCTGCTGAACTTGCTGCAGCCCATAATTTGAAAGCTTAAAAGTTTTGGAAAAAAGAAAAAAGATGATGC

AACCAGC

>AB468154.1 Glycine max GmPhyA3 gene for phytochrome A, complete cds, cultivar: Harosoy

GCAAACCCACTTTGAAGCCAAAATTTTTGTTGGGTATTTTCTTCTCTTCCCAAAAGGGAAAGTCAGAGTT

GAAGGTGATGATGCAGATCGTGATCAGAGTCCGTAAAAGCTGCATGTTGCTATTTACGGCTGCAATCATC

ATCACATGTCCCTTCCGTATTTTGCCTATTTAGAAACCAATCCACTCTTTACTATTACTAATATGCTATA

ATGATTGAATAATACTAATACTTAAATATAAGTAAGCACCAGAGAGAGAGAGAGAGAGAGAGAGAGAGAG

AGTATTAAATTATTATTATGGCATGGCAGTGTCTCAGTTCTAACTGCTGAGTTCTGTTCTGTGAGCTTGA

AGGGGTTCCAACTTCCAAGTTCCAATCCCAAAGGGCGGTGATTAAGATTTTGTGCTTCGTCACAATTCAC

AACCAGACATATATATGCAGATGCAGCTGTAACAGTAAAGTTGCTAGCGGGGATTTTTATTCCCAAGCTA

CTTCTGCTTCTAATAAAAACCTCACCCTTCTCTCTGCTCATCAGAATTTTTCCTTGCAAGGTGGGTTTTC

CCTTTCTTCATTTTCTTTCTCCTTGCTTTAGTTGATTCTTCCTTCTTTTTGGGTAGGTTGGGTTATCACT

ACCCCTTATTCTCTTAAATTTGCTTTTCTTAACTTTTCTGATGTATGTACTCTTTTTTGACCACGGAAAA

TTCTTGTTTACCGTACTCTGGATGATGTTTTCTGTAGTTTCCGGGTCAATTAAGGTTCCGTACCAATTAC

TGTTCTTGACAATTAAAGTGGAAAAAATGAAAATCAAATTGCAAAAAAAAAAAAAAAAAGAAAAAGAAAG

AGAAACTATTGTTTTGCTGAAAAAGTGTATAGTTGAGTGCATGCTCATGAGTTCCTCTGAAGAATTTATA

ACCTCTTACCATATGCATCAGATAACAGTGGAAGATTAATGATAAAGATTCTTAATATTATTATTGTTGT

TTGATCTTTGGATAGAGTATTACAACAGGATATGATTGGATCATTAATTATCATTACTTACTGAAATCCT

TTCAAAGTTTTGTTCTTCATGTGTGGAAAAATCCACTTTCTCTTCTACAATTGTGACAGTGAGTAAGGAT

GATGTGAATTGACTAGGTCCATACTCTTAGAAGCTTTGCATCTTTACTAGAGCAAACAGTTTCCATTCTT

TCTTGTGAGAGATTAACTTAGTGATTCTATGCTTGTGGTTTCAAGTGCAGTTGAAGTGACAATGTCCTCT

TCAAGGCCCAGCCAATCATCCAGCAATAATTCTGGCAGATCTAGAACATCAAGACTCAGTGCTAGGAGGA

TGGCTCAGACAACTTTAGATGCAAAACTGCATGCAACTTTTGAGGAATCAGGTAGTTCTTTTGACTACTC

CAGTTCAGTGAGAATGTCTCCTGCTGGTACTGTCAGTGGAGACCATCAACCAAGGTCTGATAGAGCAACA

AGTTCTTACCTCCATCAGACACAGAAAATCAAGCTTATCCAGCCATTTGGGTGTTTGTTAGCTTTAGATG

AGAAAACATGCAAGGTCATTGCTTACAGTGAGAATGCACCTGAAATGCTCACCATGGTTAGTCATGCTGT

CCCCAGTGTAGGTGACCACCCTGCTCTTGGCATTGGCACTGACATAAGAACTATTTTCACTGCCCCAAGT

TCTGCTGCTATTCAGAAGGCACTGAGATTTGGGGATGTTTCACTTCATAACCCCATTCTAGTCCATTGCA

AGACCTCTGGGAAGCCCTTTTATGCAATTATCCATCGTGTTACCGGTAGTGTGATCATCGATTTTGAGCC

GGTCAAGCCTCATGAAGTTCCCATGACTGCATCAGGAGCCCTGCAATCCTACAAGCTTGCAGCAAAAGCA

ATAACTAGATTGGAATCCTTGACTACTGGGAACATGGAAACACTATGTAACACAATGGTTCGAGAGGTTT

TTGAGCTCACAGGTTATGACAGAGTGATGGCTTATAAATTCCATGAGGATGATCATGGGGAAGTGATTGC

TGAGGTTAAAAGGCCAGGCCTAGAGCCATATCTGGGGTTGCACTACCCAGCCACTGATATTCCTCAGGCG

ACACGCTTTTTGTTTATGAAGAACAAGGTGCGTATGATAGTTGATTGTTGTGCAAAGCATGTGAATGTGC

TTCAAGACAAAAAAATTCCATTTGATTTAACCTTGTGTGGATCAACCTTGAGAGCTGCTCATAGTTGCCA

CTTGCAATACATGGAGAACATGAATTCTAGTGCTTCCTTGGTTATGGCAGTTGTGGTAAATGACAATGAT

GAAGATGGGGATAGTTCTGATGCTGTTCAACCACAGAAGAGTAAGAGACTCTGGGGTTTAGTAGTTTGCC

ATCACACTACTCCCAGATTCGTTCCTTTCCCTCTTAGGTATGCTTGTCAATTTCTGGCTCAAGTATTTGC

GGTTCATGTGAGCAAAGAGCTAGAGATAGAGTATCAGATTATTGAGAAGAACATCCTGCAAACTCAAACA

CTCTTGTGTGATATGCTGGTGCAAGGTGAGCCCCTAGGCATTGTTTCACAAAGTCCTAATATAATGGATC

TTGTGAAGTGTGATGGAGCAGCCCTGCTATATAAAAACAAGGTGTGGCGATTAGGGGTAACACCAAGTGA

ATCTCAGATAAAAGAGATAGCTTTGTGGCTCTTTGAGTGCCATGAGGATTCCACAGGTTTTTGTACAGAT

AGCTTGTCTGATGCAGGCTTCCCTGGGGCTGCTGCTCTTGGTGATATTGCATGTGGAATGGCAGCTGCCA

GAATAGCTTCCAAAGATATACTTTTCTGGTTTCGGTCTCACACAGCCTCAGAAATCCGATGGGGTGGTGC

AAAGCATGAGCCTGGTGAAAGGGATGATGGTAGGAGGGTGCATCCAAGATCATCATTCAAGGCTTTCCTT

GAAGTTGTGAAGACAAGGAGCTTACCCTGGAAGACCTATGAAACGGATGCCATTCATTCGTTGCAGTTAA

TACTGAGAGATGCATTCAAAGAGACACAGAGCATGGAGATAAGCACATATGCTATCGATACAAGGCTAGG

TGATTTGAAGATTGAAGGAATGCAAGAACTGGATGCAGTGACAAGTGAGGTGGTAAGGTTAATTGAAACA

GCAACGGTGCCAATTTTGGCGGTTGATGTTAATGGGATGATCAATGGATGGAACACAAAAATTGCTGAGT

TGACAGGTCTTCCAGTTGATGAAGCTATTGGAAAGCATTTACTCACACTTGTAGAGGATTTTTCAGTAGA

TAGAGTCAAGAAGATGTTGGACATGGCATTGCAGGGTATGCCTTTTTTCCTTTCCCCGTCTTATTCTTAT

TCTAGCTCTTATGTTCTTTTACTTTTTTTTGTTAGGTTTTAGCATTCATAAGCAAACTTTGAAATGAGTG

TAATGATATTTAGATTTGGGGATGTTATGTTCTCTACAACCATCCATAAATATTTCATTAATAAAGAATA

ACAGCTTCCAAGTTTTATTCAATTTTAAAAGACTTAGGAAGGAATGGAGCATTGCAAAAGATACTTTATC

CTAGCTTTCCATATTTCTACTTAATTGATTTGTTATGCTACAATATGATTAGGTGAGGAAGAGAGAAATG

TCCAATTTGAGATCCAAACACATCATATGAAGATTGATTCTGGTCCCATCAGCTTGGTAGTTAATGCTTG

TGCAAGCAGGGATCTTCAAGATAATGTTGTGGGAGTTTGTTTTCTGGCACAAGATATAACTGCTCAGAAA

ACAATGATGGACAAATTCACCCGAATTGAAGGTGACTACAAGGCAATTGTACAGAACCCAAACCCATTGA

TCCCTCCAATATTTGGCACAGATGAATTTGGTTGGTGTTGTGAATGGAATTCAGCTATGGCAAAATTAAC

TGGATGGAAGCGAGAGGAGGTAATGGATAAAATGCTTTTAGGAGAGGTTTTCGGGACCCAAATAGCTTGT

TGTCGCCTAAGGAATCATGAAGCTGTTGTTAACTTTAGCATTGTACTTAATACAGCCATGGCTGGTTTGG

AAACAGAGAAGGTTCCTTTTGGTTTCTTTGCTCGTGATGGAAAGCATGTAGAATGTATTCTTTCTATGAC

TAAGAAATTGGATGCAGAAGGTGTAGTTACTGGTGTCTTCTGCTTCTTGCAACTAGCAAGTGCAGAGCTG

CAACAAGCATTACACATTCAGCGCATATCTGAACAAACTTCATTGAAAAGACTGAAAGATTTAACTTATT

TGAAAAGGCAAATCCAGAATCCTTTATATGGGATTATGTTCTCCCGGAAATTGTTAGAGGGTACTGAGTT

GGGAGCTGAACAAAAACAATTTCTGCAAACGGGCATTCGGTGTCAACGCCAGATTAGCAAAATTCTGGAT

GACTCGGATCTTGACAGCATCATTGATGGGTATGATATCTGTGAATGTGTTGCTTCTAGTTTTTATTTTT

ACTTGCCATTTGTGTGGTATTTTGAGCATTTATCAACAAGAGTTCCTATTAAATCATACAATTTCGTATT

TCATAACCACACCTAATTTATGCTTCTAGTCTTTTGTCCTTGTCATTTGTGTGATGTTAAATCATTTATC

AACAATAGCCCCTATTAAATCAACCACACTTAATATTGAGTGTCAAATAGTTTATAGTCAAACATGAAAG

AGATCAGGATTTGAACTGTGTTCTGTTTTGATAAGTGAATACTGACTCCTGAATTTCAGGAATCAGATTC

TTTACAACTACTTTTTTTTTTCCAGTAAAGACCTTGATGTACTAGTGGCCCTTTTGTCCTGCATAACAGC

TACATGGATTTGGAGATGGTTGAATTCACTTTGCATGAAGTTTTGGTTGCCTCCCTAAGTCAAGTCATGA

CAAAGAGTAATGCAAAAGGTATCCGAGTAGTCAATGATGTTGAAGAGAAGATCACAACAGAGACCTTATA

TGGTGATAGTATCAGGCTTCAGCAGGTCTTAGCTGACTTTTTATTGATTTCCATCAATTTCACACCAACT

GGAGGTCAGGTTGTTGTAGCAGCCACGCTAACCCAACAGCAGTTAGGGAAATTAGTTCATCTTGCTAATT

TGGAGTTCAGGTAATCAACTATTCCAGACTCAGATGGACATTTCAAACTTTCTTGTGGGTCTTCAGTTCA

GTTGGTTTAAATGCCTGCAAAATAAACGTGACATGAAAAGAAACATATTATCACTTTTTTATGAGTTTAG

TTTAACAGTATAAATGTTTTTACAGTCAACTAACTAAAAATTACCTTGTAATTGGAGGGTATTGGATGAT

GCTTTGTCAAAGTTAATGAAGCTTTGTAATTGGATGACAGTGTAAAACTGTTGTACATTGTCTGTGCATA

AACTATTTTCTCTTTTTACATTATACACTCTTGTATGAGAACTAAAGGCTCTCACATTGGTCAAATAACA

ACAACAAACAACAACGCCTTATCCCACTAGGTGGGGTCGACTACATGGATCAACTTCCGCCATAATGTTC

TATCAAGTACCATACTTCTAACCAAACCATTAATTTCGAGATCTTTTTTGATAACCTCTCTTATAGTCTT

TTTGGGTCTTCCTCTGCCTCGAATTGTTTGTCTTCTCTCCATCTGGTCTACTCTCCTCACTACAGAGTCT

ACCGGTCTTCTCTCTACATGCCCAAACCACCTAAGTCTATTTTCCACCATCTTCTCTACAATAGGCGCTA

CTCCAACCCTCTCTCTAATAGCTTCGTTTCTAATTTTATCCTGTCGAGTCTTACCACACATCCACCGCAA

CATCCTCATCTCCGCTACACCTACTTTATTCTCATGTTGGCTCTTGACCGCCCAACATTCTGTTCCGTAC

AAAATCGCCGGTCTTACCGCAGTCCGATAAAACTTTCCCTTTAGCTTGATCGGTACCTTTGCATCACATA

ACACCCCCGATGCTTTTCTCCATTTCATTCATCCTGCTTGAATGCGATGATTCACATCCCCTTCAATTTC

CCCATCATCCTGTATTACAGACCCAAGATATTTAAACCGTGTGACTTGAGGAATAATATGGTCTCCTATT

TTCACCTCTGAGTTAGAAACCCTCCTTCTTTTGTTGAACTTACATTCCATATACTCCGATTTGCTTCTGC

TTAGGCGAAAGCCATGTGTTTCTAGAGCTCGTCTCCAAGTTTCCAACCTCTCATTCAACTCCTCCCTCGA

CTCTCCAAGGAGGACTATGTCATCTGCAAAAAGCATGCATCTCGGCGCTATCTCTTAGATTTGTTCCGTG

AGGACATCCAGAATTAAGGTAAAAAGGTAGGGGCTAAGGGTTGACCCTTGATGTAAACCAATTGTGATGG

GAAAATCGTCTGACTCTCCACCCTGTGTCCTAACACTAGTCGATACCCTATCATACATATCTTGGATAGC

TCGAATATATGCAACCCTAACCCCTTTCTTCTCTAGAGCTTTCCACAAAATCTCTCTAGGCACTCTATCA

TACGCTTTCTCCAAGTCAATAAAAATCAAGTGCAAGTCTTGTTGGGCCATGCGATATTGCTCCATCACCT

GCCGTAATAAATAAATCGCTTCCATGGTCGACCTTCCCGACATGAAACCAAATTGATTCTCAGTAACTTG

AGTCTCCTTTCTTAATCTCCGTTCGATCACTCTTTTCCATAATTTCATGGTATGACTCATGAGCTTGATT

CCCCTATAATTTGCACAATTTTGTATATCCCCCTTGTTCTTATAGATTGGCACTAACGTGCTTCTCCTCC

ATTCCTCCGGCATGCGTTTTGACCTCATAATTTCGTTAAAGAGTTCGGTGAGCCACTCAAGACCTCTATC

TCCAAGAGTTTTCCACACTTCAATAGGTATGTTGTCTGGCCCCACCGCCTTACCATTACTCATTCTTTTC

AACGCTTCCTTTACTTCCTGTTTTTGAATCCGACGATAGTACTTATAGTTCCGGTCCTCTTCTCTTGTGT

CTAGACTGCTAGAGTCATATCCATATCCATCATTAAATAAGTTGTGGAAATACGCCTTCCACCTTTCCTT

GATATCTTTTTCATGCACTAAGACTTTGCCTTCTTCATCCTTAACACACTTTACTTGATCCAAATCTCTA

GTCTTCCTCTCTCTACCCTTAGCAAGCCTATATATAGATCTTTCTCCGTCCCTGGTTCCTAGAGCTTGGT

ATAGTCCGTCAAAAGCTTGGGCTCTTGCCTCACTCACCGCCTTTTTGGTTTCATTTCTAGCTATCTTATA

CTTATCCCAAGTTTCAGAATTTCTACACCTAGACCACTCCTTGAAACACTCCTTTTTTACTCTAACTTTG

CTCTGAACATTTTCATTCCACCACCACGATTCTTTACCCCTAGGTCCAAAACCTCTAGATTCACCCAACG

TCTCTTTAGCCACTTTAATAATCTCTTGGGACATCTTGTTCCACATATCATTTGCACTTCCTTGTGATTG

TCCACACCAACCCTCCCATATCTTTTGTTGGAAGATTCCTTGTTTCTCACCCTTCAAGTGCCACCATTTG

ATCCTTGGTGCTACCAGAGGACTTCTTCTCTTTGCCCTATCTCTAATTCTTACATCCATAACCAAAACTC

TATGTTGGGTAGTCAAGCTCTCTCCCGGGATAACTTTACAGTTCAAGCAATACTTCCTATCAGACTTCCT

GATAAGGAAGAAATCTATCTGAGAACATGTCCCTCCACTTTTGTAAGTGATAAGATGTTCCTCTCTTTTC

TTAAACCATGTATTGGCTATAGAAAGATCCAAAGCCTTCGAAAACTCCAAGATGGATTTACCCTCCCCAT

TCATCTCCCCTAGGCCAAAACCCCTATGCACCCCCTCAAAACCTCTAGCCACGCTACCTACATGTCCATT

GAGATCCCCTCCTAGGAAAACTTTCTCTCCTTGGGGTATATCCTGAAGTACCCCTTCTAGATCCTCCCAA

AATTTTACCTTAAAGTGTTCTGCTAACCCAACCTGAGGTGCGTACCCACTAATAACATTAAAGGTGTCCT

GTCCCACTACCAATTTTAAGACTAATAACATTGGTCAAAACGACAAAAAAAAATGTGTGTATGGAGGGGT

TGAGAGCAATTGGTTTGAATCTTTTGGATGATACCTTCCTATGCTTGATACACGCCGTTTTGAGATTATA

TACGTGATTTCTAAGTCATTGTGTTTGCTTTTGTTATTTTTTGCAGCATAACGCATGATAGTTTTGGGGT

TCCAGAAACATTGCTGAACCAGATGTTTGGACGCGATGGACATGAATCTGAGGAGGGTATTAGCATGCTG

ATTAGCAGAAAGCTGCTAAAGCTCATGAATGGAGACGTACGTTATTTAAGGGAAGCAGGCAAATCATCTT

TCATCCTATCTGTTGAACTTGCCGCAGCACATAAATCCAACACTTAACATTTTGGAAATAAAACCAAACC

ATATTTTTTGTACATCAGAAAGTGAAAGGAAGCAAGAAATTTGCATTCCTTGTTCAATATTTAACTCTTG

TTATTGATTACCTTCTTGTAAGGTGATGTAGATATGTAATGTATAAGTGTATAACTAACTATTTACACAC

TATAGACCTGTTCTCATCTACAGCAATGATGCTTCATTTGCAACAAAATGAAACATAGGGACTAACTAGG

AGCATGTTAAATGGCATTGTTTTCTACCTTTATTTCATTTTCTTCCAAAACTTTATTTAAAACATCCTGC

TTAGAGATACCATAAGTATTAAATATTAGTAAAGTATTTCTTATAAATACTTTTAATTACTTCAAAAATA

AATATTAAATATTTTTTTTAAGTAATAAATAATTTTTGTTATCTTCATTTTTTCATCTGTCAGTGTCACT

TTAAAAATAGTTATTTATTTGTCATTTTAAAATTCTAAAATGACATTAGTTACTTTTTTTTAAAATTATA

TACTTATAAAAAAAGAGAGAGACATAATAATATATGAATGCCAAAAAATAAAATAAATAAGAGAGGGAAA

AACATATTAAAAAATGATAAATAATTTTAATAAAAAGTAGGAATTTATTAACTTTTCTCAATTTATGTTC

AATAATAAATAAAAAGAAACAATAATATTTTTAGGGCATAAACATGTATATGTGATCTTAAATAGTAAAT

AATTTAAAATGGAAGGCTTGCAGTTTTATGTGGATAGTCATTTGTTTTCA

>U84970.1 Lathyrus sativus phytochrome type A (phyA) gene, complete cds

ATGTCAACCACGAGGCCTAGCCAATCGTCCAACAATTCGGGGAGGTCAAGAAATAGTGCTAGGATTATTG

CTCAGACGACTGTGGATGCAAAGCTTCATGCAACTTTTGAGGAGTCCGGTAGTTCGTTTGACTACTCGAG

TTGGGTGCGTGTTTCTGGCTCGGTGGATGGAGATCAACAACCGAGGTCCAACAAAGTGACAACAGCTTAC

CTCAATCATATACAGAGAGGTAAGCAGATCCAGCCTTTCGGGTGCTTGCTGGCTTTAGATGAGAAAACGT

GCAAGGTTGTTGCGTATAGTGAGAATGCGCCTGAGATGCTGACTATGGTGAGTCATGCTGTTCCTAGCGT

GGGTGACCATCCTGCCCTTGGCATTGGAACTGACATAAGGACTGTTTTCACTGCGCCGAGTGCTTCTGCC

TTGCAGAAGGCGCTAGGGTTTGCGGAGGTTTCGCTTCTTAACCCGATTCTTGTTCATTGCAAGACTTCTG

GGAAGCCGTTTTACGCGATCATTCATCGTGTTACTGGTAGTTTGATCATTGACTTTGAGCCGGTGAAGCC

TTATGAAGTTCCCATGACTGCTGCGGGTGCCTTGCAATCTTACAAACTTGCTGCTAAAGCAATTACAAGA

TTGCAATCTTTGGCTAGTGGCAGCATGGAAAGGCTTTGTGATACCATGGTTCAAGAAGTTTTTGAACTAA

CGGGTTATGACAGGGTGATGGCTTATAAATTTCACGAGGATGATCACGGGGAGGTGATTGCTGAGATAGC

AAAGCCAGGCCTAGAGCCATATCTAGGTCTGCACTATCCGGCGACAGATATTCCCCAGGCTGCGCGGTTT

CTATTTATGAAGAACAAGGTCCGTATGATAGTTGATTGTAATGCAAAACATGTGAAGGTTCTTCAAGACG

AAAAACTCCCATTTGATTTGACTCTGTGCGGTTCGACCTTAAGAGCTCCACATAGTTGCCATTTGCAGTA

CATGGCTAACATGGATTCAATTGCTTCGTTGGTTATGGCAGTAGTCGTCAACGACAGCGATGAAGATGGA

GATAGCGCTGACGCAGTTCTCCCACAAAAGAAAAAGAGACTTTGGGGTTTGGTAGTTTGTCATAACACTA

CTCCAAGGTTTGTTCCTTTTCCTCTAAGGTATGCTTGTGAGTTTCTGGCTCAAGTGTTTGCCATCCATGT

GAACAAAGAAATAGAGTTAGAATATCAGATTCTTGAGAAGAATATCCTGCGCACACAGACGTTGTTGTGT

GATATGTTGATGCGAGATGCACCCTTAGGTATTGTATCACAAAGCCCTAATATAATGGATCTAGTGAAAT

GTGATGGGGCTGCACTCTTCTATAGAAACAAGTTATGGTTATTAGGAGCGACACCGACTGAATATCAAAT

AAGAGAGATAGCTTTATGGATGTCTGAGTATCATACAGATTCGACAGGTTTGAGTACAGATAGCTTGTTG

GATGCAGGGTTTCCAGGGGCTCTTTCTCTTAGTGATACTGTATGTGGAATGGCAGCTGTGAGAATAACTT

CAAAAGACATAGTTTTCTGGTTTAGGTCACACACTGCTGCAGAAATCCGATGGGGTGGTGCAAAGCATGA

ACCGGGCGAACAGGACGATGGTAGGAAGATGCATCCAAGATCATCATTCAAGGCTTTCCTTGAAGTTGTG

AAAGCCAGAAGCGTGCCGTGGAAAGACTTTGAAATGGATGCTATTCATTCGTTGCAGTTAATACTGAGAA

ATGCGTCCAAAGATACAGATATTATAGATTTGAACACGAAAGCAATCAATACAAGACTAAATGATTTGAA

GATTGAAGGGATGCAGGAATTGGAAGCAGTGACAAGTGAGATGGTTAGATTAATTGAAACAGCAACAGTG

CCTATTTTGGCAGTGGATGTTGATGGGACGGTCAACGGATGGAATATAAAAATCGCCGAGTTGACAGGTC

TTCCAGTTGGCGAAGCTATTGGAAAACATTTACTCACCCTGGTTGAGGATTCTTCAACTGATATTGTCAA

GAAGATGCTCAACTTGGCACTGCAGGGTATGCCTTTTTTCCTTTTCCATCTTATTATCCTTTACTTAAGT

CTCTAACTGTTGATTTTCTTATCTTATAATGTAATCAGGTGAAGAAGAGAAGAATGTTCAATTCGAGATA

AAAACACATGGGGATCAGGTGGAATTCGGTCCTATTAGTTTGATAGTTAATGCGTGTGCAAGCAGGGATC

TTCGTGAAAATGTAGTGGGGGTTTGTTTTGTGGCCCAAGATATTACTGCTCAGAAGACTGTCATGGACAA

ATTCACCCGAATCGAAGGCGATTACAAAGCAATTGTGCAGAACCCGAATCAGTTAATCCCTCCTATATTC

GGTACAGATGAATTTGGCTGGTGTTGTGAGTGGAATGCAGCTATGATTAAGTTAACTGGATGGAAGCGCG

AGGAGGTAATGGACAAAATGCTTCTAGGAGAGGTTTTCGGTACTCAAATGTCTTGTTGTCGTCTAAAGAA

TCAAGAAGCTTTTGTTAATTTCGGCATTGTACTTAATAAAGCCATGACCGGTTTGGAAACAGAAAAGGTT

GCTTTTGGTTTCTTCTCTAGAAAAGGCAAGTACGTAGAGTGCCTACTCTCAGTGAGTAAGAAAATCGACG

CAGAGGGCCTAGTTACCGGAGTCTTCTGTTTCTTGCAGTTAGCTAGCCCTGAGCTGCAACAAGCATTACA

TATTCAGCGCCTATCCGAACAAACTGCTCTAAAGAGACTGAAAGTACTGACTTACATGAAAAGGCAGATC

AGGAATCCGTTGGCTGGGATTGTGTTTTCCAGTAAAATGCTGGAGGGTACTGACTTGGAAACTGAACAAA

AACAAATCGTGAACACTAGTTCTCAGTGCCAGCGCCAGCTTAGCAAAATTCTTGATGACTCTGATCTCGA

CGGCATCATTGATGGGTATGATATCAGTAAATATATTGTTCTCTTAATTATTTTTCACCCTTGCTCACAT

TTCATGTATGCTTAACAGGTACTTGGATCTTGAGATGGCTGAATTTACTTTACATGAGGTACTGGTTACC

TCTCTTAGTCAAGTCATGAATAGGAGCAACACAAAGGGTATCCGAATAGCAAACGATGTTGCGGAGCATA

TTGCAAAGGAAAGCTTGTATGGTGATAGTCTTAGGCTTCAGCAGGTCCTAGCTGACTTTTTACTAATTTC

CATCAATTCCACACCTAATGGAGGCCAGGTTGTTATAGCATCCTCCTTAACTAAAGAACAGTTGGGAAAA

TCTGTCCATCTTGTTAACTTGGAGCTCAGGTAACTGTTCCATATTCACGCATGTATCGATTTTCAGTATC

CGATGATTTAAGCCTCGGTAATAATCTTAGCTTTGGTGAAACTGCTAACTGCAATAAACTAATATCTGTA

TATATTTTGTTTGCGATTTGTGTAGCATAACACACGGTGGTAGTGGCGTGCCAGAAGCGGCGCTTAACCA

GATGTTTGGAAATAATGTGCTAGAATCTGAGGAGGGTATTAGCCTACACATCAGTCGGAAGTTGTTAAAG

CTTATGAATGGAGATGTTCGTTATTTAAAAGAAGCAGGAAAATCATCGTTTATTCTATCTGTTGAACTTG

CAGCAGCTCATAAGTTGAAAGGTTGA

>AB767256.1 Lotus japonicus PHYA gene for phytochrome A, complete cds, haplotype: G

ATGTCTTCATCAAGGCCTAGCCAATCGTCCAACAATTCAGGGAGATCAAGACATAGTGCTAGGGTTATTG

CTCAGACCACTGTAGATGCAAAAATCCATGCCAATTTTGAGGAGTCTGGTAGTTCCTTCGACTACTCCAG

TTCGGTACGTGCCTCCGGTACGGCTGATGCAGACCATCAACCAAAGTCCAACAAAGTAACAACAGCTTAC

CTGCATCACATACAGAGAGGCAAGCTGATCCAGCCTTTTGGTTGCTTGCTGGCCTTAGATGAGAAAACAT

GCAAGGTCATTGCATACAGTGAGAATGCACCTGAAATGCTCACCATGGTGAGCCATGCTGTCCCAAGTGT

TGGTGAACACCCTGCCCTTGGCATTGACACTGACATAAGAACTATTTTCACCGCGCCAAGTGCTTCTGCA

TTACAGAAGGCGCTGGGATTTGCCGAGGTTACACTTCTTAACCCCATCCTTGTTCATTGCAAGACTTCTG

GGAAACCCTTTTATGCAATCATCCATCGTGTCACGGGTAGTTTGATAATTGACTTTGAGCCGGTCAAGCC

GTATGAAGTTCCCATGACTGCAGCAGGTGCTTTGCAATCTTACAAGCTTGCTGCCAAAGCAATTACCCGG

TTGCAATCTTTGCCTAGTGGGAGCATGGAAAGGCTTTGTGATACAATGGTTCAAGAAGTTTTTGAACTCA

CAGGTTATGATAGGGTGATGGCTTATAAATTTCATGAGGATGATCATGGAGAGGTGATTGCTGAGATAAC

AAAGCCCGGGCTAGAGCCATATCTGGGTTTACACTATCCAGCCACTGATATTCCTCAGGCTTCACGCTTT

TTATTTATGAAGAACAAGGTCCGGATGATAGTTGATTGTCATGCAAAACAAGTGAAGGTTCTTATAGATG

AAAAACTCCCATTTGATTTGACTTTGTGTGGTTCAACCTTAAGGGCTCCTCACAGTTGCCATTTGCAATA

CATGGCAAACATGGATTCAATTGCTTCCCTGGTTATGGCAGTTGTAGTCAATGACAACGATGAAGACGGG

GATGGTTCTGATTCAGTTCAGCCACAGAAGAGAAAAAGACTCTGGGGTTTGGTAGTTTGCCACAACACTT

CTCCTAGATTTGTTCCTTTTCCTCTAAGGTATGCTTGTGAATTTCTGGCTCAAGTGTTTGCCATCCATGT

GAACAAAGAAATAGAATTAGAATGTCAGATTCTTGAGAAGAATATCCTGCGCACCCAGACACTCTTGTGT

GATATGCTGATGCGAGATGCGCCCCTAGGCATTCTAACACAGAGTCCAAATTTAATGGATCTAGTGAAAT

GTGATGGCGCTGCCCTCTTGTATAAAAACAAGGTATGGATGTTAGGAGTAACACCTAGTGAACTCCATAT

AAGAGACATAGCTTCATGGCTGTCTAAGTACCACACAGATTCCACAGGTTTGAGTACAGATAGCTTGTCT

GATGCAGGGTTCCCAGGGGCCCTTTCTCTTGGGGATCTTGTGTGTGGAATGGCAGCTGTTAGAATAACTC

CGAAAGACGTAGTATTCTGGTTTCGGTCGCACACTGCTGCAGAAATCCGATGGGGTGGTGCAAAGCATGA

ACCTGGAGAACAGGATGATGGTAAGAAGATGCATCCAAGATCATCATTCAAGGCTTTCCTTGAAGTTGTG

AGGGCAAGGAGCTCACCATGGAAGGACTATGAAATGGATGCTATTCATTCATTGCAATTAATACTGAGGA

ATGCATTCAAAGATACAGATAGTATGGATATAAACACAACTGCAATAGACACAAGATTAAGTGATCTGAA

AATTGAAGGGATGCAGGAACTGGAAGCAGTGACAAGTGAGATGGTTAGGTTAATTGAAACTGCAACAGTG

CCTATTTTGGCAGTCGATATTGATGGGCTGGTCAATGGGTGGAATATAAAAATTGCTGAGTTGACAGGTC

TTCCGGTTGGTGAAGCTATTGGAAAGCATCTACTCACACTTGTTGAGGATTGTTCAACTGATAGAGTAAA

GAAGATGCTTGACTTGGCACTTTCGGGTATGTCTTTTTTCTTTCGTAATCTTATCCTTATATAAGCACTT

CTCTTTTCTCTGTTTATCAAGCCTTAGCATTCATGATCAGAAGTTACAGATGTGTGTGTGTGTGTGACTA

TTTAATTTAATCAACTTTGCTAACCCATCCATGAATTTTGTATTGATGAAGAATTGTGAGAAGTATGTCT

TGTTGACAATTTCATTGAATTCTGTGGCCCTACAATACTTGAGTAGGAAAGGAACACTCAAGGAGTTGTG

TGGTCTTCTTTTATTTTGGGGGGTCTTTGTGTGATTTAATCCACTAGGCATGATTTGCAATTGGAATTAT

GACTGTTTAATACATATGGCTGAACCTCATTTAATAGGAGAATGTTTTTGTTCCTGTTGTGCGATGGTTG

TGGTGGCAATGGAAAGTGTTGGTGCAATTTCTCTAACACAATGGGATAACATTCTTCAAACCTGCTTTTG

ATTTCCAAATTGTTGATTTGTTTTGTTCTGTTGCAATGTAATTAGGTGAAGAAGAGAAGAACGTCCAATT

CGAGATCAAAACACACGGGTCTAAGATGGAATCTGGTCCTATTAGTTTGGTAGTTAATGCTTGTGCAAGC

AGGGATCTTCGAGAAAATGTTGTGGGGGTTTGTTTTGTGGCCCAAGATATTACTGCTCAGAAGACTGTAA

TGGACAAATTCACCCGAATTGAAGGCGATTACAAGGCAATTGTTCAGAACCCCAATCCACTAATCCCCCC

GATATTTGGCACAGATGAATTTGGCTGGTGTTGGGAGTGGAATCCAGCCATGACAAAGTTAACTGGATGG

AAGCGAGAAGAGGTGATGGATAAAATGCTTTTAGGAGAAGTTTTTGGGACACACATGGCTGCTTGTCGTC

TAAAGAATCAAGAAGCTTTTGTTAATTTTGGCATTGTGCTTAATAAAGCCATGACTGGTTCGGAAACGGA

GAAGGTCGGCTTCGGTTTCTTTGCTCGGAGTGGCAAGTATGTAGAATGCCTTCTTTCTGTGAGTAAGAAA

TTGGACGTAGAAGGTCTAGTTACAGGGGTCTTCTGCTTCTTGCAGCTAGCTAGCCCAGAGCTGCAACAAG

CATTACACATTCAGCGCCTATCCGAACAAACTGCTCTGAAAAGACTGAAGGCATTAACTTATATGAAAAG

GCAGATCAGGAATCCATTGTCTGGGATTGTATTTTCACGGAAAACGTTGGAGGGTACTGATTTGGGAATA

GAACAAAAACGACTTGTCCATACTAGTGCTCAGTGCCAGCGCCAGCTTAGCAAAATTCTCGATGACTCGG

ATCTTGACAGCATCATGGACGGGTACGATATCTGTCAATGTTGTTCTTTTTTCTCTTTGAATTCATGTCA

TGTGAAGCATTAAGTGTTGATCAATTATGTCGCATATTTAAGCCTAGAAGATCCATTTGGAAACTTCATT

GAAGCTCAATAATAGAAAGTTAACACCATGCTTGAGTCTTAACTCCACAAAACAAAGGTGACAACATGCT

TTGATATGCAGGTTTTTTTATTTTTTATTTTAAGAAGTTGTCTAAAGTTTAAGATGAAAAGAATAATAAT

AACTACAAGAAATTCTGTGTGTGCTATAGCGATAGCAATTTGATTTTCATTGTAGGGGTTAAGACTTTGA

CTTTCCTATTTTATCTAGTGAATACTTGCTGAATTTATATCCCGACTTTGAGAAGCTGATTCCTATAACA

AGGAATTTTTTTTCCTGGTAAAAGCATAGTTTTTTTCCAAACTAGAAATTGTATTTCCTTGTATCTGAAG

ATTAGCGTGCATCTAAAGCATGTGATCACTTGGTAACCTTTTATCAATTTTTGGGAGGAGAAGGGAATGT

AAGTTAGTTGGAGTTGACCAATTTGACATGCTTGAGGACATAAATAGGAATTAGGTTGTCTTAAGCGATT

TAAGTCTCCTTTTGTTAGTTTGCTGAAAGTCATGTTTTTGTTATTCTGATGGTTCATGTTATCTGCATCA

TGTTTTGGCAGGGAATACCAATAAAGCATATGTTTGATAATTAAAACTTTGCAACACGGAAACTGTGGAT

ATTTACTTCTGATTCGCACTGTGCTTTCCAAGTCAATCTATAGTGCTAGATTAGTTTGATTTTGTGAAAT

TATGAACGCAGGACCAGAGATACAAAAATCTTTGAGATTATTTGGCTTGATGTATTTGCTAACAGTTTAT

ATACTTGTTTTTTCTGAACAGCTACTTGGATCTTGAGATGGCTGAATTCACTCTCCAAGATGTACTGATT

ACCTCCCTAAGTCAAATCATGGCAAGGAGTAGTGCGAGAGGTATCCGAATAGTCAATGATGTCGCTGAGG

AGATCATGGTGGAAATCTTATATGGTGATAGTCTTAGGCTTCAGCAGGTCTTAGCCGACTTTTTACTGAT

TTCCATCAATTGTACACCAAATGGAGGTCAGGTTGTTGTTGCAGCCTCTCTAACCAAGGAACAGCTAGGG

AAATCTGTCCATCTTGCTAATTTGGAGCTCAGGTAAATTTGGAGTTTCATATTCATGCATTTAAAACTCC

CTATAAGTGGTTTATCTGGATCTACTTGTATTCCCGCTACCCTGTGGGGGTGAGAGCATAAGTTCGAATC

CTGGAAAAATAATTTCTTGGAGGGACAACTATAGTTTCCCGACCGGGGGAAAAACATCCAGGTTAAAGAC

AATAAAAAGTGATGAAATCTAATTGGATGCCCGTGTAAAAATCATTTACACTGTCGGTGTATAGTTAATC

TCATGAACTAATGTGTGATGTCAAGATAAATCCTTGTTCTCTTGTGCTAGGGAACTACCTTAGAAACAAA

AGAAACTTTGCATTATTTTTATCCTATGAAGAGGAGAAGAAACTGCATTGTGAATCTTATTGACAGCTAA

ATTTGGCTTTAAACTAATAAGTGGTTATTTTTTGCTATTTATGCAGCATAACACATGGCGGTAGCGGGGT

GCCGGAAGCATTGCTGAACCAGATGTTTGGAAATGATGGGCTAGAATCAGAGGAGGGTATTAGCCTGCTC

ATCAGCAGAAAGCTGCTAAAGCTCATGAGTGGAGATGTTCGTTATCTAAGGGAAGCAGGCAAATCATCGT

TTATCCTATCGGTTGAACTTGCTGCAGCACATAAGTTGAAAGCTTAA

>CP023120.1:5563092-5565201 Lupinus angustifolius cultivar Tanjil chromosome LG-08

AAAACAAAAAGATCTTATGTAAGGATAAGATGGAGAAAGAAAAAAATACATACCCTGCAGTGCCATGTCAAGCATCTTCTTGACTCTATCAATCGAAGAATCCTCAACAAGTGTGAGTAAATGCTTCCCAATAGCTTCACCAACTGGAAGACCTGTTAACTCAGCAATTTTTGTATTCCATCCATTGACTAGCCCATTAACATCAACTGACAAAATAGGCACTGTAGCTGTTTCAATCAACCTAACCATTTCGCTTGCCACTGCTTCCAGTTCCTGCATCCCTTCAATCTTCAAATCGCTTAGTCTTGTATCGATTGCACTTGTGTTTATATGCGTAGTCACTGTATCTCTGAATGCGTTTCTTAGTATTAGCTGCAATGAATGAATAGCATCCATTTCATAGTCCTTCCATGGTAAGCTCCTTGTCTTCACAACTTCAAGGAAAGCCTTGAATGATGATCTTGGATGCATCTTCCTACTATCATCGCTTTCACCAGGTTCATGCTTTGCACCACCCCAGCGGATTTCTGCAGCAGTGTGTGACCGAAACCAGAAAACTGTGTCTTTCGAAGATATTCTAACAGCTGCCATTCCACACACAATATCTCCAAGAGAAAGAGCCCCTGGGAATCCTGCATCTAACAAGCTATCTGTACTCAAACCTGTGGAATCCATATGATGCTCAGATAGCCATAAAGCTATATCTCTTATCTCGGATTCGCTCGGTGTTACTCCTAATCTCCATACTTTGTTTTTATAAAATAGTGCTGCTCCATCACATTTTACTAGATCCATTATATTTGGGCTTTGTGATACAATACCTAAGGGTGCATCTCGCATCAGCATATCGCACAAGAGTGTCTGAGTGCGCAGGATATTCTTCTCAATAATCTGAAGTTCTAACTCTATTTCTTTGTTCACATGGATGGCAAATACTTGAGCCAGAAACTCACAAGCATACCTTAGAGGAAATGGAACAAACCTGGGAGTAGTGTTATGGCAAACTACCAAACCCCATAGCCTCTTTCTCTTCTGAGGCTGGACAGCATCGGAGCTATCACCATCTTCATCGTTGTCATTGACTACTACTGCCATAACAAGGGAAGCAATTGAATTCATGTTTGCCATGTATTGCAAATGGCAACTATGAGGAGCCCTTAAAGTTGAACCACACAAAGTCAAATCAATAGGGAGCTTCTCATCTTGAAGAACCCTCACATGTCTTGCATGACAATCTACTATCATGCGAACCTTGTTTTTCATGAACAAAAAGCGTGAAGCCTGGGGAATATCGGTGGCTGGATAATGTAAACCCAGATATGGCTCTAGGCCTGGCTTTGCTATCTCAGCAATCACCTCACCGTGATCATCCTCATGAAATTTATAAGCCATAACCCTGTCATAACCTGTGAGTTCAAAAACTTCTTGAACCATTGTATCACAAAGCTTTTCCATTTTCCCACTAGGCAAAGATTGCAGACGTGTAATTGCTTTGGCAGCCAGCTTGTAAGATTGCAAGGCACCTGCTGCAGTCATGGGAACTTCATAAGGCTTGACTGGCTCAAAGTCTATGATCAAACTAGCAGTCACACGGTGGATAATTGCGTAAAATGGTTTCCCAGAAGTCTTGCAATGAACTAGGATTGGGTTAAGAAGTGAAACCTCTCCAAATCCTAGTGCTTTTTGCAATGCAGAAGCACTTGGTGCAGTGAAAATAGTTCTTATGTCAGTGCCAATCCCAAGGGCAGGGTGATCACCAACACTAGGGACAGCATGACTCATCATTGTCAGCATTTCAGGTGCATTCTCACTGTAAGCAATGACCTTGCATGTTTTCTCATCTAAAGCTAGCAAGCAGCCAAAAGGCTGGATCTGCTTGCCTTTCTGTATGTGATGGAGATAAGCTGTGGTTACTTTGTCAGACCTTGGTTGATGGTCTCCATTAACCGTATCCGAGACGCGCACTGAACTGGAGTAGTCAAAGGAACAACCTGACTCCTCAAAGTTTGCTTGGAGTTTTGCATCTACTGTTGTCTGAGCAATAATCCTAGCACTATTTCTTGATCTCCCTGAATTATTGGACGATTGGCTAGGCCTCGAGGAAGACATT

>AY688953.1 Pisum sativum phytochrome A apoprotein (PHYA) gene, complete cds

TCAAATTCATGACATCCGACTTATTTATCTTTAAAAAATAAAATCTCGGCCACTAAACTACTTGATAAAA

AAATTTATTATATCTATATAATTATATTTAAAAATGAGCGAATAGTGATAAAATTTGTCTAAACTATTAT

AAAAATAGTAAATATTAATTAAAATATTATAATTTTTAATTCTCAACTTATAAATATTAATAACTTATTT

ATTATAAATCATTTTTTACTTTTTATATATATTAAAAAATATAATAATTATGGTATAAAAATGAGTAAAA

TGTAATGGATTATGAGAAAATATATTATTTAAAAAGTAAAGTGATAATTGAATATGTGATTCTTACAAAA

TATGACTAACCAATAAAATAAGAAGAAACCTTAGAGGATAGGTAACCAAGTAAATATAATATAGTATAAA

TCTGGTAACACTAACAAGTGTTTCCCTGTGAGTCAGTAAGGTTACATGACAGTGGTACGTACGAGGCATG

GGCTCATTTGTTAAATACAATTTCATTGTCAGACATGAGCTGTGATCTAAAAGATGTGAAATCGATAGAG

AAAAACTCTCCACCAATTAAAATCAAACAATACGATGACATGGCATCACTATCATTCAGCCGCATCACGT

AAAAACTTTCCGCATCCCTCGCACGTGACACCCATAACTCACTACTTTCCCATTATCCAACGCATGCCTC

TTGGACACTCGTCCAATCAAGTTCCTCCACCTCACCCCTTTTTTTCCCCACCCCTATTCTATACTTAGCA

TGTTTAATTATTGAATTAGGATTCAATAATTATCCATTTATTAAATTAAATTAAAATTAATAGCCGACAT

GGAAAGTGAAGTTGAAAAAGAAAGTCATGTTTTCTCAGTAACAGTCATGTTTTCTGTTTAATTATGTGTC

TGTTTTATCATTCATCATTTAACTGTCCTTTTTTTCTTATCTATTTTAGGAACAAAACCAAACCCACTTG

CCCAAATTAGTTTTATTTTATTTTATTTTAAATTATAAATATATAATAATAAATAAACAGCACCAACACC

AAGTAGATGCTGTTGCTGAATGAATCTATCTAAGAGTTGGATTGGATTGGATGATGATTTGATTTTTTAT

TTTTATAATATTCTTTTATTTATCTTTATTCATTTCATTCCTTTCTTTCTCCCACACCTTCACATGGGAA

CACTATAAAATTCATATATCTTTATAGCATATGATGGCGGTCCCTTCTGTTCTCAGTCACAATTCTGTGT

TGAATAATAATAATACTGAGCTTCCAGTTGTGGACTAAGGGCTCCATCTAGGTAGGAGACAAACAAGGCC

GGTGATCCAGATTCACCATCTTATTTACTGTTGTTGCTGCTGCAGGTGCACGGTATGGTATGCTGGTCTC

CCACTAATGGTTATTTTAACCCTTACCTTTAATACCTTTCTTTCTTCAACTTGTACTTGAACTTGAACTT

TCACAACAGCCAAGTTTTTCCCTTTCAAGGTTAGTTAGTACTATTTTGCTTCTTGTGTTTATATTTCATC

ATGTGTCAGGGTTGTTGATTTGATTGTTTTCTATGATTGGGTTTTACTTCATAGATTGTGACTAGTTATT

TTGGTAAAGCCTTTGATAATTTTGTCGTATTGATGATAGAGTTCAATAGTTTTGTAACATCATCGTATAT

GGTTTTGATTGACTTGGTTATCTTGTTTTTTTTACTGTTGTAAATGTAGTTTGTGTTAAAATGTCAACCA

CGAGGCCTAGCCAATCGTCCAACAATTCGGGGAGGTCAAGAAATAGTGCTAGGATTATTGCTCAGACGAC

TGTGGATGCAAAGCTTCATGCAACTTTTGAGGAGTCCGGTAGTTCGTTTGACTACTCGAGTTCGGTGCGT

GTTTCTGGCTCGGTGGATGGAGATCAACAACCGAGGTCCAACAAAGTGACGACGGCTTACCTCAATCATA

TACAGAGAGGTAAGCAGATCCAGCCTTTCGGGTGCTTGCTAGCTTTAGATGAGAAAACGTGCAAGGTTGT

TGCGTATAGTGAGAATGCGCCTGAGATGCTGACTATGGTGAGTCATGCTGTTCCTAGCGTTGGTGACCAT

CCTGCTCTTGGCATTGGAACTGACATAAGGACTGTTTTCACTGCGCCGAGTGCTTCTGCCTTGCAGAAGG

CGCTAGGGTTTGCGGAGGTTTCACTTCTTAACCCGATTCTTGTTCATTGCAAGACTTCTGGGAAGCCGTT

TTACGCGATCATTCATCGTGTTACTGGTAGTTTGATCATTGACTTTGAGCCGGTGAAGCCTTATGAAGTT

CCCATGACTGCTGCGGGTGCCTTGCAATCTTACAAACTTGCTGCTAAAGCAATTACAAGATTGCAATCTT

TGGCTAGTGGCAGCATGGAAAGGCTTTGTGATACCATGGTTCAAGAAGTTTTTGAACTAACGGGTTATGA

CAGGGTGATGGCTTATAAATTTCACGAGGATGATCACGGGGAGGTGATTGCTGAGATAGCAAAGCCAGGC

CTAGAGCCATATCTAGGTCTGCACTATCCGGCGACAGATATTCCCCAGGCTGCGCGGTTTCTATTTATGA

AGAACAAGGTCCGTATGATAGTTGATTGTAATGCAAAACATGTGAAGGTTCTTCAAGACGAAAAACTCCC

ATTTGATTTGACTCTGTGCGGTTCGACCTTGAGAGCTCCACATAGTTGCCATTTGCAGTACATGGCTAAC

ATGGATTCAATTGCTTCGTTGGTTATGGCAGTTGTCGTCAATGACAGCGATGAAGATGGAGATAGCGCTG

ACGCAGTTCTCCCACAAAAGAAAAAGAGACTTTGGGGTTTGGTAGTTTGTCATAACACTACTCCAAGGTT

TGTTCCTTTTCCTCTAAGGTATGCTTGTGAGTTTCTGGCTCAAGTGTTTGCCATCCATGTGAACAAAGAA

ATAGAGTTAGAATATCAGATTCTTGAGAAGAATATCCTGCGCACGCAGACGCTGTTGTGTGATATGTTGA

TGCGAGATGCACCCTTAGGTATTGTATCACAAAGCCCTAATATAATGGATCTAGTGAAATGTGATGGGGC

TGCACTCTTTTATAGAAACAAGTTATGGTTATTAGGAGCGACACCGACTGAATCTCAATTAAGAGAGATA

GCTTTATGGATGTCTGAGTATCATACAGATTCAACAGGTTTGAGTACAGACAGCTTGTCGGATGCAGGGT

TTCCAGGGGCTCTTTCTCTTAGTGATACTGTATGTGGAATGGCAGCTGTTAGAATAACTTCAAAAGACAT

AGTTTTCTGGTTTAGGTCGCACACTGCTGCAGAAATCCGATGGGGTGGTGCAAAGCATGAACCGGGCGAC

CAAGACGATGGTAGGAAGATGCATCCAAGATCATCATTCAAGGCTTTCCTTGAAGTTGTGAAAGCCAGAA

GCGTGCCGTGGAAAGACTTTGAAATGGATGCTATTCATTCGTTGCAGTTAATACTGAGAAATGCGTCCAA

AGATACAGATATTATAGATTTGAATACGAAGGCAATCAATACAAGACTAAATGATTTGAAGATTGAAGGG

ATGCAGGAATTGGAAGCAGTGACAAGTGAGATGGTTAGATTAATTGAAACAGCAACAGTGCCTATTTTGG

CAGTGGATGTTGATGGGACGGTCAACGGATGGAATATAAAAATCGCCGAGTTGACAGGTCTTCCGGTTGG

CGAAGCTATTGGAAAACATCTACTCACCCTGGTTGAGGATTCTTCAACTGATATAGTCAAGAAGATGCTC

AACTTGGCACTGCAGGGTATGCCTTTTTTCCTTTTCCATCTTATTATCCTTTACTTAAGACTCTAACTGT

TGATTTTGTTCTCTTATAATGTAATCAGGTGAAGAAGAGAAGAATGTTCAATTCGAGATAAAAACACATG

GGGATCAGGTGGAATCCGGTCCTATTAGTTTGATAGTTAACGCATGTGCAAGCAAGGATCTTCGTGAAAA

TGTAGTGGGGGTTTGTTTTGTGGCCCAAGATATAACTGCTCAGAAGACTGTCATGGACAAATTCACCCGA

ATCGAAGGCGATTACAAAGCAATTGTGCAGAACCCAAATCAGTTGATCCCTCCTATATTCGGTACAGATG

AATTTGGCTGGTGTTGTGAGTGGAATGCAGCTATGATTAAGTTAACCGGATGGAAGCGCGAGGAGGTGAT

GGATAAAATGCTTCTCGGAGAGGTTTTCGGAACTCAAATGTCTTGTTGTCGTCTAAAGAATCAAGAAGCT

TTTGTTAATTTCGGCATTGTACTTAATAAAGCCATGACCGGTTTGGAAACAGAAAAGGTCCCTTTTGGCT

TCTTCTCTCGAAAAGGCAAGTATGTAGAGTGCCTACTCTCGGTGAGTAAGAAAATCGACGCAGAGGGCCT

AGTTACCGGAGTCTTCTGTTTCTTGCAGTTAGCTAGCCCTGAGCTGCAACAAGCATTACATATTCAGCGC

CTGTCCGAACAAACCGCTCTCAAGAGACTGAAAGTACTAACTTACATGAAAAGGCAGATCAGGAATCCGT

TGGCTGGGATCGTGTTTTCCAGTAAAATGCTGGAGGGTACTGACTTGGAAACCGAACAAAAGCGAATCGT

GAACACTAGTTCTCAGTGCCAACGCCAGCTTAGCAAAATTCTTGACGACTCTGATCTCGACGGCATCATT

GATGGGTATGATATCAGTAAATATATTGTTCTTTTAACATTTTTTTTTACCCTTACTCACATTCATGTGT

GAAATTTTGACAGGTACTTGGATCTTGAGATGGCTGAATTCACTTTACATGAGGTACTGGTTACCTCTCT

TAGTCAAGTCATGAATAGGAGTAACACAAAGGGTATCCGTATAGCCAACGATGTTGCGGAGCATATCGCG

AGGGAAACCTTGTATGGTGATAGTCTTAGGCTTCAGCAGGTCCTAGCTGACTTTTTACTAATTTCCATCA

ATTCCACACCTAATGGAGGCCAGGTTGTTATAGCAGCCTCCTTAACTAAAGAACAGTTGGGAAAATCTGT

CCATCTTGTTAACTTGGAGCTCAGGTAACTGTTCCATATTCACGCATGTATCGATTTTCAGTATCCGATG

ATTTAAGCCTCGGTAATAATCTTAGCTTTGGTGAAACTGCTAACTGCAATAAACTAATATCTGTATATAT

TTTGTTTGCGATTTGTGTAGCATAACACACGGTGGTAGTGGCGTGCCAGAAGCGGCGCTGAACCAGATGT

TTGGAAATAATGTGCTAGAATCTGAGGAGGGTATTAGCCTACACATCAGTCGGAAGTTGTTAAAGCTTAT

GAATGGAGATGTTCGTTATTTAAAAGAAGCAGGAAAATCATCGTTTATTCTATCTGTTGAACTTGCAGCA

GCTCATAAGTTGAAAGGTTGAAATTTTGGAAAAATAATAAAAGTGAACACGGCATGCTATTGTTCAATGT

AACTCTTATTTTGTTTCATGTTTCTTTAACTTGTTGTAACGTATGTTGTAGATATGTGTATTAGTGTCTC

TTTCACACTCGTGGCCTATTCTTATTTCTTAGTCATGCTTTATGTTTTTTGTTTTATTTTTAGAGCAATT

AATGGCATGTGTGGGGATGAACGCAGTTGGGGAAAACAGTTAATAACAACTATGCTATGACTTACTGTAC

TAGTTTCTCAATATCTTCTTTCTCTGCTTTATTCAGTCCTGTCACCCATACTATATATAGTACACATATA

TAGTACAATCTAGGAAGAGTTTAAAAGAGTACATTCCACCCAATTACAAGTTCTGAATAACAAACTAGTG

ACTCTTTTAACAATCTTGATTACTCACAGCACTGACAAATAAAAGCTTCCTACCCCAGATTTGAGCTACT

TGTACCTCATTGCATTTCTCTTTGCCTCGCAATTGTGGAATGGACTCCTCCTCTTCTGACCCTATCCTTT

CCAAATCTTCCTCTTCATAGTTCATGACATCCCGAGTCCATCAAAACTGACCTTGTCCTTAAGGTCAAAG

GTCGGGTAAATAGCATATATGTCTCTCAAAACTTGGTTTTGATCATTAATAGTGTCTTTCTTCCAATCCA

TCACAGCTAACGGCTTGATGTGAGGCCGGTATCCAAGAGGCATATCCAAGGTTTTCATAGTTGTATCAAA

ATAGGGTTTCAAGTTTCAACTGTGATATATGGAACATAAGACGAATCCTATGGGTGCATGGGAATTT

>U83267.1 Pongamia pinnata phytochrome A-like (PHYA-like) gene, exon 1, partial cds

AGGGTTCGTATGATAGTTGATTGTCGCGCAAAGCATCTGAAGGTTCTTCAAGACAAAAAGGTTCCATTTG

ATTTAACTTTGTGTGGATCAACCTTAAGGGCTCCTCATAGTTGCCATTTGCAGTACATGGAGAACATGAA

TTGTAGTGCTTCCTTGGTTATGGCAGTTGTGGTCAACGACAATGATGAAGACGGAGATAGTGATGCTGTT

GAGCCACAGAAGAGAAAAAGACTCTGGGGTTTAGTAGTTTGCCATAACACTACTCCCAGGTTTGTTCCTT

TTCCTCTTAGGTATGCCTGTGAATTCCTTGCTCAAGTATTTGCCATCCATGCGAACAGAGAACTAGAGCT

AGAATATCAGATTGTTGAGAAGAATATCCTGCGGACTCAAACACTGTTGTGTGATATGCTTATGCGAGAT

GCTCCACTAGGCATTGTAACACAGAGGCCTAACATAATGGACCTAGTCAAATGC

>XM_017563899.1 PREDICTED: Vigna angularis phytochrome A-like (LOC108329605), mRNA

AGAGAGAGAGAGAGAGAGAGAGAGAGAAAGAGAGAGAGTAGTCTTAAATTGTTATTTTTACTATGGCATG

GCATTATCTACTGTGTCTCAGTTCTAACTGCCGAGTTCTGTGAGCTTGGAGGGGTTCCCACTTTCCACTT

GTGTCCCAAAGGTCGGTGGTTAAGATTTTCTTCTTCTTCTTCTTGTTCATCTACAGATGCAGCTAAACAG

TAAGTAGCTATCGGGGATTTATATTCCCTAGTTGCTTTCTGCTTCTATTCAGACCTTCTCTCTGCTCATT

GGTTGGAATCAGATTTTTCCTTGCAAGTTGAAGTGACAATGTCCTCCTCAAGGCCTAGTCAATCGTCCAG

CAATAATTCTGGCAGATCAAGATCATCAAGACGCAGTGCTAGGGTTCTTGCTCAGACAACTTTAGACGCA

AAACTGCGTGCAACTTTCGAGGAATCCGGTAGTTCTTTTAACTACTCCAATTCAGTGAGATTGTCCCCTG

GCACAGGCACTGCCAGTGGTGATCATCAACCGAGGTCTGATGAAGTAACAACTGCTTACCTCCATCAGTT

ACAGAAAAGCAAGCTTATCCAACCATTTGGGTGCTTGCTAGCGTTAGATGAGAAAACATTTAGGGTCATT

GCTTACAGTGAGAATGCACCTGAAATGCTCACCATGGTTAGCCATGCTGTCCCCAGTGTTGGTGACCACC

CTGCTCTGGGAATTGGCACTGACATCAGAACTATTTTCACTTCCCCGAGTTCTACTTCTATTCAGAAGGC

ACTGGGATTTGGGGAGGTTTCACTTCTTAACCCCATTCTAGTTCATTGCAAGTCCTCTGGGAAGCCCTTT

TATGCAATTATCCATCGTGTTACCGGCAGTGTGATCATTGATTTTGAGCCAGTCAAGCCTCATGAAGTTC

CCATGACTGCAGCAGGTGCCCTGCAGTCCTATAAATTTGCAGCAAAAGCAATAACTAGATTGCAATCGTT

GCCTAGTGGGAACATGGACACTCTGTGTGACACAATGGTTCGAGAGGTTTTTGAACTAACAGGTTATGAT

AGAGTGATGGCTTATAAATTTCATGAGGATGATCACGGGGAAGTGATTGCTGAAGTGAAAAGGCCAGACC

TAGAGCCGTATCTGGGATTGCACTACCCAGCAACTGATATTCCTCAGGCTACACGTTTTTTGTTTATGAA

GAACAAGGTGCGTATGATAGTTGATTGTCGTGCAAAGCATGTGAAGGTGCTTCAAGACAAAAAAATTCCA

TTTGAATTAACTCTGTGTGGATCAACCTTGAGGGCTGCTCACAGTTGTCACTTGCAATACATGCAAAATA

TGAATTCTAGTGCTTCCTTGGTTATGGCCGTTGTGGTCAATGACAATGATGAAGATGGGGATAGTTCTGA

TGCTGTTCAGCCACAGAAGAGAAAGAGATTATGGGGTTTAGTAGTTTGCCATCACTCCACTCCCAGGTTT

GTTCCTTTCCCTCTCAGGTATGCTTGCGAATTCCTGGCTCAGGTATTTGCCATCCATGTGAACAAAGAAC

TTGAGATAGAGTATCAGATTATTGAGAAGAATATCCTGAGGACTCAAACACTCTTGTGTGATATGCTGAT

GCGAGATGCACCCCTAGGTATTGTATCACAGAGTCCTAACATTATGGACCTTGTTAAGTGCGATGGTGCA

GCACTGTTGTATAAAAACAAGGTATGGAGATTAGGGGTAACACCAAGTGAATCCCAGGTAAGAGAGATAG

CTTTGTGGCTCTCTGAATGTCACAGGGATTCCACAGGTTTGAGTACAGATAGCTTGTCTGATGCAGGCTT

CCCAGGGACTGCTACTCTTGGTGATATAACTTGTGGAATGGCAGCTGTCAGAATATCTTCCAAAGATATA

GTTTTCTGGTTCCGATCTCACACAGCCGCAGAAATTCGATGGGGTGGTGCAAAGCATGAGCCTGGTGAAA

GGGATGATGGTAGCAGGATGCATCCAAGATCTTCATTCAAGGCTTTCCTTGAAGTTGTGAAGACAAGGAG

TTTGCCCTGGAAGGATTATGAAATGGACGTCATTCATTCATTGCAACTAATACTGAGAAATGCATTCAAA

GACAACGAGAGTATGGAGATAAGCACATATGCTATCAATACAAGATTAGACGATTTGAAAATTGAAGGGA

TGCAAGAACTGGAAGCAGTGACTAGTGAGATGGTAAGGTTAATTGAAACAGCAACAGTACCAATTTTGGC

TGTTGATGTCAATGGAATGGTCAACGGGTGGAATACAAAAATTGCTGAGTTGACATGTCTTCCAGTTGAA

CAAGCTATTGGGAAGCATTTACTCACTCTTGTTGAAGATTTTTCAGTAGATAGAGTCAAGAATATGTTGG

ACATGGCACTGCAGGGTGAGGAAGAGAAAAGTGTGCAATTTGAGATCAAAACACACGATTTGAAGATTGA

TTCTGGTCCTATCAGCTTGGTGGTCAATGCTTGTGCAAGCAGGGATCTTCAAGATAATGTTGTGGGGGTT

TGTTTTGTGGCCCAAGATATAACTGCTCAGAAGACAGTGATGGACAAATTCACCCGAATTGAAGGCGACT

ACAAGGCAATTGTGCAGAACCCAAACCCATTGATCCCTCCAATATTTGGCACAGATGAATTTGGGTGGTG

TTCTGAATGGAATTCAGCTATGACAAAGTTAACTGGATGGAAGCGAGAGGAGGTGATGGATAAAATGCTT

TTAGGGGAGGTTTTTGGTACACATATAGCTTGTTGTCGTCTAAGGAATCAGGAAGCTGTTGTTAACTTCA

GCATTGTACTTAACAAAGCCATGGCTGGTTTGGAAACGGAGAAGATTCCTTTTAGTTTCATTACTCGTGA

TGGGAAACACGTAGAATGTCTGCTTTCTGTGAGTAAGAAGTTGGATGCAGAGGGTGTAGTTACTGGAGTC

TTCTGCTTCTTGCAACTAGCTAGTCCAGAGCTGCAACAAGCATTACACATTCAGCACCTATCTGAACAAA

CTGCATCCAAAAGACTGAAAGCTTTAACTTATTTGAAAAGGCAAATTCGGAGTCCTTTATACGGGATTGT

TTTCACCCGGAAATTGTTAGAAGGTACTCAGTTGGGACCTGAACAAATACAATTTCTGCAAACGGGTACT

CGGTGTCAACGCCAACTTAGCAAAGTCCTGGATGACTCAGATCTTGACAGCATCATTGATGGGTATCTGG

ATTTGGAGATGCTTGAATTTACTCTGCATGAAGTGTTAGTTGCCTCCTTAAGTCAAGCCATGACAAAAGG

TAATGCAAAAGGTGTCCGAGTGGTCAATGATGTTGAAGAGCAGATCACAACAGAAACCTTATACGGTGAT

GGTCTCAGGCTCCAGCAGGTCTTAGCCGACTTTTTATTGGTTTCCATCAATTTCACACCAACTGGAGGTC

AGGTTGTTGTGGCAGCCTCATTAACAAAACAGCAGTTAGGGAAACTAGTTCATCTTGCTAATTTGGAGCT

CAGCATAACACATGATGGTTTTGGGGTTCCAGAAACACTGCTGAACCAAATGTTTGGACGTGATGGTGAT

GAATCTGAGGAAGGTATCAGCATGCTGATTAGCAGAAAGCTGCTAAAGCTGATGAATGGAGAAGTACGTT

ATATAAGGGAAGCAGGCAATTCATCTTTCATCTTATCTGTTGAACTTGCCGCAGCTCATAAATCCAACAC

TTAAAATTTTGGAAATAATTAAATCAAACCATATTTTTTGTACATCCGAAAGTGAAGGGTGCAAGAAATT

TGCATTCCTTGTTCAATATTTAACTCTTGTTTTTATGTACCTACCTTCTTGTAAGGTGATAATGTAGATA

TGTATAACTAACGATTCACGCTCTCTCCTCAAGTGCTCTCATTACAGCAATTATGCCTCATTCTCAACAG

AATGAAACATAAATAAAAGCTAACCAGGAACATTCCGCATCA

>CP039350.1:48434274-48436821 Vigna unguiculata cultivar Xiabao 2 chromosome Vu01

CTTTCAAAGTTTGTGAATGTTCTAGTGTTGAAAAATTCACTTTCTCTTGTTGAATTGTGATAGTGAATAAGGATGATGTCATTTGACAAGGTCCATAGTAGCTTTGCATCTGTATCAGAGCAATCAAACAGATTCCGTTCTTCCTTGCGAGATATTAACTTAGTGATTCTGTGCTTGTGGTTTATTTCAAGTGCAGTTGAAGTGACAATGTCCTCCTCAAGGCCTAGCCAATCATCCAGCAATAATTCTGGCAGATCAAAATCATCAAGACGCAGTGCTAGGGTTCTTGCTCAGACAACTTTAGACGCAAAGCTGCATGCAACTTTCGAGGAATCGGGTAGTTCTTTTAACTACTCCAATTCGGTGAGATTGTCTCCTGGCACAGGCACTGCCAGTGGTGATCATCAATCAAGGTCTGATGAAGTAACAACTGCGTACCTGCATCAGTTACAGAAAAGCAAGCTTATCCAACCATTTGGGTGCTTGCTAGCGTTAGATGAGAAAACATGTAAGGTCATTGCTTACAGTGAGAATGCACCCGAAATGCTCACCATGGTTAGCCATGCTGTCCCAAGTGTTGGTGAACACCCTGCTCTTGGCATTGGCACTGACATAAGAACTATTTTCACTGCCCCGAGTTCTACTTCTATTCAGAAGGCACTAGGATTTGGGGAGGTTTCACTTCTTAACCCCATTCTAGTTCATTGCAAGTCCTCTGGGAAGCCCTTTTATGCAATTATCCATCGTGTTACCGGCAGTGTGATCATTGATTTTGAACCGGTGAAGCCTCATGAAGTTCCCATGACTGCGGCAGGTGCCCTGCAATCCTATAAGTTTGCAGCAAAAGCAATAACTAGACTGCAATCCTTGCCTAGTGGGAACATGGACACTCTGTGTGACACAATGGTTCGAGAGGTTTTTGAACTAACAGGTTATGATAGAGTGATGGCTTATAAATTTCATGAGGATGATCACGGGGAAGTGATTGCAGAAGTGAAAAGGCCAGACATGGAGCCGTATCTGGGGTTGCACTACCCAGCAACTGATATTCCTCAGGCTACACGTTTTTTGTTTATGAAGAACAAGGTGCGTATGATAGTTGATTGTCGTGCAAAGCATGTGAAGGTTCTTCAAGACAAAAGAATTCCATTTGAGTTAACTCTGTGTGGATCAACCTTGAGGGCTGCTCATAGTTGTCACTTGCAATACATGCAAAACATGAATGCTAGTGCTTCCTTGGTTATGGCGGTTGTGGTCAATGACAATGATGAAGATGGGGATAGTTCTGATGCTGTTCAGCCACAGAAGAGAAAGAGACTATGGGGTTTAGTAGTTTGCCATCACTCTACTCCCAGGTTTGTTCCTTTCCCTCTGAGGTATGCTTGCGAATTCCTGGCTCAGGTATTTGCCATCCATGTGAACAAAGAACTTGAGATAGAGTATCAGATTATTGAGAAGAATATCCTGAGGACTCAAACACTCTTGTGTGATATGCTGATGCGAGATGCACCCCTAGGTATTGTATCACAGAGTCCTAACATTATGGATCTTGTTAAGTGCGATGGTGCAGCACTGTTGTATAAAAACAAGTTATGGAGATTAGGGGTAACGCCAAGTGAATCCCAGATAAGAGAGATAGCTTTGTGGCTCTCTGAATGTCACAGGGATTCCACTGGTTTGAGTACAGATAGCTTGTCTGAGGCAGGCTTCCCAGGGGCTGCTACTCTTGGTGATATAACTTGTGGAATGGCAGCTGTCAGAATATCTTCCAAAGACATAGTTTTCTGGTTCAGATCTCACACAGCCGCCGAAATTCGATGGGGTGGTGCAAAGCATGAGCCTGGTGAAAGGGATGATGGTAGCAGGATGCATCCAAGATCTTCATTCAAGGCTTTCCTTGAAGTTGTGAAGACAAGGAGTTTGCCCTGGAAGGACTATGAAATGGATGTCATTCATTCATTGCAGCTAATACTGAGAAATGCATTCAAAGACAACGAGAGTATGGAGATAAGCACATATGCTATCAATACAAGATTGGGTGATTTGAAAATTGAAGGGATGCAAGAACTGGAAGCAGTGACTAGTGAGATGGTAAGGTTAATTGAAACAGCAACAGTACCAATTTTGGCTGTTGATGTCAACGGAATGGTCAACGGATGGAATACAAAAATTGCCGAGTTGACATGTCTTCCAGTTGAGCAAGCTATTGGGAAGCATTTACTCACTCTTGTTGAAGATTTTTCCGTAGATAGAGTGAAGAAGATGTTGGACATGGCACTGCAGGGTATGCCTTTTTCTTTTCTTTTCTTATTCTCATACACTTGTTTTAGGTCTTAGCATTCATGAGCAGAGACTTTGAAATGAGTGTGGGTATTTAGATGTTAGGTTGTTATCTTACCTAAAGTCACTCATGAATATTGCATTAGTGAAGAATTCTAAAAATTTTAATTTGTCTTGCTTCGAAGTTAAACTTAATTTTATCTGAACCTCCTATAATTTAACATGTTACAAAACATGATCAGGTGAGGAAGAGAAAAATGTGCAATTT

>XM_014658076.2 PREDICTED: Vigna radiata var. radiata phytochrome A (LOC106771983), transcript variant X3, mRNA

AGTTGAGTTCCAGGAAGAGGAAGGGGAGACATGGGTGTTGTTGCCCACACCTTTTTTCTTTATTTTAACC

CTTAACTTCCGTTATATCTTCATTGCTTCCTTCCTTTACTTCTTCTCACTCTTTCCCTTTCGAGTTTGGA

GTGAAAATGTCTTCCTCGAGGCCCAGCCAATCGTCCAGCAATTCAGGGAGATCAAGACATAGTGCTAGGG

TTCTTGCCCAGACAACTGTAGATGCAAAGCTTCATGCAACTTTTGAGGAGTCTGGTAGTTCCTTTGATTA

CTCCAGTTCGGTGCGAGTCTCTGGTACAGCTGATGGAGTCAATCAACCAAGGTCTGATAAAGTTACGACA

GCTTACCTCCATCACATACAGAGAGGAAAGATGATTCAGCCTTTTGGATGCTTGTTGGCTTTAGATGACA

AAACATGTAAGGTCATTGCATACAGTGAGAACGCTCCTGAAATGCTGACCATGGTTAGTCATGCTGTCCC

CAGTGTTGGTGACCACCCTGCCCTTGGCATTGGCACTGACATAAAAACTTTGTTCACAGCACCTAGTGCT

TCTGCATTGCAAAAGGCTCTGGGATTTGGGGAGGTTTCACTTCTTAACCCGATCCTTGTTCATTGCAAGA

CCTCTGGGAAGCCCTTTTATGCGATTATTCATCGTGTTACTGGCAGCTTGATCATTGACTTTGAGCCAGT

CAAGCCTTATGAAGTTCCCATGACTGCAGCTGGTGCCTTGCAGTCTTACAAGCTTGCTGCCAAAGCAATT

ACCCGGTTGCAATCTTTGCCTAGTGGAAGCATGGACAGATTATGTGATACAATGGTTCAAGAAGTTTTTG

AGCTCACAGGCTATGACAGGGTGATGGCTTATAAATTTCACGATGATGACCATGGGGAGGTGATTGCTGA

GATAACAAAGCCAGGCCTTGAGCCATATTTGGGTTTGCATTATCCAGCCACAGATATTCCCCAGGCTTCA

CGCTTTTTATTTATGAAGAACAAGGTCCGTGTGATAGTTGATTGTCATGCAAAACACGTTAAAGTTCTGC

AAGATGAAAAACTCCCATTTGATTTGACTTTATGTGGTTCCACCTTGAGGGCTCCCCATAGTTGTCATGC

TCAATACATGGCAAACATGGATTCAATTGCCTCCCTGGTCATGGCAGTTGTAGTCAATGACAACGAAGAA

GATGGTGATACTGATGCTGTTCAGCCACAAAAAAGGAAGAGACTTTGGGGTTTGGTAGTTTGCCATAACA

CTACTCCCAGGTTCGTTCCCTTTCCTCTTAGGTATGCTTGTGAATTTCTGGCTCAAGTATTTGCCATCCA

TGTGAACAAAGAAATAGAGTTAGAATATCAGATTATTGAGAAGAATATCCTGCGCACACAGACACTCTTG

TGTGATATGCTGATGCGAGATGCACCCCTTGGCATTGTATCACAGAGCCCTAATATAATGGATCTAGTGA

AATGTGATGGAGCAGCACTCTTATATAAGAACAAGTTATGGAGATTAGGAGTGACACCAAGTGAATCCCA

GGTAAGAGAGATAGCTTTGTGGCTGTCCGAGTACCATATGGATTCCACTGGCTTGAGTACGGATAGCTTG

TCTGATGCAGGCTTCCCATCGGCTCTTTCTCTGGGTGATGTTGTGTGTGGAATGGCAGCTGTCAGAATTA

CTTCGAAAGACGTGGTATTTTGGTTTCGGTCACACACTGCTGCAGAAATCCGATGGGGTGGTGCAAAGCA

TGAAGCTGGAGAAAAAGATGACGGTAGGAGGATGCATCCAAGATCGTCATTCAAGGCTTTCCTTCAAGTC

GTGAAGGCAAGGAGCTTGCCGTGGAAGGACTATGAAATGGATGCCATTCATTCCTTACAGTTAATACTGA

GAAATGCATTCAGAGATACTGAGGGTACAGATTTACAGACAAATGCAATTAATACAAAACTAAGTGATCT

GAAGATCGAAGGGATGCAGGAACTGGAAGCAGTTACAAGCGAGATGGTTAGGTTGATTGAAACAGCAACA

GTGCCTATTTTGGCAGTTGATGTCGATGGGCTGGTAAACGGGTGGAACATAAAAGTTGCTGAGTTGACAG

GCCTTCCAGTTGGTGAAGCTATCGGAAAGCATTTACTCACACTTGTCGAGGATTCTTCAACTGATAGAGT

CAAAAAGATGCTTGACTTGGCACTACAAGGTGAAGAAGAGAAGAATGTTCAATTTGAGATCAAAACGTTC

GGGTCCAAGATGGACTCTGGTCCTATTAGTCTAGTAGTAAACGCTTGTGCAAGCAGGGATCTTCGAGACA

ATGTTGTTGGTGTTTGCTTTGTGGCTCATGATATAACTGCTCAGAAGAATGTAATGGACAAATTCACCCG

TATTGAAGGTGATTACAAGGCAATTGTACAGAACCCCAATCCATTAATCCCCCCTATATTTGGCACAGAT

GAATTTGGCTGGTGTTGTGAGTGGAATCCAGCTATGTCAAAGTTAACTGGATGGAAGCGAGAGGAGGTAA

TGGATAAAATGCTTTTGGGAGAGGTTTTTGGCACCCAAATGTCTTGTTGTCGACTAAAGAATCAAGAAGC

TTTTGTTAATTTTGGCATTGTACTTAATAAAGCCATGACTGGCTCAGAAACAGAGAAAGTTGCTTTCGGT

TTCTTCGCTCGGAATGGCAAGTATGTAGAGTGCCTACTTTCTGTGAGTAAGAAGTTGGACGTAGAGGGCC

TAGTTACTGGGGTCTTCTGCTTCTTACAGCTAGCTAGCCCGGAACTCCAGCAAGCATTGCATATTCAGCG

TCTATCTGAGAAAACGGCCTTGAAGAGATTGAATGCATTAACTTACATGAAAAGGCAGATCAGGAATCCT

TTATGTGGGATTATATTTTCCCGGAAAATGTTGGAGGGTACTGAATTGGGAACAGAACAGAAACAATTTC

TGCATACTAGTGCTCAGTGCCAGCGGCAACTTAGCAAAATTCTTGATGACTCAGATCTTGACAGCATCTT

AGACGGTTACTTGGATCTTGAGATGGCTGAATTTACTCTGCATGAAGTAATGATTGCCTCCCTTAGCCAA

GTCATGACGAAAAGTAATGGAAAGAGTATCCGAATAGTCAATGATGTTGCAGAACAAATCGTAATGGAAA

CTTTATACGGTGATAGTCTAAGGCTTCAGCAGGTCTTGGCTGACTTCTTACTTATTTCCATCAATTTCAC

ACCAACTGGAGGCCAGGTTGCTGTAGCAGGCTCTCTAACCAAAGAACAGTTAGGGAAATCGGTCCATCTT

GTTAAGTTAGAGCTCAGCATAACACATGGTGGGAGTGGGGTGCCAGAAGCATTACTGAACCAGATGTTTG

GAAATAATGGACTAGAATCAGAGGAGGGTATGAGCCTACTGATCAGCAGAAAGCTGCTGAAGCTCATGAA

TGGAGATGTTCGCTATCTAAGGGAAGCAGGCAAATCAGCTTTTATCCTCTCTGCTGAACTTGCTGCAGCC

CATAATTTGAAAGCTTAAAGTTTTGGAAAAGAATAAGATGATGCAACACAATCTGTTTTGTACATCATAA

AGAGGACGGAGCTAGAAATTAATTGCATCCATTGTAACGCTTTATTTGTTTCACGTTTCTTTAACTACTT

CTTGTAACGTGTACGTTGTAGATAATGTATTATTAATCTTATTCTCATTTCTTGATCTA

4. Amino acid sequences retrieved from NCBI-BLAST for phylogenetic analysis

>XP_027330861.1 phytochrome A [Abrus precatorius]

MSSSRPSQSSSNSGRSRHSARIIAQTTVDAKLHATFEESGSSFDYSSSVRISGPATADGNHQPRSDKVTTAYLHHIQRGKLIQPFGCLLALDEKTCKVIAYSENAPEMLTMVSHAVPSVGDHPALGIGTDIRALFTAPSASALQKALGFAEVSLLNPILVHCKTSGKPFYAIIHRVTGSLIIDFEPVKPYEVPMTAAGALQSYKLAAKAITRLQSLPSGSMERLCDTMVQEVFELTGYDRVMAYKFHEDDHGEVIAEITKPGLEPYLGLHYPATDIPQAARFLFMKNKVRMIVDCHAKHVKVLQEEKLPFDLTLCGSTLRAPHSCHLQYMANMDSIASLVMAVVVNDNEEDGDSSDAVQPQKRKRLWGLVVCHNTTPRFVPFPLRYACEFLAQVFAIHVNKEIELEYQIIEKNILRTQTLLCDMLMRDAPLGIVSQSPNIMDLVKCDGASLLYKNKVWRLGVTPSESQIREIALWLSEYHMDSTGLSTDSLSDAGFPGALSLGDIVCGMAAVRITSKDIVFWFRSHTAAEIRWGGAKHEPGERDDGRKMHPRSSFKAFLEVVKARSLPWKDYEMDAIHSLQLILRNAFKDTEGTDLNTNAINTRLSDLKIEGMQELEAVTSEMVRLIETATVPILAVDVDGLVNGWNIKIAELTGLPVGEAIGKHLLTLVEDSSADRVKKMLDLALQGEEEKNVQFEIKTHGSKMDSGPISLVVNACASRDLRDNVVGVCFVAHDITAQKTVMDKFTRIEGDYKAIVQNPNPLIPPIFGTDEFGWCCEWNPAMTKLTGWKREEVMDKMLLGEVFGTQIACCRLKHQEAFVNFGIVLNKAMTGLETEKVAFGFFARNGKYVECLLSVSKKLDIEGVVTGVFCFLQLASPELQQALHIQRLSEQTALKRLKALTYMKRQIRNPLCGIVFSRKMLEATDLGTEQKQLLRTSAQCQRQLSKILDDSDLDSIIDGYLDLEMAEFTLHDILVASLSQVMTKGNGKGIRVVNDVAEQILMETLYGDSLRLQQVLADFLLISINFTPNGGQVVVAASLTKEQLGKSVHLVNLELSITHGGSGVPEALLNQMFGNNGLESEDGISLLISRKLLKLMNGDVRYLREAGKSSFILTAELAAAHKMKP

>XP_015968739.1 phytochrome A [Arachis duranensis]

MSSSRRSQSSSNSSRSRQSARVIAQTSVDAKLHANFEESGSSFDYSNSVRLSSGTVSGENQARCDRVTAAYLHQMQKGKFIQPFGCLLALDDKTLRVIAYSHNASEMLTMVSHAVPSVGDHTALAIGTDIRTIFTPSSAAALQKALAVPEVSLLNPILVHCKTSGKPFYAIVHRITASLIIDFEPVKPHEVPMTAAGALQSYKLAAKAITRLQSLPSGSMETLCDTMVQEVFELTGYDRVMAYKFHEDDHGEVIAEVAKPGLEPYLGLHYPATDIPQAARFLFMKNKVRMIVDCRAKHVKVLQDPKVSIDLTLCGSTLRAAHSCHLQYMENMNSIASLVMAVVVNDNDEDGDGSDVVQPQKRKRLWGLVVCHNTTPRFVPFPLRYACEFLAQVFAIHVNKELELEYQIVEKNILRTQTLLCDMLMRDAPLGIVSQSPNIMDLVRCDGAALLYRDKVWRLGVAPSESHIRELALWLSKCHKDSTGLSTDSLSDAGFPGAAALGDVVCGMAAVRISSMDIVFWFRSHTAAEIRWGGAKHEPGDRDDPTKMSPRSSFKAFLEVVKGRSLPWKDYEMDAIHSLQLILRNSFKDNEIMDISTQAIDTRLNDLKIEGMQELEAVTSEMVRLIETASVPILAVNVDGMVNGWNTKIAELTGLSVEEAIGKDLLTLVEDFSAERVKKMLDMALQGKEEKNFQFEIKTHGVKIDSGPISLVVNACASRNLQNSVVGVCFVAHDMTAEKTVMDKFTRIEGDYRAIVQNPNPLIPPIFGTDEFGWCCEWNSAMTKLTGWKREDVMDKMLLGEVFGTHMACCRLKNQEAVVNFGIVLNNAMTGVETEKAAFGFFTRKGKYVECLLSVSKKLDVEGEVTGVFCFLQTASPELQQALHIQRLSEQTALKRLKALTYMKRQIRNPLCGIVFSRKLLENTELGIEQKQLLDTGTQCQRQLSKILDDSDLDRIIDGYLDLEMVEFTLHQVFVACLSQVMTKSKAMGIHIINEVTEHIMTETLYGDSLRLQQVLADFLLVCINFTPTGGQVVVAASLTKDQLGKSVHLANLEISITHDGVGVPETLLNQMFGRDGQESEEGISLLISRKLLKLMNGDVRYLREAGKSSFILTVELAASQKLIA

>QHN85005.1 Phytochrome type A [Arachis hypogaea]

MSSSRRSQSSSNSSRSRQSARVIAQTSVDAKLHANFEESGSSFDYSNSVRLSSGTASGEKQARCDRVTAAYLHQMQKGKFIQPFGCLLALDDKTLRVIAYSHNASEMLTMVSHAVPSVGDHPALAIGTDIRTIFTPSSAAALQKALAVPEVSLLNPILVHCKTSGKPFYAIVHRITASLIIDFEPVKPHEVPMTAAGALQSYKLAAKAITRLQSLPSGSMETLCDTMVQEVFELTGYDRVMAYKFHEDDHGEVIAEVAKPGLEPYLGLHYPATDIPQAARFLFMKNKVRMIVDCRAKHVKVLQDPKVSIDLTLCGSTLRAAHSCHLQYMENMNSIASLVLAVVVNDNDEDGDGSDVVQPQKRKRLWGLVVCHNTTPRFVPFPLRYACEFLAQVFAIHVNKELELEYQIVEKNILRTQTLLCDMLMRDAPLGIVSQSPNIMDLVRCDGAALLYRDKVWRLGVAPSESHIRELALWLSKCHKDSTGLSTDSLSDAGFPGAAALGDVVCGMAAVRISSMDIVFWFRSHTAAEIRWGGAKHEPGDRDDPTKMSPRSSFKAFLEVVKGRSLPWKDYEMDAIHSLQLILRNSFKDNEIMDISTQAIDTRLNDLKIEGMQELEAVTSEMVRLIETASVPILAVNVDGMVNGWNTKIAELTGLSVEEAIGKDLLTLVEDFSVERVKKMLDMALQGKEEKNFQFEIKTHGVKIDSGPISLVVNACASRNLQNSVVGVCFVAHDMTAEKTVMDKFTRIEGDYRAIVQNPNPLIPPIFGTDEFGWCCEWNSAMTKLTGWKREDVMDKMLLGEVFGTHMACCRLKNQEAVVNFGIVLNNAMTGVETEKAAFGFFTRKGKYVECLLSVSKKLDVEGEVTGVFCFLQTASPELQQALHIQRLSEQTALKRLKALTYMKRKIRNPLCGIVFSRKLLENTELGIEQKQLLDTGTQCQRQLSKILDDSDLDRIIDGYDL

>XP_016174979.1 phytochrome A [Arachis ipaensis]

MSSSRPSQSSSNSGRSRHSARIIAQTTVDAKLHASFEESGSSFDYSSSVRASGSADGENQPRTDKVTTAYLHHIQKGKMIQPFGCLLALDEKTCKVIAYSENAPEMLTMASHAVPSVGDHPALGIGTDIRTIFTAPSASALQKALGFGEVHLLNPILVHCKTSGKPFYAILHRVTGSLIIDFEPVKPYEVPMTAAGALQSYKLAAKAITRLQSLPSGSMERLCDTMVQEVFELTGYDRVMAYKFHEDDHGEVIAELTKPGLEPYLGLHYPSTDIPQAARFLFLKNKVRMIVDCHAKHVKVVQDEKLPFDLTLCGSTLRAPHSCHLQYMSNMDSIASLVMAVIVNDSDEDADNSDAVQPQKRKRLWGLVVCHNTTPRFVPFPLRYACEFLTQVFAIHVNREIELEYQITEKNILRTQTLLCDMLMRDAPLGIVSQSPNIMDLVKCDGASLLYKNKVWRLGVTPTESHIREIALWLSEHHMDSTGLSTDSLYDAGFPGALSLGDVVCGMAAVRITEKDIVFWFRSHTAAEIRWGGAKHDPGEKDDGRRMHPRSSFKAFLEVVKSRSLPWKDYEMDAIHSLQLILRNAFKEMDSMDITTNAINTRLNDLRIEGMQELEAVTSEMVRLIETATVPILAVDVDGLVNGWNIKIAELTGLSVGDAIGKHLLTLIENSSVGIVKKMLEMALKGEEEKNVQFEIKTHGSKVDCGPIRLVVNACASRDIHDNVVGVCFVAQDITAQKTVMDKFTRIEGDYKAIVQNPNPLIPPIFGTDEFGWCCEWNAAMTKVTGWKREEVMDKMLLGEVFGTQMACCRLKNQEAFVNFGIVLNKAMTGLETAKVAFGFFARSGKYVECLLSVSKKLDVEGVVTGVFCFLQLASPELQQALHVQRISEQTALKRLKALTYMKRQIRNPLSGMMFSRKMLEATELGTEQKQLLHTSAQCQCQLSKVLDDSDLDSIIDGYLDLEMAEFTLHDVLVASLSQVMAKSNTKAIRIVNDVKEQIVTETLYGDSLRLQQAIADFLLISINFTPNGGQVVVTATLTKEQIGQSVHLVNLELSITHPGSGVPEALLNQMFESNGLESEEEGISLLISRKLLKLMNGDVRYVREAGKSSFILSAELAAA

HKLKD

>OAP14284.1 PHYA [Arabidopsis thaliana]

MSGSRPTQSSEGSRRSRHSARIIAQTTVDAKLHADFEESGSSFDYSTSVRVTGPVVENQPPRSDKVTTTYLHHIQKGKLIQPFGCLLALDEKTFKVIAYSENASELLTMASHAVPSVGEHPVLGIGTDIRSLFTAPSASALQKALGFGDVSLLNPILVHCRTSAKPFYAIIHRVTGSIIIDFEPVKPYEVPMTAAGALQSYKLAAKAITRLQSLPSGSMERLCDTMVQEVFELTGYDRVMAYKFHEDDHGEVVSEVTKPGLEPYLGLHYPATDIPQAARFLFMKNKVRMIVDCNAKHARVLQDEKLSFDLTLCGSTLRAPHSCHLQYMANMDSIASLVMAVVVNEEDGEGDAPDATTQPQKRKRLWGLVVCHNTTPRFVPFPLRYACEFLAQVFAIHVNKEVELDNQMVEKNILRTQTLLCDMLMRDAPLGIVSQSPNIMDLVKCDGAALLYKDKIWKLGTTPSEFHLQEIASWLCEYHMDSTGLSTDSLHDAGFPRALSLGDSVCGMAAVRISSKDMIFWFRSHTAGEVRWGGAKHDPDDRDDARRMHPRSSFKAFLEVVKTRSLPWKDYEMDAIHSLQLILRNAFKDSETTDVNTKVIYSKLNDLKIDGIQELEAVTSEMVRLIETATVPILAVDSDGLVNGWNTKIAELTGLSVDEAIGKHFLTLVEDSSVEIVKRMLENALEGTEEQNVQFEIKTHLSRADAGPISLVVNACASRDLHENVVGVCFVAHDLTGQKTVMDKFTRIEGDYKAIIQNPNPLIPPIFGTDEFGWCTEWNPAMSKLTGLKREEVIDKMLLGEVFGTQKSCCRLKNQEAFVNLGIVLNNAVTSQDPEKVSFAFFTRGGKYVECLLCVSKKLDREGVVTGVFCFLQLASHELQQALHVQRLAERTAVKRLKALAYIKRQIRNPLSGIMFTRKMIEGTELGPEQRRILQTSALCQKQLSKILDDSDLESIIEGCLDLEMKEFTLNEVLTASTSQVMMKSNGKSVRITNETGEEVMSDTLYGDSIRLQQVLADFMLMAVNFTPSGGQLTVSASLRKDQLGRSVHLANLEIRLTHTGAGIPEFLLNQMFGTEEDVSEEGLSLMVSRKLVKLMNGDVQYLRQAGKSSFIITAELAAA

NK

>XP_020226040.1 phytochrome A [Cajanus cajan]

MSTSRPSQSSSNSGRSRHSARVLAQTTVDAKLHATFEESGSSFDYSSSVRVSGTADGVNQPRSDKVTTAYLHHIQRGKMIQPFGCLLALDEKTCKVIAYSENAPEMLTMVSHAVPSVGDHPALGIGTDIKTLFTAPSASALQKALGFAEVSLLNPILVHCKTSGKPFYAIIHRVTGSLIIDFEPVKPYEVPMTAAGALQSYKLAAKAITRLQSLPSGSIERLCDTMVQEVFELTGYDRVMAYKFHEDDHGEVIAEITKPGLEPYLGLHYPATDIPQASRFLFTKNKVRMIVDCHAKHVKVLQDEKLPFDLTLCGSTLRAPHSCHAQYMSNMDSIASLVMAVVVNDNEEDGDTDAVQPQKRKRLWGLVVCHNTTPRFVPFPLRYACEFLAQVFAIHVNKEIELEYQIIEKNILRTQTLLCDMLMRDAPLGIVSQSPNIMDLVKCDGAALLYKNKVWRLGVTPSESQIRDIALWLSEYHMDSTGLSTDSLSDAGFPSALSLGDIVCGMAAVRITAKDVVFWFRSHTAAEIRWGGAKHEPGEKDDGRRMHPRSSFKAFLQVVKSRSLPWKDYEMDAIHSLQLILRNAFKDTESTDIQTNAINTRLSDLKIEGMQELEAVTSEMVRLIETATVPILAVDVDGLVNGWNIKIAELTGLPVSEAIGKHLLTLVEDSSTDRVKKMLDLALQGEEEKNVQFEIKTHESKMDSGPISLVVNACASRDLRENVVGVCFVAHDITAQKNVMDKFTRIEGDYKAIVQNRNPLIPPIFGTDEFGWCCEWNPAMTKLTGWKREEVMDKMLLGEVFGTQMACCRLKNQEAFVNFGIVINKAMTGSETEKVAFGFFARNGKYVECLLSVSKKLDVEGLVTGVFCFLQLASPELQQALHIQRLSEQTALKRLNALTYMKRQIRNPLCGIIFSRKMLEGTDLGTEQKQLLHTSAQCQRQLSKILDDSDLDSIIDGYLDLEMAEFTLHEVLVASFSQVMTKSNGKSIRIVNDVTEQIVMETLYGDSLRLQQVLADFLLISINFTPNGGQVVVAGSLTKEQLGKSVHLVKLELSITHGGSGVPEALLNQMFGNNGLESEEGISLLISRKLLKLMNGDVRYLREAGKSAFILSAELAAAHN

LKA

>XP_004495828.1 phytochrome A [Cicer arietinum]

MSTSRPSQSSTNSGRSRHSARIIAQTTVDAKLHATFEESSSSFDYSSSVRVSGSVDGDHQPRSNKVTTAYLNHIQRGKQIQPFGCLLALDEKTCKVVAYSENAPEMLTMVSHAVPSVGDHPALGIGTDIRTIFTAPSASALQKALGFAEVSLLNPILVHCKTSGKPFYAIIHRVTGSLIIDFEPVKPYEVPMNAAGALQSYKLAAKAITRLQSLPSGSMERLCDTMVQEVFELTGYDRVMAYKFHEDDHGEVIAEIAKPGLEPYLGLHYPATDIPQAARFLFMKNKVRMIVDCHAKHVKVLQDEKLPFDLTLCGSTLRAPHSCHLQYMANMDSIASLVMAVVVNDSDEDSDSTDAVHPQKKKRLWGLVVCHNTTPRFVPFPLRYACEFLAQVFAIHVNKEIELEYQILEKNILRTQTLLCDMLMRDAPLGIVSQSPNIMDLVKCDGAALLYKNNLWILGVTPSESKIREIALWMSEYHTDSTGLSTDSLSDAGFPGALSVGDTVCGMAAVRITPKDIVFWFRSHTAAEIRWGGAKHEPSEQDDGRKMHPRSSFKAFLEVVKARSLPWKDFEMDAIHSLQLILRNASKDTESVDLNTKAINTRLNDLKIEGMQELEAVTSEMVRLIETATVPILAVDVDGMVNGWNIKIAELTGLPVDEAIGKHLLTLVEDSSSDIVKKMLNLALQGEEEKNVQFEIKTHASKMDSGPISLIVNACASKDLRDNVVGVCFVAQDITAQKTVMDKFTRIEGDYKAIVQNPNPLIPPIFGTDEFGWCCEWNQAMIKLTGWKREEVMDKMLLGEVFGTQMACCRLKNQEAFVNFGIVLNKAMTGFETQKVAFGFFARNGKYVECLLSVSKKLDAEGLVTGVFCFLQLASPELQQALHIQRLSEQTALKRLKVLHYMKRQIRNPLSGIVFSSKMLEGTDLGTEQKRLLSASAQCQRQLSKILDDSDLDSIIDGYLDLEMAEFTLHEVLVTALSQVVTRSNTKGIRIVNDVAEHIAMETLYGDSLRLQQVLADFLLISINSSPNGGQVVIAASLTKEQLGKSVHLVNLELSITHGGSGVAEALLNEMFGNNVLESEEGISLHISRKLLKLMNGDVRYLKEAGKSSFILSVELAAAH

KLKG

>AHA82658.1 phytochrome A [Glycine max]

MSSSRPSQSSSNNSGRSRTSRLSARRMAQTTLDAKLHATFEESGSSFDYSSSVRMSPAGTVSGDHQPRSDRATSSYLHQTQKIKLIQPFGCLLALDEKTCKVIAYSENAPEMLTMVSHAVPSVGDHPALGIGTDIRTIFTAPSSAAIQKALRFGDVSLHNPILVHCKTSGKPFYAIIHRVTGSVIIDFEPVKPHEVPMTASGALQSYKLAAKAITRLESLTTGNMETLCNTMVREVFELTGYDRVMAYKFHEDDHGEVIAEVKRPGLEPYLGLHYPATDIPQATRFLFMKNKVRMIVDCCAKHVNVLQDKKIPFDLTLCGSTLRAAHSCHLQYMENMNSSASLVMAVVVNDNDEDGDSSDAVQPQKSKRLWGLVVCHHTTPRFVPFPLRYACQFLAQVFAVHVSKELEIEYQIIEKNILQTQTLLCDMLVQGEPLGIVSQSPNIMDLVKCDGAALLYKNKVWRLGVTPSESQIKEIALWLFECHEDSTGFCTDSLSDAGFPGAAALGDIACGMAAARIASKDILFWFRSHTASEIRWGGAKHEPGERDDGRRVHPRSSFKAFLEVVKTRSLPWKTYETDAIHSLQLILRDAFKETQSMEISTYAIDTRLGDLKIEGMQELDAVTSEVVRLIETATVPILAVDVNGMINGWNTKIAELTGLPVDEAIGKHLLTLVEDFSVDRVKKMLDMALQGEEERNVQFEIQTHHMKIDSGPISLVVNACASRDLQDNVVGVCFLAQDITAQKTMMDKFTRIEGDYKAIVQNPNPLIPPIFGTDEFGWCCEWNSAMAKLTGWKREEVMDKMLLGEVFGTQIACCRLRNHEAVVNFSIVLNAAMAGLETEKVPFGFFARDGKHVECILSMTKKLDAEGVVTGVFCFLQLASAELQQALHIQRISEQTSLKRLKDLTYLKRQIQNPLYGIMFSRKLLEGTELGAEQKQFLQTGIRCQRQISKILDDSDLDSIIDGYMDLEMVEFTLHEVLVASLSQVMTKSNAKGIRVVNDVEEKITTETLYGDSIRLQQVLADFLLISINFTPTGGQVVVAATLTQQQLGKLVHLANLEFSITHDSFGVPETLLNQMFGRDGHESEEGISMLISRKLLKLMNGDVRYLREAGKSSFILSVELAAAHKSNT

>XP_028222188.1 phytochrome A-like [Glycine soja]

MSTSRPSQSSSNSGRSRRSARAMALATVDAKLHATFEESGSSFDYSSSVRISGTADGVNQPRHDKVTTAYLHHMQKGKMIQPFGCLLALDEKTCKVIAYSENAPEMLTMVSHAVPSVGDHPALGIGTDIKTLFTAPSASALQKALGFAEVSLLNPVLIHCKTSGKPFYAIIHRVTGSMIIDFEPVKPYEVPMTAAGALQSYKLAAKAITRLQSLPSGSMERLCDTMVQEVFELTGYDRVMAYKFHEDDHGEVIAEITKPGLEPYLGLHYPATDIPQASRFLFMKNKVRMIVDCHAKHVRVLQDEKLPFDLTLCGSTLRAPHSCHAQYMANMDSIASLVMAVVVNDNEEDGDTDAIQPQKRKRLWGLVVCHNTTPRFVPFPLRYACEFLAQVFAIHVNKEIELEYQIIEKNILRTQTLLCDLVMRDAPLGIVSESPNIMDLVKCDGAALIYKNKVWRLGVTPSESQIREIAFWLSEYHMDSTGFSTDSLSDAGFPSALSLGDVVCGMAAVRVTAKDVVFWFRSHTAAEIRWGGAKHEAGEKDDGRRMHPRSSFKVFLDVVKARSLPWKEYEIDAMHSLQLILRNAFKDTESMDLNTKAINTRLSDLKIEGMQELEAVTSEIVRLIETATVPILAVDVDGLVNGWNIKIAELTGLPVGEAMGKHLLTLVEDSSTDRVKKMLNLALLGEEEKNVQFEIKTHGSKMDSGPISLVVNACASRDLRDNVVGVCFVAHDITAQKNVMDKFTRIEGDYKAIVQNRNPLIPPIFGTDEFGWCCEWNPAMTKLTGWKREEVMDKMLLGELFGTHMAACRLKNQEAFVNLGVVLNKAMTGLETEKVPFGFFARNGKYVECLLSVSKKLDVEGLVTGVFCFLQLASPELQQALHIQRLSEQTALKRLNALSYMKRQIRNPLCGIIFSRKMLEGTALGTEQKQLLRTSAQCQQQLSKILDDSDLDSIIDGYLDLEMAEFTLHEVLVTSLSQVMTKSNGKSIRIVNDVAEQIVMETLYGDSLRLQQVLADFLLISINFTPNGGQVVVAGTLTKEQLGKSVHLVKLELSITHGGSGVPEALLNQMFGNNGLESEEGISLLISRKLLKLMNGDVRYLREAGKSAFILSAELAAAHNLKG

>BAN14693.1 phytochrome A [Lotus japonicus]

MSSSRPSQSSNNSGRSRHSARVIAQTTVDAKIHANFEESGSSFDYSSSVRASGTADADHQPKSNKVTTAYLHHIQRGKLIQPFGCLLALDEKTCKVIAYSENAPEMLTMVSHAVPSVGEHPALGIDTDIRTIFTAPSASALQKALGFAEVTLLNPILVHCKTSGKPFYAIIHRVTGSLIIDFEPVKPYEVPMTAAGALQSYKLAAKAITRLQSLPSGSMERLCDTMVQEVFELTGYDRVMAYKFHEDDHGEVIAEITKPGLEPYLGLHYPATDIPQASRFLFMKNKVRMIVDCHAKQVKVLIDEKLPFDLTLCGSTLRAPHSCHLQYMANMDSIASLVMAVVVNDNDEDGDGSDSVQPQKRKRLWGLVVCHNTSPRFVPFPLRYACEFLAQVFAIHVNKEIELECQILEKNILRTQTLLCDMLMRDAPLGILTQSPNLMDLVKCDGAALLYKNKVWMLGVTPSELHIRDIASWLSKYHTDSTGLSTDSLSDAGFPGALSLGDLVCGMAAVRITPKDVVFWFRSHTAAEIRWGGAKHEPGEQDDGKKMHPRSSFKAFLEVVRARSSPWKDYEMDAIHSLQLILRNAFKDTDSMDINTTAIDTRLSDLKIEGMQELEAVTSEMVRLIETATVPILAVDIDGLVNGWNIKIAELTGLPVGEAIGKHLLTLVEDCSTDRVKKMLDLALSGEEEKNVQFEIKTHGSKMESGPISLVVNACASRDLRENVVGVCFVAQDITAQKTVMDKFTRIEGDYKAIVQNPNPLIPPIFGTDEFGWCCEWNPAMTKLTGWKREEVMDKMLLGEVFGTHMAACRLKNQEAFVNFGIVLNKAMTGSETEKVGFGFFARSGKYVECLLSVSKKLDVEGLVTGVFCFLQLASPELQQALHIQRLSEQTALKRLKALTYMKRQIRNPLSGIVFSRKTLEGTDLGIEQKRLVHTSAQCQRQLSKILDDSDLDSIMDGYLDLEMAEFTLQDVLITSLSQIMARSSARGIRIVNDVAEEIMVEILYGDSLRLQQVLADFLLISINCTPNGGQVVVAASLTKEQLGKSVHLANLELSITHGGSGVPEALLNQMFGNDGLESEEGISLLISRKLLKLMSGDVRYLREAGKSSFILSVELAAAHKLKA

>KAE9618885.1 Phytochrome A [Lupinus albus]

MSSSRPSQSSTNSGRPRNSARVIAQTTVDAKLHANFEESGCSFDYSSSVRVSGTVGGDHQPRSDRVTTAYLHHIQKGKQIQPFGCLLALDEKTCKVIAYSENAPEMLTMMSHAVPNVVDHPALGIGTDIRTIFTAPSASALQKALGFGEVSLLNPILVHCKTSGKPFYAIIHRVTGSLIIDFEPVKPYEVPMTAAGALQSYKLAAKAITRLQSLPSGSMERLCDTMVQEVFELTGYDRVMAYKFHEDDHGEVIAEIAKPSLESYLGLHYPATDIPQASRFLFMKNKVRMIVDCHARHVKVLQDEKLPIDLTLCGSTLRAPHSCHLQYMANMDSIASLVMAVVVNDNDEDGDTSDAVQPQKRKRLWGLVVCHNTTPRFVPFPLRYACEFLTQVFAIHVNKEIELELQIIEKNILRTQTLLCDMLMRDAPLGIVSQSPNIMDLVKCDGAALFYKNKVWRLGVTPSESQIREIALWLSEHHMDSTGLSTDSLLDAGFPGALSLGDTVCGMAAVRIASKDTVFWFRSHTASEIRWGGAKQEPGERDDGRKMHPRSSFKAFLEVVKTRSLPWKDYEMDAIHSLQLILRNAFRDTVTTHINTSAIDTRLSDLRIEGMQELEAVASEMVRLIETATVPILAVDINGLVNGWNTKIAELTGLPVGDAIGKHLLTLVEDSSIDRVKKMLDMALQGEEEKNVQFEIKTHGSYMDSGPVSLVVNACASRDLRDNVVGVCFVAHDITAQKTVMDKFTKIEGDYKAIVQNPNPLIPPIFGTDEFGWCCEWNQAMTKLTGWKREEVMDKMLLGEVFGTQKACCRLKNQEAFVNFGIVLNEAMTGSETAKVAFGFIARNGKYVDCLLSVSKKFDTDGAVTGIFCFLQLASPELQRALHIQRLSEKTALKRLKSLSYIKRQIRNPLSGIMFTRKLLEGTELGTEQVQLLHTSAQCQRQLSKIIEDSDLDSIIDGCLDLEMAEFTLHDVLAASLSQVMLKCNAKGIQIINDVAEQIRIETLYGDCLRLQQVVADFLVISINFTPNGGQVVVAASLTKQQLGQSVHLVNLELSITHAGSGVPESLLNQMFENGGQESEEGISLLISRKLLKLMSGDVRYLREADKSSLILSVELAAAHKLKP

>XP_019452320.1 PREDICTED: phytochrome A-like [Lupinus angustifolius]

MSSSRPSQSSNNSGRSRNSARIIAQTTVDAKLQANFEESGCSFDYSSSVRVSDTVNGDHQPRSDKVTTAYLHHIQKGKQIQPFGCLLALDEKTCKVIAYSENAPEMLTMMSHAVPSVGDHPALGIGTDIRTIFTAPSASALQKALGFGEVSLLNPILVHCKTSGKPFYAIIHRVTASLIIDFEPVKPYEVPMTAAGALQSYKLAAKAITRLQSLPSGKMEKLCDTMVQEVFELTGYDRVMAYKFHEDDHGEVIAEIAKPGLEPYLGLHYPATDIPQASRFLFMKNKVRMIVDCHARHVRVLQDEKLPIDLTLCGSTLRAPHSCHLQYMANMNSIASLVMAVVVNDNDEDGDSSDAVQPQKRKRLWGLVVCHNTTPRFVPFPLRYACEFLAQVFAIHVNKEIELELQIIEKNILRTQTLLCDMLMRDAPLGIVSQSPNIMDLVKCDGAALFYKNKVWRLGVTPSESEIRDIALWLSEHHMDSTGLSTDSLLDAGFPGALSLGDIVCGMAAVRISSKDTVFWFRSHTAAEIRWGGAKHEPGESDDSRKMHPRSSFKAFLEVVKTRSLPWKDYEMDAIHSLQLILRNAFRDTVTTHINTSAIDTRLSDLKIEGMQELEAVASEMVRLIETATVPILSVDVNGLVNGWNTKIAELTGLPVGEAIGKHLLTLVEDSSIDRVKKMLDMALQGEEEKNVQFEIKTHRSYIDSGPVSLVVNACASRDLRDNVVGVCFVAHDITVQKTVMDKFTKIEGDYKAIMQNPNPLIPPIFGTDEFGWCCEWNQAMTKLTGWKRDQVMDKMLLGEVFGTQAACCRLKNQEAFVNLGIVLNKAMTGSETSKEAFGFIARNGKCVDCLLSVSKKLGTDGAVTGIFCFLQLASPELQRALHIQRLSEKTALKRLKSLSYIKRQIGNPLSGIMFSRKLLEGTELGTEQIQLLHTSAQCQRQLSKIVEDSDLDSIIDGYLDLEMTEFTLHDVLAASLSQVMLKCNSKGIQIINSVAEQIRMETLYGDCLRLQQVFADFLLISINFTPNGGQVVVAASLTKEQLGQSIHLVNLELSITHTGIGVPEALLNQMFGNDEHESEEGISLLISRKLLKLMNGDVRYLQEAGKSSLILSVELAAAHKMKT

>KAE9618885.1 Phytochrome A [Lupinus albus]

MSSSRPSQSSTNSGRPRNSARVIAQTTVDAKLHANFEESGCSFDYSSSVRVSGTVGGDHQPRSDRVTTAYLHHIQKGKQIQPFGCLLALDEKTCKVIAYSENAPEMLTMMSHAVPNVVDHPALGIGTDIRTIFTAPSASALQKALGFGEVSLLNPILVHCKTSGKPFYAIIHRVTGSLIIDFEPVKPYEVPMTAAGALQSYKLAAKAITRLQSLPSGSMERLCDTMVQEVFELTGYDRVMAYKFHEDDHGEVIAEIAKPSLESYLGLHYPATDIPQASRFLFMKNKVRMIVDCHARHVKVLQDEKLPIDLTLCGSTLRAPHSCHLQYMANMDSIASLVMAVVVNDNDEDGDTSDAVQPQKRKRLWGLVVCHNTTPRFVPFPLRYACEFLTQVFAIHVNKEIELELQIIEKNILRTQTLLCDMLMRDAPLGIVSQSPNIMDLVKCDGAALFYKNKVWRLGVTPSESQIREIALWLSEHHMDSTGLSTDSLLDAGFPGALSLGDTVCGMAAVRIASKDTVFWFRSHTASEIRWGGAKQEPGERDDGRKMHPRSSFKAFLEVVKTRSLPWKDYEMDAIHSLQLILRNAFRDTVTTHINTSAIDTRLSDLRIEGMQELEAVASEMVRLIETATVPILAVDINGLVNGWNTKIAELTGLPVGDAIGKHLLTLVEDSSIDRVKKMLDMALQGEEEKNVQFEIKTHGSYMDSGPVSLVVNACASRDLRDNVVGVCFVAHDITAQKTVMDKFTKIEGDYKAIVQNPNPLIPPIFGTDEFGWCCEWNQAMTKLTGWKREEVMDKMLLGEVFGTQKACCRLKNQEAFVNFGIVLNEAMTGSETAKVAFGFIARNGKYVDCLLSVSKKFDTDGAVTGIFCFLQLASPELQRALHIQRLSEKTALKRLKSLSYIKRQIRNPLSGIMFTRKLLEGTELGTEQVQLLHTSAQCQRQLSKIIEDSDLDSIIDGCLDLEMAEFTLHDVLAASLSQVMLKCNAKGIQIINDVAEQIRIETLYGDCLRLQQVVADFLVISINFTPNGGQVVVAASLTKQQLGQSVHLVNLELSITHAGSGVPESLLNQMFENGGQESEEGISLLISRKLLKLMSGDVRYLREADKSSLILSVELAAAHKLKP

>XP_013469103.1 phytochrome A [Medicago truncatula]

MSTTRPSQSSNNSGRSRNSARIIAQTTVDAKLHATFEESGSSFDYSSSVRVSGSVDGEHQPRSNKVTTAYLNTIQRGKQIQPFGCLLALDEKTCKVIAYSENAPEMLTMVSHAVPSVGDHPALGIGTDIRTIFTAPSASALQKALGFAEVSLLNPILVHCKTSGKPFYAIIHRVTGSLIIDFEPVKPYEVPMTAAGALQSYKLAAKAITRLQSLPSGSMERLCDTMVQEVFELTGYDRVMAYKFHEDDHGEVIAEVTKTGLEPYLGLHYPATDIPQAARFLFMKNKVRMIVDCHAKHVKVLQDEKLPFDLTLCGSTLRAPHSCHLQYMANMDSIASLVMAVVVNDSDEDGDSADAVLPQKKKRLWGLVVCHNTTPRFVPFPLRYACEFLAQVFAIHVNKEIELEFQILEKNILRTQTLLCDMLMRDAPLGIVSQSPNIMDLVKCDGAALLYRNKLWILGATPSEPQIREIALWMSEYHTDSTGLSTDSLSDAGFPGALKLNDTVCGMAAVRITSKDIVFWFRSHTAAEIRWGGAKHEPGEQDDGRKMHPRSSFKAFLEVVKARSIPWKDFEMDAIHSLQLILRNASKDTDMIDLNSKAINTRLNDLKIEGMQELEAVTSEMVRLIETATVPILAVDVDGMVNGWNIKISELTGLPVGEAIGKHLLTLVEDSSTDIVKKMLNLALQGQEEKNVQFEIKTHGSKTDCGPISLIVNACASRDLHENVVGVCFVAQDITAQKTVMDKFTRIEGDYKAIVQNPNQLIPPIFGTDEFGWCCEWNAAMIKITGWKREEVMDKMLLGEVFGTHMSCCRLKNQEAFVNFGIVLNKAMTGLETEKVPFGFLSRKGKYVECLLSVSKKIDAEGLVTGVFCFLQLASPELQQALHIQRLSEQTALKRLKVLTYMRRQIRNPLSGIVFSSKMLENTELGTEQKRIVNTSSQCQRQLSKILDDSDLDSIIDGYLDLEMAEFTLHEVLVTSLSQVMNRSNTRSIRIVNDVAEHIAMETLYGDSLRLQQVLADFLLISINSTPNGGQVVIAATLTKEQLGKSVHLVNLELSITHGGNGVAEAVLNQMFGNNGLESEEGISLHISRKLLKLMNGDVRYLKEAGKSSFILSVELAAAQKLRG

>XP_007163278.1 hypothetical protein PHAVU_001G221100g [Phaseolus vulgaris]

MSSSRPSQTSSNNSGRSRSSRRSARVLAQTTLDAKLHATFEESGSSFNYSNSVRLSSGTGTVSGDHQTRSDGVTTAYLHQLQKSKLIQPFGCLLALDEKTCKVIAYSENAPEMLTMVSHAVPSVGEHPGLGIGTDIRTIFTAPSSTSIQKALGFGEVSLLNPILVHCKSSGKPFYAIIHRVTGSVIIDFEPVKPHEVPMTAAGALQSYKFAAKAITKLQSLPSGSMDTLCDTMVREVFELTGYDRVMAYKFHEDDHGEVIAEVKRPGLEPYLGLHYPATDFPQATRFLFLKNKVRMIVDCRAKHVKVLQDKNIPFELTLCGSTLRAAHSCHLQYMQNMNSSASLVMAVVVNDNDEDGDSSDAVQPQKRKRLWGLVVCHHSTPRFVPFPLRYACEFLAQVFAIHVNKELELEYQIVEKNILRTQTLLCDMLMRDAPLGIVSQSPNIMDLVNCDGAALLYKNKIWRLGVTPSESQIREIALWLSECHRDSTGLSTDSLSDAGFPGAATLGDITCGMAAVRIASKDIVFWFRSHTAAEIRWGGAKHEPSERDDGRRMHPRSSFKAFLEVMKTRSLPWKDYEMDVIHSLQLILRNAFKDTERMEISTYAINTRLGDLKIEGMQDLEAVTSEMVRLIETATVPILAVDVNGMVNGWNKKIAELTCLPVEQAIGKHLLTLVEDFSVDRVKKMLDMALQGEEEKNVQFEIKTHDLKIDSGPISLVVNACASRDLQDNVVGVCFVAQDITAQKTVMDKFTRIEGDYKAIVQNRNPLIPPIFGTDEFGWCCEWNSAMTKLTGWKREEVMDKMLLGEVFGTQIAYCRLRNQEAVVNFSIVVNKAMAGLETEKVPFSFFTRDGKHVECLLSVSKKLDAESVVTGVFCFLQLASPELQQALHIQRLSEQTASKRLKALTYLKRQIQSPLYGIVFSRKLLEGAELGAEQKQFLQTGGRCERQLSKVLDSDLDSIVDGYLDLEMVEFTLHEVLVASLSQAMTKGNAKGIRLVSDVEELITRETLYGDGLRLQQVLADFLLISINFTPNGGQIVVAASLSKQQLGKLVHLANLELSITHEGFGIPETLLNQMFGRDGDESEEGISMLISRKLLNLMNGEVRYIREAGKSSFILSVELAAAHKSNS

>sp|P15001.1|PHYA_PEA RecName: Full=Phytochrome A

MSTTRPSQSSNNSGRSRNSARIIAQTTVDAKLHATFEESGSSFDYSSSVRVSGSVDGDQQPRSNKVTTAYLNHIQRGKQIQPFGCLLALDEKTCKVVAYSENAPEMLTMVSHAVPSVGDHPALGIGTDIRTVFTAPSASALQKALGFAEVSLLNPILVHCKTSGKPFYAIIHRVTGSLIIDFEPVKPYEVPMTAAGALQSYKLAAKAITRLQSLASGSMERLCDTMVQEVFELTGYDRVMAYKFHEDDHGEVIAEIAKPGLEPYLGLHYPATDIPQAARFLFMKNKVRMIVDCNAKHVKVLQDEKLPFDLTLCGSTLRAPHSCHLQYMANMDSIASLVMAVVVNDSDEDGDSADAVLPQKKKRLWGLVVCHNTTPRFVPFPLRYACEFLAQVFAIHVNKEIELEYQILEKNILRTQTLLCDMLMRDAPLGIVSQSPNIMDLVKCDGAALFYRNKLWLLGATPTESQLREIALWMSEYHTDSTGLSTDSLSDAGFPGALSLSDTVCGMAAVRITSKDIVFWFRSHTAAEIRWGGAKHEPGDQDDGRKMHPRSSFKAFLEVVKARSVPWKDFEMDAIHSLQLILRNASKDTDIIDLNTKAINTRLNDLKIEGMQELEAVTSEMVRLIETATVPILAVDVDGTVNGWNIKIAELTGLPVGEAIGKHLLTLVEDSSTDIVKKMLNLALQGEEEKNVQFEIKTHGDQVESGPISLIVNACASKDLRENVVGVCFVAQDITAQKTVMDKFTRIEGDYKAIVQNPNQLIPPIFGTDEFGWCCEWNAAMIKLTGWKREEVMDKMLLGEVFGTQMSCCRLKNQEAFVNFGIVLNKAMTGLETEKVPFGFFSRKGKYVECLLSVSKKIDAEGLVTGVFCFLQLASPELQQALHIQRLSEQTALKRLKVLTYMKRQIRNPLAGIVFSSKMLEGTDLETEQKRIVNTSSQCQRQLSKILDDSDLDGIIDGYLDLEMAEFTLHEVLVTSLSQVMNRSNTKGIRIANDVAEHIARETLYGDSLRLQQVLADFLLISINSTPNGGQVVIAASLTKEQLGKSVHLVNLELSITHGGSGVPEAALNQMFGNNVLESEEGISLHISRKLLKLMNGDVRYLKEAGKSSFILSVELAAAHKLKG

>XP_028779403.1 phytochrome A [Prosopis alba]

MSSSRPSQSSSNSGRSRHTARVIAQTTVDAKLHANFEESGSSFDYSNSVRISGTVNGEQQPRSDRVTTAYLHHMQKGKLIQPFGCLLALDEKTCKVIAYSENAPEMLTMVSHAVPSVGDNAALGIGTDIRTIFTAPSASALQKALGFGEVSLLNPILVHCKTSGKPFYAIIHRVTGSLIIDFEPVKPFEVPMTAAGALQSYKLAAKAITRLQSLPSGSMERLCDTMVQEVFELTGYDRVMAYKFHEDDHGEVIAEITKPGLEPYLGLHYPATDIPQASRFLFMKNKVRMIVDCRARHVKVLQDEKLPFELTLCGSTLRAPHSCHLQYMENMNSIASLVMAVVVNDSDEDVDSSDTVQPQKRKRLWGLVVCHNTTPRFVPFPLRYACEFLAQVFAIHVNKELELENQIVEKNILRTQTLLCDMLMRDAPLGIISQSPNIMDLVKCDGAALLYKNKVWRLGVTPSESQIREISIWLSENHKDSTGLSTDSLAEAGLPSALALGDIACGMAAVRISSKDMVFWFRSHTAAEIRWGGAKHEPGEQDDNRRMHPRSSFKAFLEVVKSRSLPWKDYEMDAIHSLQLILRNAFKDTESVVDVNTNAINTGLSDLRIEGMQELEAVTSEMVRLIETATVPILAVDVDGLVNGWNTKIAELTGLQVDEAIGKHLLTLVEDSSTDRVKRMLDMALQGEEEQNVQFEIKTHAAEIDSGPIRLVVNACASRDLRDNVVGVCFVAQDITAQKTVMDKFTRIEGDYKAIVQNPNPLIPPIFGTDEFGWCCEWNPAMIKLTGWKREEVMDKMLLGEVFGTHAACCRLKNQEAFVNFGVVLNKAMAGLETEKVAFGFFSRNGKYNECLLSVSKKLDIEGTVTGVFCFLQLASPELQQALHIQRLSEQTAMKRLKALTYMKRQIRNPLCGIVFSRKMLEGTELGTEQKQLLHTSSLCQRQLSKVLDDSDLDSIIDGCLDLEMVEFTLHEVLVASLSQVMTKSNAKGITIVNEVAEQIMTETLYGDCLRLQQVLADFLLMSVTFTPSEGKVVVAASLTKDQLGQSVHLANLELSITHAGSGIPEALLNEMFGSDGQESEEGINLLISRKLVKLMNGDVRYLREAGKSSFILTVELAAA

QK

>TKY61615.1 Phytochrome type A [Spatholobus suberectus]

MSSSRPSQSSSNNSGRSSRHSARILAQTTLDAKLHATFEESGSSFDYSNSVRLSPGTGTTSGTGNHQSRSDSVTSAYLHQIQKSKLIQPFGCLLALDEKTHKVIAYSENAPEMLTMVSHAVPSVGDHLALGIGTDIRTIFTAPSAAAFQKALGFGEVSLLNPILVHCKTSGKPFYAIIHRVTSSVIIDFEPVKPHEVPMTAAGALQSYKLAAKAITRLQSLPSGSLETLCDTMVQEVFELTGYDRVMAYKFHEDDHGEVIAEVKRPGLEPYLGLHYPATDIPQVTRFLFMKNKVRMIVDCRAKHVKVLQDRKIPFDLTLCGSTLRAAHSCHLQYMENMNSSASLVMAVVVNDNDEDGDSSDAVQPQKRKRLWGLVVCHHTTPRFIPFPLRYACEFLAQVFAIHVNKELELEYQIVEKNILRTQTLLCDMLMRDAPLGIVSQSPNIVDLVKCDGAALLYKNKVWRLGVTPSESQIREIALWLSECHRDSTGLSTDSLSDAGFPGSAALGDIACGMAAVRIASKDIVFWFRSHTAAEIRWGGAKHEPGERDDGRRMHPRSSFKAFLEVVKTRSLPWKDYEMDAIHSLQLILRNAFKDTENMEISTYAINTRLGDLKIEGMQELEAVTSEMVRLIETATVPILAVDVQGMVNGWNTKIAELTGLPVEEAIGKHLLMLVEDFSVDRVQKMLDMALQGEEEKNVQFEIKTHDLKIDSGPISLVVNACASRDLQDNIVGVCFVAQDITAQKTVMDKFTRIEGDYKAIVQNPNPLIPPIFGTDEFGWCCEWNSAMTKLTGWKREEVMDKMLLGEVFGTQIACCRLRNQEAVVNFSIVLNKGMTGMETEKVPFGFFARDGKHVECLLSVSKKLDAEGVVTGVFCFLQLASPELQQALHIQHLSEQTALKRLKALTYLKRQIQNPLCGIIFSRKLLESTELGAEQKQFLQTGNRCQHQLSKILDDSDLDSIIDGYLDLEMVEFTLHEILVASLSQVMTKSNAKAIRVVNDVEEQIITETLYGDSLRLQQVLADFLLISINFTPTGGQVAVAASLTKHQLGKLVHLANLEISITHDGLGVPEGLLNQMFGRDGHESEEGISMLISRKLLKLMNGEVRYIREAGKSSFILSVELASAHKSMA

>KAG2406446.1 Phytochrome protein [Vigna angularis]

MSSSRPSQSSSNNSGRSRSSRRSARVLAQTTLDAKLRATFEESGSSFNYSNSVRLSPGTGTASGDHQPRSDEVTTAYLHQLQKSKLIQPFGCLLALDEKTFRVIAYSENAPEMLTMVSHAVPSVGDHPALGIGTDIRTIFTSPSSTSIQKALGFGEVSLLNPILVHCKSSGKPFYAIIHRVTGSVIIDFEPVKPHEVPMTAAGALQSYKFAAKAITRLQSLPSGNMDTLCDTMVREVFELTGYDRVMAYKFHEDDHGEVIAEVKRPDLEPYLGLHYPATDIPQATRFLFMKNKVRMIVDCRAKHVKVLQDKKIPFELTLCGSTLRAAHSCHLQYMQNMNSSASLVMAVVVNDNDEDGDSSDAVQPQKRKRLWGLVVCHHSTPRFVPFPLRYACEFLAQVFAIHVNKELEIEYQIIEKNILRTQTLLCDMLMRDAPLGIVSQSPNIMDLVKCDGAALLYKNKVWRLGVTPSESQVREIALWLSECHRDSTGLSTDSLSDAGFPGTATLGDITCGMAAVRISSKDIVFWFRSHTAAEIRWGGAKHEPGERDDGSRMHPRSSFKAFLEVVKTRSLPWKDYEMDVIHSLQLILRNAFKDNESMEISTYAINTRLDDLKIEGMQELEAVTSEMVRLIETATVPILAVDVNGMVNGWNTKIAELT

>QCD98374.1 phytochrome A [Vigna unguiculata]

MMSFDKVHSSFASVSEQSNRFRSSLRDINLVILCLWFISSAVEVTMSSSRPSQSSSNNSGRSKSSRRSARVLAQTTLDAKLHATFEESGSSFNYSNSVRLSPGTGTASGDHQSRSDEVTTAYLHQLQKSKLIQPFGCLLALDEKTCKVIAYSENAPEMLTMVSHAVPSVGEHPALGIGTDIRTIFTAPSSTSIQKALGFGEVSLLNPILVHCKSSGKPFYAIIHRVTGSVIIDFEPVKPHEVPMTAAGALQSYKFAAKAITRLQSLPSGNMDTLCDTMVREVFELTGYDRVMAYKFHEDDHGEVIAEVKRPDMEPYLGLHYPATDIPQATRFLFMKNKVRMIVDCRAKHVKVLQDKRIPFELTLCGSTLRAAHSCHLQYMQNMNASASLVMAVVVNDNDEDGDSSDAVQPQKRKRLWGLVVCHHSTPRFVPFPLRYACEFLAQVFAIHVNKELEIEYQIIEKNILRTQTLLCDMLMRDAPLGIVSQSPNIMDLVKCDGAALLYKNKLWRLGVTPSESQIREIALWLSECHRDSTGLSTDSLSEAGFPGAATLGDITCGMAAVRISSKDIVFWFRSHTAAEIRWGGAKHEPGERDDGSRMHPRSSFKAFLEVVKTRSLPWKDYEMDVIHSLQLILRNAFKDNESMEISTYAINTRLGDLKIEGMQELEAVTSEMVRLIETATVPILAVDVNGMVNGWNTKIAELTCLPVEQAIGKHLLTLVEDFSVDRVKKMLDMALQGEEEKNVQFEIKTHDLKIDSGPISLVVNACASRDLQDNVVGVCFVAQDITAQKTVMDKFTRIEGDYKAIVQNPNPLIPPIFGTDEFGWCCEWNSAMTKLTGWKREEVMDKMLLGEVFGTHIACCRLRNQEAVVNFSIVLNKAMAGLETEKVPLSFITRDGKHVECLLSVSKKLDAEGVVTGVFCFLQLASPELQQALHIQHLSEQTASKRLKALTYLKRQIRSPLYGIVFSRKLLEGTELGAEQKQFLQTGTRCQRQLSKVLDDSDLDSIIDGYLDLEMVEFTLHEVLVSSLSQALTKGNAKGVRVVNDVEEQ

ITTETLYGDGLRLQQVLADFLLVSINSTPTGGQVVVAASLTKQQLGKLVHLANLELSITHDGFGVPETLLNQMFGRDGDESEEGISMLISRKLLKLMNGEVRYIREAGNSSFILSVELAAAHKSNTSNFGNN
